# Dysregulated lipid metabolism and hypomyelination in postnatal peroxisome-deficient *Pex2* knockout Zellweger mice

**DOI:** 10.3389/fnmol.2026.1636268

**Published:** 2026-02-24

**Authors:** Tanja Eberhart, Khanichi N. Charles, Brenda Salumbides-Torres, Nia Price, Steven J. Fliesler, Phyllis L. Faust, Werner J. Kovacs

**Affiliations:** 1 Institute of Molecular Health Sciences, ETH Zurich, Zurich, Switzerland; 2 Department of Biology, San Diego State University, San Diego, CA, United States; 3 Departments of Ophthalmology and Biochemistry and Graduate Program in Neuroscience, University at Buffalo-The State University of New York (SUNY), Buffalo, NY, United States; 4 Research Service, Veterans Administration Western New York Healthcare System, Buffalo, NY, United States; 5 Department of Pathology and Cell Biology, Vagelos College of Physicians and Surgeons, Columbia University, New York, NY, United States

**Keywords:** brain, central nervous system, cholesterol, fatty acids, myelination, peroxisomes, proteomics, Zellweger syndrome

## Abstract

Peroxisomes are dynamic organelles that play a crucial role in cellular metabolism, particularly in fatty acid degradation, cholesterol homeostasis and reactive oxygen species metabolism. Their dysfunction is associated with severe neurological disorders, including Zellweger spectrum disorders (ZSD) and X-linked adrenoleukodystrophy (X-ALD). In this study, we investigated the relationship between cholesterol homeostasis and myelination in postnatal peroxisome-deficient *Pex2* knockout mice. We dissected the central nervous system (CNS) of 10-day-old (P10) control and *Pex2*^−/−^ mice into five regions: spinal cord, brainstem, cerebellum, diencephalon and cerebral cortex. Catalase activity, a marker enzyme of peroxisomes, was significantly increased in CNS regions of *Pex2*^−/−^ mice, indicating an oxidative imbalance. Proteomic analysis revealed significant alterations in peroxisomal proteins and pathways related to neurodegenerative diseases, cholesterol and fatty acid metabolism and mRNA processing. Cholesterol biosynthesis was particularly dysregulated: enzyme activities, mRNA, and protein levels were reduced in white matter regions but increased in the cerebral cortex. The elevated desmosterol levels in the brain of *Pex2*^−/−^ mice indicate impaired cholesterol synthesis. Sphingolipid metabolism was also altered in the peroxisome-deficient CNS, as the protein levels of enzymes dihydroceramide desaturase 1, ceramide synthase 2, fatty acid 2-hydroxylase, and UDP-glycosyltransferase 8 were significantly decreased. Myelination was significantly reduced throughout the CNS, as evidenced by decreased activities of the myelin marker 2′,3′-cyclic nucleotide 3′-phosphodiesterase (CNP) and decreased mRNA and protein levels of myelin-associated proteins. The consistent decrease in ribosomal protein S6 phosphorylation in the CNS of *Pex2*^−/−^ mice suggests that decreased mechanistic target of rapamycin complex 1 (mTORC1) activity contributes to hypomyelination. Gene expression analysis revealed an upregulation of pro-inflammatory cytokines and altered expression of some homeostatic and disease-associated microglial (DAM) genes. However, full DAM activation was not yet observed in *Pex2*^−/−^ mice at P10. In conclusion, this study shows that systemic peroxisome deficiency leads to severe hypomyelination and dysregulation of cholesterol and fatty acid metabolism in the CNS, providing new insights into the pathophysiology of peroxisomal disorders.

## Introduction

Peroxisomes are ubiquitous and highly dynamic organelles whose number, size, and function depend on cell type and metabolic needs. They play key roles in fatty acid degradation [i.e., very long-chain fatty acids (VLCFAs), branched-chain FAs, polyunsaturated FAs (PUFAs)], ether lipid (i.e., plasmalogens), cholesterol and bile acid synthesis, and ROS metabolism (Van Veldhoven, 2010; Faust and Kovacs, 2014; Wanders et al., 2023). They also act as intracellular signaling platforms in redox, lipid, inflammatory and innate immune signaling (Nordgren and Fransen, 2014; Fransen and Lismont, 2019; Di Cara et al., 2023). The importance of peroxisomes for cellular metabolism is illustrated by the marked abnormalities in brain and systemic organs in peroxisome biogenesis disorders of the Zellweger spectrum disorders (ZSD; Zellweger syndrome, neonatal adrenoleukodystrophy, infantile Refsum disease), which lack functional peroxisomes, and disorders caused by single peroxisomal enzyme/protein deficiencies [e.g., X-linked adrenoleukodystrophy (X-ALD)] (Raymond et al., 2009; Wanders et al., 2023). X-ALD, caused by mutations in the *ABCD1* (ATP Binding Cassette Subfamily D Member 1) gene, is the most frequent peroxisomal disorder (Moser et al., 2007; Berger et al., 2014). The lack of peroxisomal metabolism results in severe biochemical abnormalities, leading to a variety of clinical symptoms both in patients with peroxisomal disorders and in peroxisome-deficient mice (Kovacs et al., 2002; Baes and Van Veldhoven, 2012; Van Veldhoven and Baes, 2013; Faust and Kovacs, 2014; Klouwer et al., 2015).

Peroxisomes are more abundant in the developing central nervous system and are also biochemically diverse, leading to functional differences among different cell types and brain regions (Arnold and Holtzman, 1978; Adamo et al., 1986; Lazo et al., 1991; Knoll et al., 2000; Kovacs et al., 2001; Ahlemeyer et al., 2007). Quantitative analysis revealed a comparable abundance of peroxisomes in cultured neurons and astrocytes isolated from the medial neocortex, hippocampus and cerebellum of newborn mice. In contrast, catalase immunoreactivity was higher in cultured astrocytes than in neurons. In mouse brain tissue, peroxisome abundance decreased by half during postnatal development, with marked differences between different brain regions and cell types (Ahlemeyer et al., 2007). In the CNS, peroxisomes are generally more abundant in differentiating compared to mature neurons and are found at sites such as axon terminals and dendrites while they are rare in the mature nervous system (Holtzman et al., 1973; McKenna et al., 1976; Arnold and Holtzman, 1978). Peroxisomes are also involved in the early determination of neuronal polarity, and it has been suggested that they are one of the constituents of the polarized cytoplasmic flow preceding axonogenesis (Bradke and Dotti, 1997). In the CNS of Zellweger infants, neuronal migration is disrupted, resulting in characteristic cytoarchitectural abnormalities involving the cerebral hemispheres, cerebellum, and inferior olivary complex (Volpe and Adams, 1972; Evrard et al., 1978; Faust et al., 2005; Barry and O’Keeffe, 2013). The four mouse models for Zellweger syndrome (*Pex2*^−/−^, *Pex5*^−/−^, *Pex11b*^−/−^, *Pex13*^−/−^) have severe defects in neuronal migration in both the cerebrum and cerebellum and malformation of the inferior olivary complex, as well as extensive neuronal death by apoptosis (Baes et al., 1997; Faust and Hatten, 1997; Faust et al., 2001, 2005; Li et al., 2002; Faust, 2003; Janssen et al., 2003; Maxwell et al., 2003; Kassmann et al., 2007; Krysko et al., 2007; Hulshagen et al., 2008; Bottelbergs et al., 2010, 2012; Müller et al., 2011; Rahim et al., 2014).

In the CNS, peroxisomes are particularly abundant in myelin-forming oligodendrocytes prior to the appearance of myelin sheaths and for several days thereafter (Adamo et al., 1986; Kassmann et al., 2011). During rat brain development, peroxisomal activity, represented by catalase activity, remained constant in the cerebral cortex (a typical gray matter region). However, in the white matter, the activity changed over time, with a clear peak accompanying the phase of myelination (during postnatal days 17–31) (Adamo et al., 1986). Subsequently, the overall abundance of peroxisomes and the rate of lipid precursor synthesis decrease. A similar increase in catalase activity was found in extracts of mouse spinal cord, brainstem, and cerebellum (Kovacs et al., 2001). A systematic comparison using Western blot analysis and catalase activity measurements found the maximum level 2 days after birth, and at later timepoints, 15 and 49 days postnatally, the levels of peroxisomal enzymes remained comparable (Ahlemeyer et al., 2007).

The brain is the most cholesterol-rich organ in the body, containing ~25% of unesterified cholesterol in the whole body. The majority of cholesterol in the CNS is found in two distinct pools: one comprising myelin sheaths (i.e., oligodendroglia) and the other consisting of the plasma membranes of astrocytes and neurons (Snipes and Suter, 1997; Saher and Stumpf, 2015; Montani, 2021). Within the brain, cholesterol is predominantly found in the white matter, and up to 70% of brain cholesterol is associated with myelin, where it is required for proper function of cell membranes, regulation of ion permeability, and proper structure and function of myelin proteins (Snipes and Suter, 1997). A characteristic of myelin is that it consists of 70% lipids and 30% proteins (when related to its dry weight), which is approximately the opposite of most other cell membranes. Myelin contains cholesterol, phospholipids, and glycosphingolipids (particularly galactocerebrosides) in molar ratios ranging from 4:3:2 to 4:4:2 (Baumann and Pham-Dinh, 2001; Barnes-Vélez et al., 2023). Plasmalogens, which are partially synthesized in peroxisomes, comprise over 30% of total phospholipids found in myelin-rich white matter and their levels in the brain increase postnatally in parallel with myelination (Barnes-Vélez et al., 2023). In addition to being a structural component of cell membranes, neurons need cholesterol for the development and maintenance of axons, dendrites and synaptic connections (Nieweg et al., 2009).

The brain depends on intracerebral *de novo* synthesis of cholesterol because the blood–brain barrier effectively prevents low-density lipoprotein (LDL) receptor-mediated uptake of cholesterol from the circulation. It has been shown that local synthesis is sufficient to account for over 95% of total brain sterol (desmosterol and cholesterol) (Jurevics and Morell, 1995; Dietschy and Turley, 2001, 2004). Most of the sterol is acquired during myelination in the early stages of development, and the rate of cholesterol synthesis in the developing CNS is relatively high and declines to very low levels in the adult state, probably reflecting cholesterol synthesis primarily in astrocytes (Spady and Dietschy, 1983; Quan et al., 2003; Dietschy and Turley, 2004; Li et al., 2022). It has been reported that the activity of 3-hydroxy-3-methylglutaryl CoA reductase (HMGCR), the rate-limiting enzyme in cholesterol synthesis, is highest during the early phase of ontogenetic brain development (Maltese and Volpe, 1979a, 1979b; Volpe et al., 1985). Oligodendrocytes, the cells responsible for myelination, have the highest rate of cholesterol synthesis in the CNS (Snipes and Suter, 1997). A recent study using cholesterol-deficient oligodendrocytes showed that cholesterol is an indispensable component of myelin membranes and that cholesterol availability in oligodendrocytes is a rate-limiting factor for brain maturation (Saher et al., 2005). However, cholesterol-deficient oligodendrocytes were able to acquire cholesterol, presumably from neighboring astrocytes via apoE and LDL receptor related protein (LRP) or other undefined mechanisms (Saher et al., 2005). Astrocytes, the most abundant glial cells, secrete cholesterol in lipoprotein particles (Boyles et al., 1985; Shanmugaratnam et al., 1997) and have been proposed to provide cholesterol for synapse formation (Mauch et al., 2001) and to participate in cholesterol recycling after injury (Danik et al., 1999). Neurons are capable of synthesizing cholesterol, but it has been suggested that they reduce or abandon cholesterol biosynthesis in the adult state and outsource it to astrocytes (Pfrieger, 2003). Cholesterol biosynthesis consumes large amounts of energy metabolites, and outsourcing may be more cost effective for neurons that specialize in generating electrical activity. The brain has the highest concentration of VLCFAs and it has been shown that acetyl-CoA derived from peroxisomal β-oxidation of VLCFAs is preferentially used for sterol synthesis (Kovacs et al., 2007).

Despite the efficiency of the cholesterol recycling machinery in the brain (e.g., cholesterol has a half-life of 4–6 months in rat brain, ~1 year in mice, ~5 years in human) (Berghoff et al., 2022), there is a constant need to export a small excess of cholesterol into the circulation to maintain steady state. Data suggest that cholesterol metabolism is very different in different compartments of the CNS, with extremely high turnover rates in some types of neurons, much lower rates in glial cells, and very low turnover rates in myelin (Dietschy and Turley, 2004). A minor pathway is the clearance of apoE-bound cholesterol via the cerebrospinal fluid. The quantitatively important mechanism involves the conversion of cholesterol to 24(S)-hydroxycholesterol (24S-OH) by cholesterol 24-hydroxylase (CYP46A1) (Lund et al., 1999, 2003). The latter oxysterol readily crosses the blood–brain barrier and is delivered to the liver for excretion in bile. CYP46A1 is expressed exclusively in neurons and in the areas of the CNS with the largest volumes of gray matter. 24S-OH is an inhibitor of cholesterol synthesis and a potent activator of liver X-activated receptors (LXRs) and stimulates cholesterol efflux from astrocytes, a mechanism that involves activation of the cholesterol transporter ABCA1 (ATP-binding cassette subfamily A member 1) and ABCG1 (Wang and Eckel, 2014; Courtney and Landreth, 2016).

Systemic knockout mouse models for the peroxins *Pex2* (Faust and Hatten, 1997), *Pex5* (Baes et al., 1997), and *Pex13* (Maxwell et al., 2003) have been generated as models for Zellweger syndrome. In these three models, newborn pups are growth retarded, severely hypotonic, do not feed, and die 6–24 h after birth. Therefore, these systemic knockout mouse models are not suitable for studying myelination, which occurs postnatally in the first 3 weeks of life. However, studies with cell-type-specific *Pex5* and *Pex13* knockout mice have provided valuable insights into the cell-autonomous roles of peroxisomes in myelination, axonal integrity, and neuroprotection, but they do not fully replicate the pathophysiology of human ZSD (Kassmann et al., 2007; Krysko et al., 2007; Hulshagen et al., 2008; Bottelbergs et al., 2010, 2012; Müller et al., 2011; Kassmann, 2014; Berger et al., 2016).

20–30% of *Pex2*^−/−^ mice on a mixed Swiss Webster × 129SvEv genetic background (SW/129) survive for about 1–2 weeks (Faust and Hatten, 1997; Kovacs et al., 2004, 2009), and the postnatal survival can be improved by oral bile acid therapy (9% alive at 30 days) (Keane et al., 2007). Abnormalities in cholesterol homeostasis were observed in the liver and extrahepatic tissues of 9–10-day-old (P9–10) SW/129 *Pex2*^−/−^ mice (Kovacs et al., 2004, 2009). The mRNA, protein and activity levels of cholesterol biosynthetic enzymes were increased in liver and extrahepatic tissues, resulting in an enhanced rate of cholesterol synthesis from [^3^H]acetate *in vivo* (Charles et al., 2020; Kovacs et al., 2004, 2009). This was orchestrated by an upregulation of the sterol regulatory element-binding protein 2 (SREBP-2) pathway. However, liver and plasma cholesterol levels were reduced by approximately 40% in *Pex2*^−/−^ mice, while cholesterol levels in other tissues were similar between control and *Pex2*^−/−^ mice. Bile acid feeding of *Pex2*^−/−^ mice significantly attenuated the activities, protein and mRNA levels of cholesterol biosynthetic enzymes, decreased hepatic cholesterol synthesis, and normalized hepatic cholesterol levels (Kovacs et al., 2009). We have shown that peroxisome deficiency in P0 and postnatal *Pex2*^−/−^ mice activates ER stress pathways, in particular the integrated stress response mediated by PERK and ATF4 signaling, leading to dysregulation of the SREBP-2 pathway (Kovacs et al., 2009, 2012; Faust and Kovacs, 2014).

The aim of the present study was to investigate the relationship between cholesterol homeostasis and myelination in postnatal peroxisome-deficient *Pex2* knockout mice. As ZSD is a systemic disease involving a peroxisome biogenesis defect in all cell types, its pathological phenotype reflects interactions between multiple cell types and tissues. Furthermore, the process of myelination is not solely the responsibility of oligodendrocytes, as neurons, astrocytes and microglia also influence oligodendrocyte function and therefore the myelination process. Therefore, postnatal *Pex2*^−/−^ mice, which have a systemic deficiency of functional peroxisomes, are expected to better reflect the situation in ZSD patients. For this purpose, we dissected the CNS of P10 control and *Pex2*^−/−^ mice into five regions including the spinal cord, brainstem, cerebellum, diencephalon, and cerebral cortex. First, we performed mass spectrometry-based differential quantitative proteomic analyses of these CNS regions, followed by pathway analyses. Subsequently, we measured the activity of cholesterol biosynthesis enzymes and conducted an mRNA analysis of cholesterol and fatty acid biosynthesis enzymes and myelin-associated proteins.

## Materials and methods

### Animal studies

Homozygous *Pex2*^−/−^ mice were obtained by breeding *Pex2* heterozygote mice on a hybrid Swiss Webster-129 (SW/129) background (Faust et al., 2001). *Pex2* mice and BALB/c mice had *ad libitum* access to food and water and were exposed to a 12:12 h light–dark cycle. As their biochemical characteristics were comparable, the control mice consisted of either *Pex2*^+/+^ or *Pex2*^+/−^ genotypes (hereafter referred to as *Pex2*^+/^) for the purpose of this study (Kovacs et al., 2004, 2009). A RT-PCR-based method targeting *Kdm5c* and *Kdm5d* was used to determine the genetic sex in mouse cDNA samples (Bosso-Lefèvre et al., 2023). Sex was not considered a biological variable in 10-day-old (P10) pups, so both male and female animals were examined in our study. Bile acids (BAs) were administered by orogastric gavage as described previously (Keane et al., 2007). All animal experiments and protocols were approved by the Institutional Animal Care and Use Committee of San Diego State University and Columbia University.

### Western blot analysis

Frozen tissues (1:10 w/v) were homogenized in RIPA buffer (20 mM Tris, pH 7.5; 150 mM NaCl; 1 mM EDTA; 1 mM EGTA; 1% NP-40; 1% sodium deoxycholate) containing the cOmplete protease inhibitor cocktail (Roche; #11697498001) and the PhosSTOP phosphatase inhibitor (Roche; #4906837001) using the Potter S homogenizer (Sartorius, Goettingen, Germany). Homogenates were incubated on ice for 30 min and centrifuged at 20,000 g for 20 min at 4 °C. Protein concentration was determined by the BCA method (Thermo Fisher Scientific; #23225). Equal amounts of proteins were subjected to SDS-polyacrylamide gel electrophoresis and transferred to Amersham Protran Supported 0.2 μm nitrocellulose (Cytvia; GE10600015). After blocking for 1 h in TBST [Tris-buffered saline with 0.05% Tween-20] containing 1% bovine serum albumin (BSA) (PAN Biotech; P06-1391500), membranes were probed with the indicated primary antibodies in TBST containing 1% BSA overnight at 4 °C. The membranes were incubated with horseradish peroxidase-conjugated secondary antibodies and developed using the Clarity Western ECL Substrate (BIO-RAD; Cat#1705061). Blots were either exposed to Kodak X-Omat LS film (Rochester, NY) and scanned using a densitometer (GE Healthcare) or visualized on the Fusion Solo S imaging system. Blots were analyzed semiquantitatively by densitometry using ImageJ 1.52 v (National Institutes of Health). Antibodies are listed in Supplementary Table S1.

### Enzyme assays

Catalase (EC 1.11.1.6) activity was assayed as described previously (Walter et al., 2014). Briefly, protein lysates were diluted with TVBE buffer [0.01% (v/v) Triton X-100 (Sigma-Aldrich; #93426), 1 mM EDTA, 1 mM NaHCO_3_, 0.1% (v/v) ethanol; pH 7.5]. Equal amounts of sample and 2% Triton X-100 were added to glass test tubes on ice and incubated for 1 min. The reagent control contained TVBE buffer and 2% Triton X-100. The reaction was started by adding 1 mL cold substrate solution (20 mM imidazole, 0.1% fatty acid-free BSA (PAN Biotec; P06-139450) and 3.1 mM H_2_O_2_ (Sigma-Aldrich; #216763) at pH 7.0) and the samples were incubated at 0 °C. The enzymatic reaction was stopped by adding 1 mL TiOSO_4_ solution. The glass tubes were transferred to room temperature (RT) and incubated for 10 min for full color development. Absorbance was measured at 410 nm. Catalase activity was measured as follows: BU/mL = (1 + *x* mL Sample + *x* mL TX-100)/50 * 1/(incubation time in min) * 1/(sample volume in mL) * log (reagent absorbance/sample absorbance) * dilution factor.

The enzyme 2′,3′-cyclic nucleotide 3′-phosphodiesterase (CNP, EC 3.1.4.37), a marker enzyme for myelin, was assayed as described previously (Kovacs et al., 2001). HMG-CoA reductase (HMGCR, EC 1.1.1.34), farnesyl pyrophosphate synthase (FDPS, EC 2.5.1.10), isopentenylpyrophosphate isomerase (IDI1, EC 5.3.3.2), and squalene synthase (farnesyl-diphosphate farnesyltransferase 1, FDFT1, EC 2.5.1.21) activities were assayed as described previously (Kovacs et al., 2004).

### Brain sterol analysis

Brain sterols were measured as described previously (Kovacs et al., 2004). Briefly, the mice were euthanized, their tissues harvested and flash frozen in liquid nitrogen before being stored at −85 °C until ready for analysis. After saponification and petroleum ether extraction, the nonsaponifiable lipids were analyzed by reverse-phase radio-HPLC, and the specific activities of the radiolabeled products were determined.

### RNA isolation, northern blot analysis and quantitative RT-PCR

Total RNA was isolated and Northern blot analysis was performed as previously described (Kovacs et al., 2004, 2009). Quantitative RT-PCR assays were performed as described previously (Charles et al., 2020). First-strand cDNA was synthesized with random hexamer primers using the High-Capacity cDNA Reverse Transcription Kit (Applied Biosystems; #4368813). Quantitative RT-PCR was performed on a Roche LightCycler LC480 instrument in duplicate using 10 ng cDNA, 2 pmol forward and reverse primers, and the 2× KAPA SYBR FAST qPCR Mastermix (Roche; #KK4611). Thermal cycling was performed with a 5 min denaturation step at 95 °C, followed by 45 three-step cycles: 10 s at 95 °C, 10 s at 60 °C, and 10 s at 72 °C. Melt curve analysis was performed to confirm the specific amplification of a target gene and absence of primer dimers. Primer sequences are listed in Supplementary Table S2 or have been previously published (Charles et al., 2020). Expression levels were calculated using the $2^{–\Delta \Delta C_{T}}$ method (Livak and Schmittgen, 2001). *18S* rRNA or *Cyclophilin* were used as the invariant control.

The *Cnp* cDNA (GenBank accession number NM_009923) was prepared from total mouse brain RNA by a standard reverse transcriptase-PCR procedure using the following forward and reverse primers: 5′-TACCCGCAAAAGCCACACAT-3′ and 5′-TCAGCATCCATCGCCCTTTG-3′. The PCR-generated probe was subcloned into the pCRII-TOPO vector (Invitrogen) and confirmed by sequencing. IMAGE clones for proteolipid protein 1 (*Plp1*; ID 1617043) and myelin basic protein (*Mbp*; ID 3982852) were purchased from ATCC.

### Immunohistochemistry

Indirect immunofluorescence on 2 μm paraffin sections was performed with the indicated antibodies (Supplementary Table S1) as described previously (Kovacs et al., 2012). Mice were cardiac-perfused with 4% paraformaldehyde-phosphate-buffered saline. The brain was post-fixed overnight in 4% paraformaldehyde-phosphate-buffered saline and processed for paraffin embedding. 2 μm thick paraffin-embedded brain sections were mounted on Superfrost Plus slides. For antigen retrieval of paraffin-embedded tissue, deparaffinized and rehydrated sections were digested with 0.01% trypsin (Sigma T7409; Type II-S from porcine pancreas) for 10 min at 37 °C followed by microwaving in 10 mM citrate buffer (pH 6.0) three times for 5 min at 800 watts. Nonspecific binding was blocked with 4% BSA and 0.05% Tween 20 in PBS for 2 h and sections were then incubated overnight at 4 °C with primary antibodies. Secondary antibodies were applied for 2 h. Negative control sections were incubated in parallel by omitting the primary antibody. Images were taken with a Leica SP2-AOBS confocal laser scanning microscope. Fluorescent dyes were imaged sequentially in frame interlace mode to eliminate crosstalk between the channels.

### Proteomic analysis

Tissue samples were transferred to 2 mL Eppendorf tubes and 200 μL lysis buffer [4% sodium dodecyl sulfate (SDS) in 50 mM triethylammoniumbicarbonat (TEAB), pH 8.2] was added per sample. Protein extraction was carried out using a tissue homogenizer (TissueLyser II, QIAGEN) by applying 2× 2 min cycles at 30 Hz. Samples were treated with High Intensity Focused Ultrasound for 1 min at an ultrasonic amplitude of 90% before boiling at 95 °C for 10 min while shaking at 800 rpm on a Thermoshaker (Eppendorf). Protein concentration was determined using the Lunatic UV/Vis polychromatic spectrophotometer (Unchained Labs) at a 1:10 dilution.

For each tissue sample, 50 μg of protein was taken and reduced with 5 mM TCEP [tris(2-carboxyethyl)phosphine] and alkylated with 15 mM chloroacetamide at 30 °C for 30 min. Samples were processed using single-pot solid phase enhanced sample preparation (SP3). The SP3 protein purification, digestion and peptide clean-up were performed using a KingFisher Flex System (Thermo Fisher Scientific) and carboxylate-modified magnetic particles (GE Life Sciences; GE65152105050250, GE45152105050250) (Hughes et al., 2014; Leutert et al., 2019). Beads were conditioned following the manufacturer’s instructions, consisting of 3 washes with water at a concentration of 1 μg/μl. Samples were diluted with 100% ethanol to a final concentration of 60% ethanol. Beads, wash solutions and samples were loaded into 96 deep well- or micro-plates and transferred to the KingFisher. The following steps were performed on the robot: collection of beads from the last wash, protein binding to the beads, washing of the beads in wash solutions 1–3 (80% ethanol), protein digestion (overnight at 37 °C with a trypsin:protein ratio of 1:50 in 50 mM TEAB), and peptide elution from the magnetic beads using MilliQ water. The digest solution and water elution were combined, dried to completeness, and re-solubilized in 20 μL of MS sample buffer (3% acetonitrile, 0.1% formic acid). The peptide concentration was determined using the Lunatic UV/Vis polychromatic spectrophotometer.

LC-MS/MS analysis was performed on an Orbitrap Fusion Lumos (Thermo Scientific) equipped with a Digital PicoView source (New Objective) and coupled to an M-Class UPLC (Waters). The solvent composition of the two channels was 0.1% formic acid for channel A and 99.9% acetonitrile in 0.1% formic acid for channel B. The column temperature was 50 °C. For each sample, 300 ng of peptides were loaded onto a commercial ACQUITY UPLC M-Class Symmetry C18 Trap Column (100 Å, 5 μm, 180 μm × 20 mm, Waters) connected to an ACQUITY UPLC M-Class HSS T3 Column (100 Å, 1.8 μm, 75 μm × 250 mm, Waters). Peptides were eluted at a flow rate of 300 nL/min. After an initial hold at 5% B for 3 min, a gradient of 5 to 22% B was applied for 80 min and 22 to 32% B for an additional 10 min. The column was cleaned at the end of the run by increasing to 95% B and holding at 95% B for 10 min before restoring the loading condition. Samples were measured in random order. The mass spectrometer was operated in data-dependent mode (DDA) with a maximum cycle time of 3 s, funnel RF level at 40%, heated capillary temperature at 275 °C, and advanced peak determination (APD) on. Full-scan MS spectra (300–1,500 m/z) were acquired at a resolution of 120,000 at 200 m/z after accumulation to an automated gain control (AGC) target of 500,000 or for a maximum injection time of 40 ms. Precursors with an intensity above 5,000 were selected for MS/MS. Ions were isolated using a quadrupole mass filter with a 0.8 m/z isolation window and fragmented by higher-energy collisional dissociation (HCD) using a normalized collision energy of 35%. Fragments were detected in the linear ion trap with the scan rate set to rapid, the automatic gain control set to 10,000 ions, and the maximum injection time set to 50 ms. Charge state screening was enabled, and singly, unassigned charge states and charge states greater than seven were excluded. Precursor masses previously selected for MS/MS measurement were excluded from further selection for 20 s with a mass tolerance of 10 ppm. Samples were acquired using internal lock mass calibration at m/z 371.1012 and 445.1200.

The mass spectrometry proteomics data were processed using the local laboratory information management system (LIMS) (Türker et al., 2010).

The acquired MS raw data were processed for identification and quantification using FragPipe (version 16.0), MSFragger (version 3.4), IonQuant (version 1.7.17), and Philosopher (version 4.1.1) (Yu et al., 2020). Spectra were searched against a Uniprot *Mus musculus* reference proteome (taxonomy 10090, canonical version from 2022-05), concatenated to its reversed decoyed fasta database and common protein contaminants. Strict trypsin digestion with a maximum of 2 missed cleavages was set for the closed search settings. Carbamidomethylation of cysteine was set as fixed modification, while methionine oxidation and N-terminal protein acetylation were set as variable. Label-free quantification and match between run option were enabled.

The R package prolfqua (Wolski et al., 2023) was used to analyze the differential expression and to determine group differences, confidence intervals, and false discovery rates for all quantifiable proteins. We started with the combined_protein.tsv file generated by FragPipe, which reports the protein abundances for each raw file. We then employed the Tukeys-Median Polish to estimate protein abundances. Furthermore, before fitting the linear models, we transformed the protein abundances using the variance stabilizing normalization (Huber et al., 2002).

For unsupervised multivariate statistical analysis, principal component analysis and hierarchical clustering was performed using Metaboanalyst 6.0 (Pang et al., 2024). Normalized protein abundance matrices of proteomic data derived from spinal cord, brainstem, cerebellum, and cerebral cortex samples from P10 control and *Pex2*^−/−^ mice were filtered for protein species occurring in all tissues, followed by left-censored missing data estimation (1/5th of the minimal positive value) and scaling (mean-centered and divided by the standard deviation of each variable). For hierarchical clustering, the Euclidean distance measurement and Ward clustering algorithm were applied.

All relevant data have been deposited to the ProteomeXchange Consortium via the PRIDE^1^ (Deutsch et al., 2023) partner repository with the dataset identifier PXD063585.

### Over-representation analysis

Over-representation analysis after proteomics was performed with Enrichr (RRID:SCR_001575) using the Gene ontology, KEGG and reactome databases (Ashburner et al., 2000; Xie et al., 2021; Milacic et al., 2024; Kanehisa et al., 2025). All proteins with an adjusted *p*-value <0.1 were included. No fold change threshold was set, as some of the proteins may show moderate but biologically relevant changes.

### Gene set enrichment analysis

Gene set enrichment analysis (GSEA) was performed using GSEA_4.3.2 software version (RRID:SCR_003199) (Subramanian et al., 2005). The software and all analyzed gene sets were downloaded from the official GSEA website (BROAD Institute).

### Statistical analyses

Data are presented as mean ± SD. Statistical analyses were performed with GraphPad Prism version 9.0. When two groups were compared, statistical significance was assessed by unpaired, two-tailed Student’s *t*-test or unpaired, two-tailed Student’s *t*-test with Welch’s correction when variances were significantly different for two-group analyses. To correct for multiple comparisons, the Benjamini–Hochberg procedure was applied in R to control the false discovery rate at 0.05 and calculate the adjusted *p*-values for all evaluated genes and tissues. For multiple group analyses, one-way ANOVA followed by Tukey’s multiple comparisons test or two-way ANOVA Tukey’s multiple comparisons test was used. Illustrations were created with Adobe Illustrator (v.27.2) and BioRender.

## Results

### Catalase in the CNS of *Pex2*^−/−^ mice

**Figure 1:**
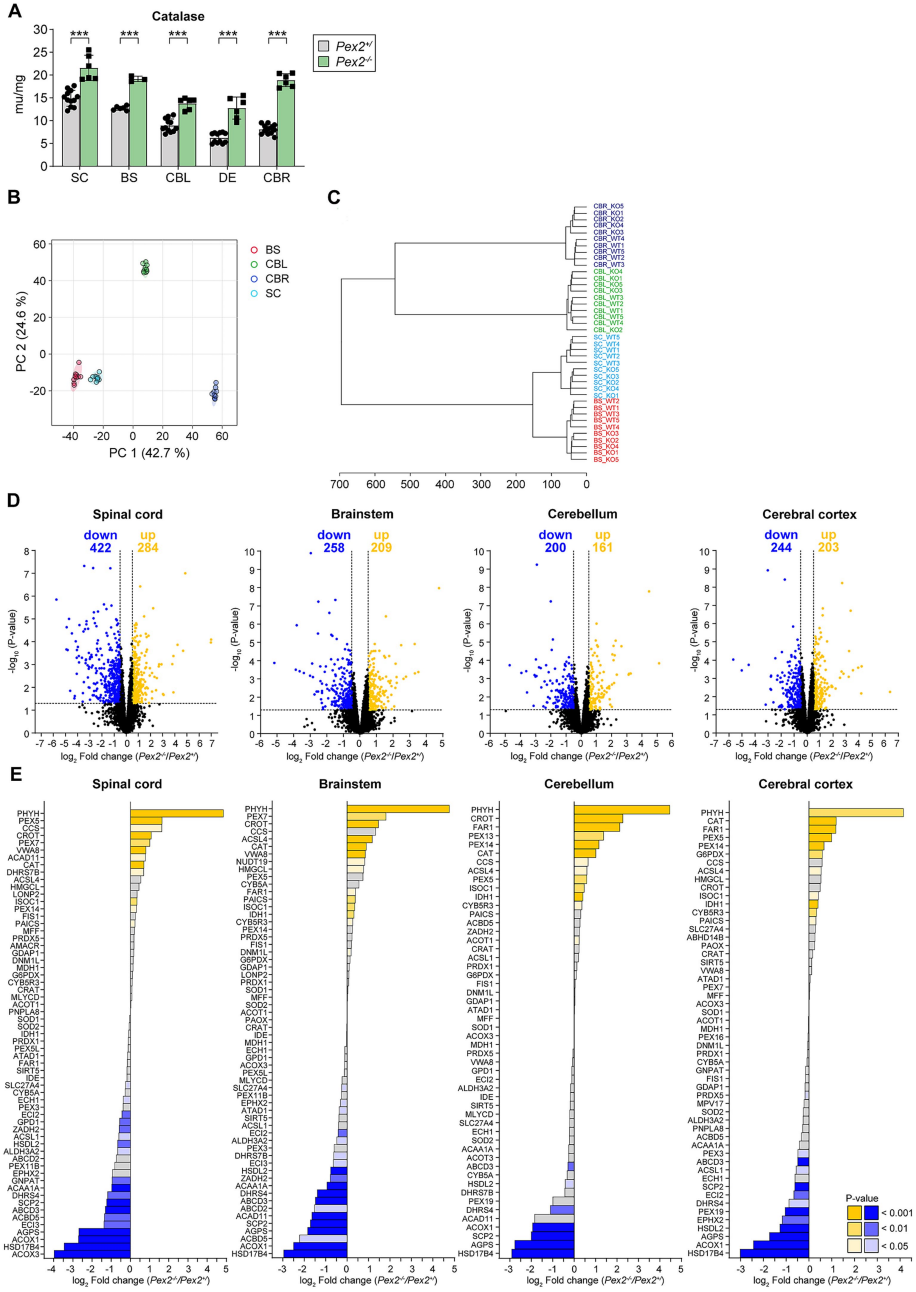
**(A)** Catalase activity in the spinal cord (SC), brainstem (BS), cerebellum (CBL), diencephalon (DE), and cerebral cortex (CBR) of P10 control (*Pex2*^+/^) and *Pex2*^−/−^ mice. Data are mean ± SD (*n* = 6–12 for control mice; *n* = 3–6 for *Pex2*^−/−^ mice). Statistical analysis was performed using Student’s *t*-test or Student’s *t*-test with Welch’s correction. ^***^p < 0.001 versus control mice. **(B,C)** Principal component analysis **(B)** and hierarchical clustering **(C)** of proteomics samples from SC (light blue), brainstem (red), cerebellum (green), and cerebral cortex (dark blue) of P10 control and *Pex2*^−/−^ mice. **(D)** Volcano plots of altered proteins in SC, BS, CBL, and CBR of P10 control and *Pex2*^−/−^ mice (*n* = 5). Cut-offs for log_2_ fold change and *p*-value were set at ±0.5 and 0.05, respectively. Blue dots: significantly downregulated proteins; yellow dots: significantly upregulated proteins; black dots: no significant change. Numbers of significantly up-or downregulated proteins are indicated in the plot. **(E)** Levels of bona fide and putative peroxisomal proteins in SC, BS, CBL, and CBR of P10 control and *Pex2*^−/−^ mice. Blue bars: significantly downregulated proteins; yellow bars: significantly upregulated proteins; gray bars: no significant change.

Catalase is the most abundant peroxisomal matrix protein and in the CNS the activity is highest during the first 3 weeks of development and then declines sharply (Kovacs et al., 2001; Ahlemeyer et al., 2007), supporting the hypothesis that the high peroxisomal activity in the CNS during the first weeks of postnatal life may be related to lipid synthesis associated with rapid myelin formation and the production of plasma membranes for growing neurons. First, we divided the CNS of wild-type BALB/c mice into five regions, namely spinal cord (SC), brain stem (BS), cerebellum (CBL), diencephalon (DE), and cerebral cortex (CBR), and measured the activity of catalase at different developmental stages [from 7-days-old (P7) to P56] (Supplementary Figure S1A). Catalase activity was highest at P7 and P14 and decreased significantly thereafter (Supplementary Figure S1A). The activity of the cholesterol biosynthetic enzyme FDPS was also highest at P7 and P14 and decreased significantly thereafter (Supplementary Figure S1B). Likewise, the CNS of P10 SW/129 control and *Pex2* knockout mice was divided into the SC, BS, CBL, DE, and CBR. In P10 control mice catalase activity was significantly higher in CNS regions containing a higher proportion of white matter (i.e., SC, BS) as compared to gray matter (i.e., DE, CBR) (Figure 1A). Catalase activity was significantly increased by ~44–133% in all examined regions of the CNS of *Pex2*^−/−^ mice at P10 (Figure 1A), with the highest increase in the DE and CBR (~2- and 2.3-fold, respectively). This finding suggests that peroxisome deficiency induces an oxidative imbalance that could activate stress pathways and lead to metabolic dysfunction.

### Changes in the CNS proteome of *Pex2*^−/−^ mice

To determine the impact of peroxisome deficiency on the CNS proteome, we characterized the proteome of the SC, BS, CBL, and CBR of P10 control and *Pex2*^−/−^ mice. Principal component analysis (PCA) (Figure 1B) and hierarchical clustering analysis (Figure 1C) revealed a clear separation between the proteomes of the different CNS regions. Furthermore, the hierarchical clustering analysis showed that the proteomes of each CNS region could be clearly separated based on genotype (Figure 1C). We detected 4,236, 4,243, 4,241, and 4,356 proteins in the SC, BS, CBL, and CBR, respectively (Supplementary Table S3). Among the detected proteins, 705, 467, 361, and 447 were significantly altered in SC, BS, CBL, and CBR of *Pex2*^−/−^ mice when the log_2_ fold change (FC) and *p*-value cut-offs were set at ±0.5 and 0.05, respectively (Figure 1D).

We have compiled a list of bona fide and putative peroxisomal proteins based on published studies and compared them with the proteins detected in our proteomics analysis (Kikuchi et al., 2004; Islinger et al., 2007; Mi et al., 2007; Wiese et al., 2007; Gronemeyer et al., 2013). We detected ~60 peroxisomal proteins in the CNS. As expected and confirming the defect in peroxisome biogenesis, bona fide peroxisomal matrix proteins involved in peroxisomal fatty acid β-oxidation (i.e., ACOX1, HSD17B4, SCP2), etherlipid synthesis (i.e., AGPS), and VLCFA transport (i.e., ABCD3) were strongly downregulated in *Pex2*^−/−^ mice (Figure 1E). This is because mislocalized matrix proteins in the cytosol are often unstable and targeted for degradation via the proteasome. Surprisingly, protein levels of the peroxisomal matrix protein phytanoyl-CoA 2-hydroxylase (PHYH), which is involved in peroxisomal α-oxidation and converts phytanoyl-CoA to 2-hydroxyphytanoyl-CoA, were highly increased in all CNS regions examined in *Pex2*^−/−^ mice (Figure 1E). Several studies have shown that patients with ZSD have reduced levels of PHYH and increased levels of phytanic acid in their plasma and tissues (Aubourg et al., 1985; Schutgens et al., 1987; Molzer et al., 1989; Jansen et al., 1996; Verhoeven et al., 1997; Verhoeven and Jakobs, 2001). Little is known about how PHYH protein levels are regulated, but it is thought that phytanic acid or its precursor, phytol, could lead to increased levels of PHYH. A study found that PHYH activity is induced by phytanic acid, but this induction was not regulated via PPARα or RXR (Zomer et al., 2000). We determined the mRNA expression of *Phyh* and found no differences between P10 control and *Pex2*^−/−^ mice in any of the regions of the CNS examined (Supplementary Figure S1C), suggesting that PHYH protein levels in the CNS are controlled post-translationally. Peroxins (PEX), which are involved in the biogenesis of peroxisomes, showed a mixed picture, as the protein levels of some peroxins were reduced or increased. For example, the protein levels of PEX14 were increased in *Pex2*^−/−^ mice (Figure 1E). Peroxins can localize to peroxisomal membrane ghosts in *Pex2*^−/−^ mice or cells (Kovacs et al., 2012; Charles et al., 2020), and PEX14 has been reported to localize to mitochondria in PEX3-deficient mammalian cells (Muntau et al., 2000; South et al., 2000; Sugiura et al., 2017; Jansen et al., 2024). The protein levels of PEX5, which is predominantly cytoplasmic and required for the targeting of peroxisomal matrix proteins to peroxisomes, were significantly increased in all CNS regions of *Pex2*^−/−^ mice except for the brainstem.

**Figure 2:**
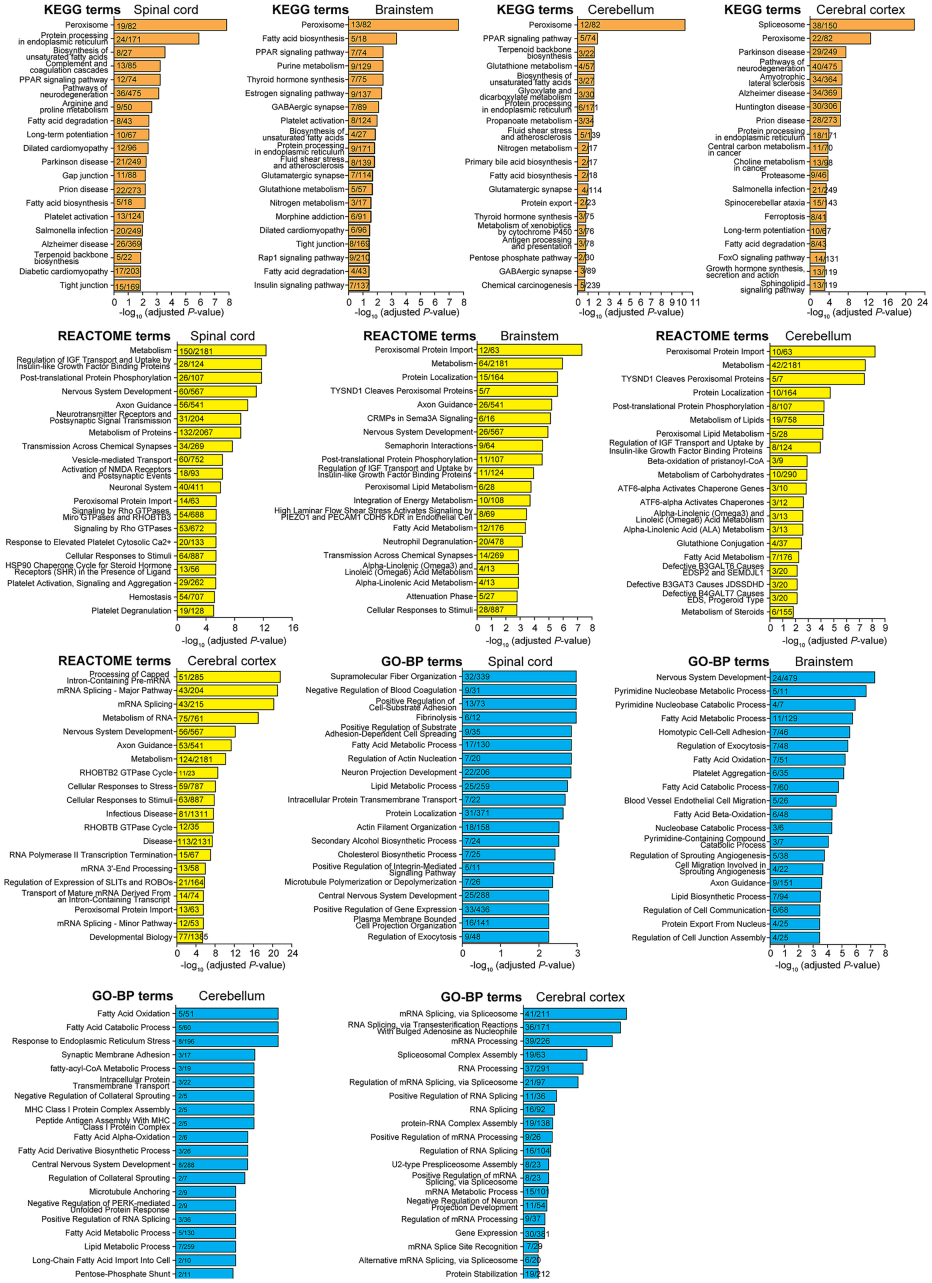
Functional analysis of the identified proteins. ORA analysis of differentially expressed proteins in SC, BS, CBL, and CBR of P10 control and *Pex2*^−/−^ mice using the KEGG, REACTOME, and gene ontology biological process (GO-BP) databases. All proteins with an adjusted *p*-value <0.1 were included. No fold change threshold was set, as some of the proteins may show moderate but biologically relevant changes. Numbers in bars indicate differentially expressed proteins compared to the number of proteins in each term.

Next, we performed an overrepresentation analysis (ORA) to investigate which specific biological processes were enriched in differentially expressed proteins in the CNS of control versus *Pex2*^−/−^ mice (Figure 2; Supplementary Figure S2 and Supplementary Table S4). As expected, ORA confirmed that peroxisome-associated proteins were significantly altered in *Pex2*^−/−^ mice. Importantly, an overrepresentation of processes involved in neurodegenerative diseases (Parkinson’s disease, Alzheimer disease, Amyotrophic lateral sclerosis, prion disease, pathways of neurodegeneration, Huntington disease, Spinocerebellar ataxia), where neuronal death and dysfunction are central themes, was observed in the top pathways in *Pex2*^−/−^ mice. The overrepresentation of neurodegenerative disease pathways confirms studies showing severe neurological dysfunction and abnormal neuronal migration in the developing cerebral cortex and cerebellum of *Pex2*^−/−^ mice (Faust and Hatten, 1997; Faust et al., 2001, 2005; Faust, 2003). It also suggests that peroxisome dysfunction contributes to the pathophysiology of the above-mentioned neurodegenerative diseases. GSEA of the proteomics data showed that the SC and BS of *Pex2*^−/−^ mice had a significant positive enrichment score for “Neuronal system,” whereas the CBR and CBL had a negative enrichment score (Supplementary Figure S3A and Supplementary Table S5). The significantly altered proteins under this term indicate changes in neurotransmission, neuronal excitability, synaptic plasticity, glutamate metabolism, and response to oxidative stress (Supplementary Table S5). Pathways of fatty acid degradation and biosynthesis, such as fatty acid biosynthesis, PPAR signaling, and unsaturated fatty acid biosynthesis, were overrepresented in *Pex2*^−/−^ mice, suggesting disruptions in cellular energy regulation and membrane composition that could affect neuronal and oligodendrocyte health.

Interestingly, ORA analysis revealed top terms related to mRNA processing (i.e., mRNA splicing and processing, RNA splicing, mRNA splicing via spliceosome, RNA splicing via transesterification reactions, regulation of mRNA splicing and mRNA processing, regulation of alternative splicing via spliceosome) (Figure 2; Supplementary Figure S2). These terms were particularly prevalent in the CBR. GSEA showed that the top GO terms that were most positively enriched for biological processes in the CBR and CBL of *Pex2*^−/−^ mice were related to RNA splicing and processing, as well as mRNA processing, metabolism, and splicing, and the regulation of RNA and mRNA splicing and processing (Supplementary Figure S3B and Supplementary Table S5). In addition, GSEA using gene sets from the Reactome database revealed significant positive enrichment for “Metabolism of RNA”, “mRNA processing and splicing”, and “Processing of capped intron-containing pre-mRNA”, particularly in the CBR and CBL of *Pex2*^−/−^ mice (Supplementary Figure S3B). The significantly altered proteins under these terms suggest enhanced RNA processing and splicing activity, enhanced RNA transport and stability, and increased transcriptional activity (Supplementary Table S5).

The top enriched GO term for molecular function was cadherin binding (Supplementary Figure S2). Cadherins are involved in regulating the interactions between cells and the extracellular matrix, which are essential for maintaining proper tissue architecture and cellular communication. More proteins are downregulated than upregulated under this term in the SC, BS, CBL, and CBR, indicating disrupted cytoskeletal dynamics and cell adhesion, altered vesicular trafficking, and impaired stress response (Supplementary Table S6). Cadherins are a class of cell adhesion molecules that play a critical role in neuronal development, synapse formation, and myelination (Payne et al., 1996; Schnädelbach et al., 2000; Laursen and Ffrench-Constant, 2007; Gärtner et al., 2015; Szabó et al., 2015; Chen et al., 2017; Martinez-Garay, 2020). Cadherins are involved in the communication between axons and oligodendrocytes during myelination, ensuring proper axonal growth and myelin formation. Cadherins are also involved in neuronal migration during development to facilitate the proper positioning of neurons. So far, no direct link between dysregulation of cadherins and peroxisomal disorders has been shown.

### Sterol levels in the brain of *Pex2*^−/−^ mice

**Figure 3:**
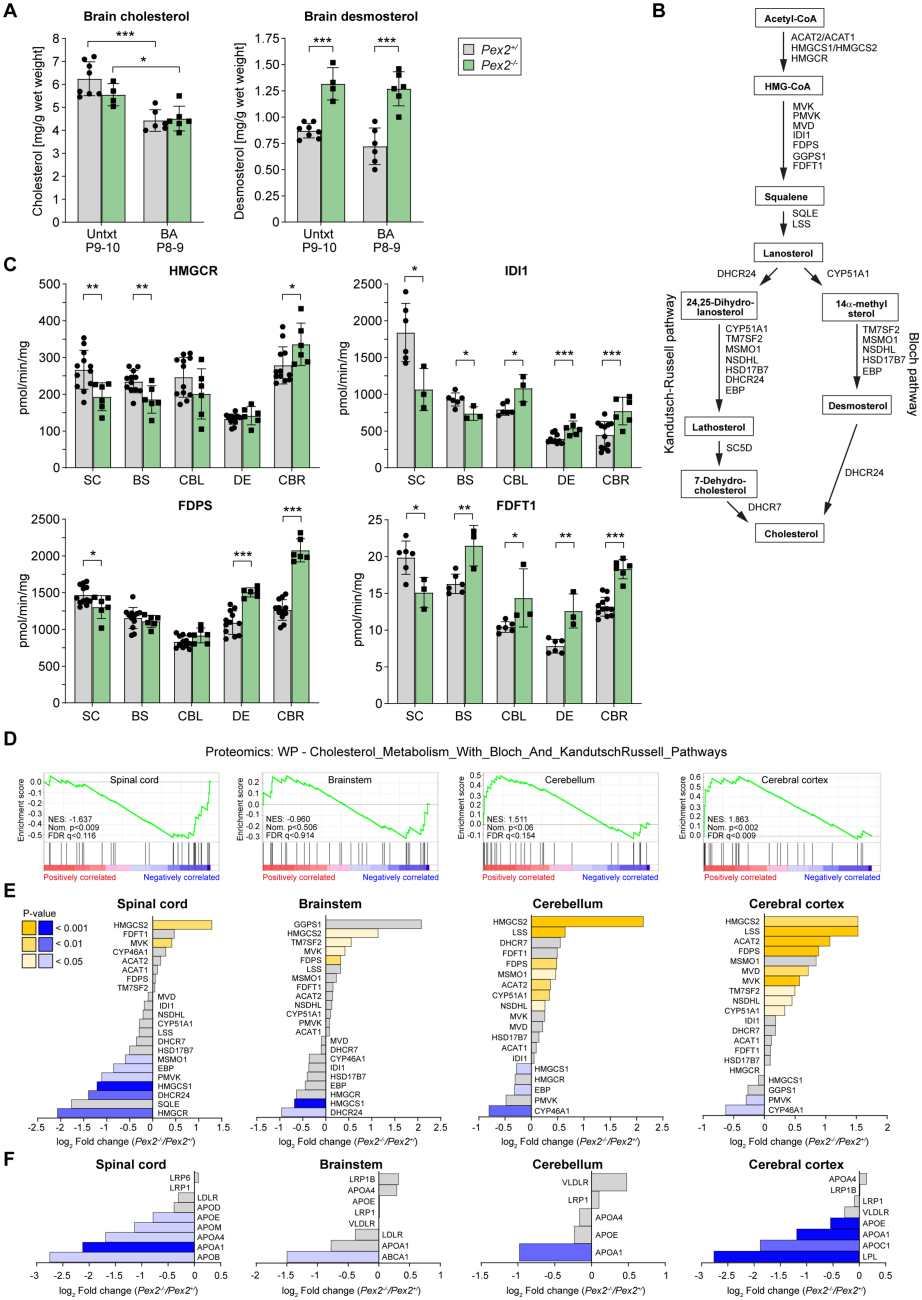
Analysis of cholesterol metabolism in the CNS. **(A)** Concentration of cholesterol and desmosterol in the brain of untreated and BA-fed P8-10 control and *Pex2*^−/−^ mice. Data are mean ± SD (*n* = 8 for control mice; *n* = 4 for *Pex2*^−/−^ mice). **(B)** Schematic representation of the cholesterol biosynthetic pathway. **(C)** Activities of cholesterol biosynthetic enzymes in different regions of the CNS of P10 control and *Pex2*^−/−^ mice. **(D)** GSEA of proteomics data revealed a negative enrichment of cholesterol metabolism in the SC and BS and a positive enrichment in the CBL and CBR. NES, normalized enrichment score; FDR, false discovery rate (*q* value); Nom., nominal. **(E,F)** Levels of proteins involved in **(E)** cholesterol biosynthesis and turnover and **(F)** cholesterol transport in SC, BS, CBL, and CBR of P10 control and *Pex2*^−/−^ mice. Blue bars: significantly downregulated proteins; yellow bars: significantly upregulated proteins; gray bars: no significant change. Data are mean ± SD (*n* = 6–12 for control mice; *n* = 3–6 for *Pex2*^−/−^ mice). Statistical analysis was performed using Student’s *t*-test or Student’s *t*-test with Welch’s correction **(C)** or two-way ANOVA followed by Tukey’s multiple comparisons test **(A)**. ^*^p < 0.05, ^**^p < 0.01, and ^***^p < 0.001; versus control mice.

We previously showed that the rate of cholesterol synthesis in the liver of P9 *Pex2*^−/−^ mice was 13-fold higher than in controls, and bile acid (BA) feeding reduced hepatic cholesterol synthesis in *Pex2*^−/−^ mice to control levels (Kovacs et al., 2004, 2009). Interestingly, the rate of cholesterol synthesis in the brain of P9 *Pex2*^−/−^ mice was significantly decreased ~2-fold compared to controls, and BA feeding increased cholesterol synthesis in *Pex2*^−/−^ brain to control levels (Kovacs et al., 2004, 2009). Cholesterol and desmosterol were the only sterols detected in significant amounts in the brains of P8-10 control and *Pex2*^−/−^ mice. While cholesterol levels were similar in the brains of control and *Pex2*^−/−^ mice, desmosterol levels were significantly elevated in the brains of both untreated and BA-fed *Pex2*^−/−^ mice compared to their control littermates (Figure 3A). While BA feeding had no effect on desmosterol levels in control and *Pex2*^−/−^ mice compared to untreated mice, total cholesterol levels decreased (Figure 3A).

### Cholesterol biosynthetic enzyme activities and protein levels in the CNS of *Pex2*^−/−^ mice

Since the brain depends on intracerebral *de novo* synthesis of cholesterol, the rate of cholesterol synthesis is highest during the early phase of postnatal brain development. As SW/129 *Pex2*^−/−^ can survive for about 1–2 weeks, we investigated the effects of peroxisome deficiency on the activities of cholesterol biosynthetic enzymes in the CNS of P10 control and *Pex2*^−/−^ mice. We determined the activities of HMGCR, IDI1, FDPS and FDFT1 as a measure of cholesterol perturbation (Figures 3B,C). All enzyme activities were significantly increased between 20 and 72% in the cerebral cortex of *Pex2*^−/−^ mice. Except for HMGCR the activities were also significantly increased by 40–60% in the diencephalon of *Pex2*^−/−^ mice. In contrast, the activities of all four enzymes were significantly decreased by ~11–42% in the spinal cord of *Pex2*^−/−^ mice. HMGCR and IDI1 activities were also significantly decreased by ~21% in the brainstem of the knockout mice, whereas FDFT1 was significantly increased by 32% and FDPS was unaltered. The activities of IDI1, FDPS and FDFT1 were slightly increased in the cerebellum of *Pex2*^−/−^ mice, while the activity of HMGCR was decreased, although not significantly.

**Figure 4:**
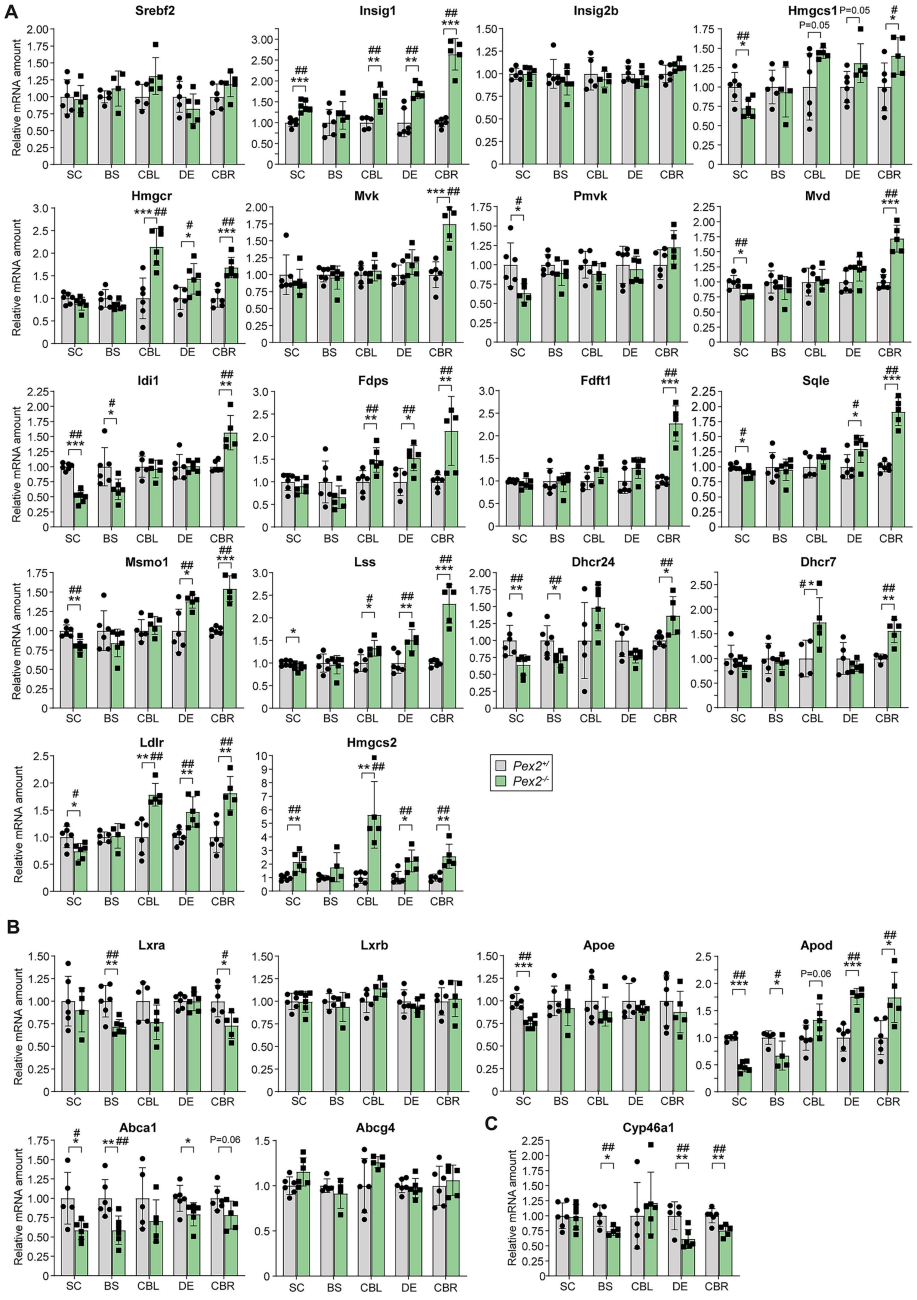
Expression of genes involved in **(A)** cholesterol biosynthesis and its regulation, **(B)** cholesterol efflux and transport, and **(C)** cholesterol turnover in the CNS of P10 control and *Pex2*^−/−^ mice. Data are mean ± SD. Statistical analysis was performed using Student’s *t*-test or Student’s *t*-test with Welch’s correction, followed by a multiple comparison correction using the Benjamini–Hochberg procedure. ^*^p < 0.05, ^**^p < 0.01, and ^***^p < 0.001; ^#^adjusted *p* < 0.1 and ^##^adjusted *p* < 0.05; versus the corresponding CNS region in control mice.

GSEA of the proteomics data showed that the SC of *Pex2*^−/−^ mice had a significant negative enrichment score for “Cholesterol metabolism with Bloch and Kandutsch–Russell pathways”, whereas the CBR had a significant positive enrichment score (Figures 3B,D and Supplementary Table S5). There was a trend toward negative and positive enrichment scores in the BS and CBL, respectively (Figure 3D). In addition, GSEA using gene sets from the GOBP database revealed significant negative enrichment scores for “Sterol biosynthetic process”, “Steroid metabolic process”, “Steroid biosynthetic process”, and “Sterol homeostasis” in the SC of *Pex2*^−/−^ mice, whereas the CBR has a positive enrichment score for “Isoprenoid biosynthetic process” (Supplementary Figure S4A and Supplementary Table S5). The protein levels of most cholesterol biosynthetic enzymes were decreased in the SC of *Pex2*^−/−^ mice (Figure 3E). In particular, the rate-limiting enzyme HMGCR was strongly downregulated in the SC. HMGCR levels were also decreased in the BS and CBL, though this decrease was not significant. These data are consistent with the observed HMGCR activities in these CNS regions (Figure 3C). Despite an upregulation of activity in the *Pex2*^−/−^ CBR, HMGCR protein levels remained unchanged, suggesting that posttranscriptional modifications contribute to the increased activity (Figures 3C,E). The protein levels of 24-dehydrocholesterol reductase (DHCR24), which catalyzes the last step in the Bloch pathway, the conversion of desmosterol to cholesterol, were significantly decreased in the SC and BS. In CBR and CBL, DHCR24 could not be detected, but only 7-dehydrocholesterol reductase (DHCR7), which catalyzes the last step of the Kandutsch–Russell pathway. Consistent with the increased enzyme activities of HMGCR, IDI1, FDPS and FDFT1 in the CBR of *Pex2*^−/−^ mice, the protein levels of most cholesterol biosynthetic enzymes were also significantly increased in the CBR (Figure 3E). The lipogenic pathways responsible for cholesterol synthesis are the same in oligodendrocytes as in other cells, except that oligodendrocytes preferentially use ketone bodies rather than glucose as a substrate for cholesterol synthesis (Koper et al., 1981). In fact, mRNA and protein levels of the mitochondrial enzyme 3-hydroxy-3-methylglutaryl-CoA synthase 2 (HMGCS2), which is involved in ketone body synthesis (Hegardt, 1999), were significantly increased in the CNS of *Pex2*^−/−^ mice (Figures 3E, 4A).

CYP46A1, which converts cholesterol to 24(S)-hydroxycholesterol for excretion from the CNS to the systemic circulation, is expressed exclusively in neurons (Dietschy and Turley, 2004). The protein levels of CYP46A1 were decreased in the CBR, CBL, and BS of *Pex2*^−/−^ mice, and the mRNA expression of *Cyp46a1* was significantly decreased in the CBR, DE, and BS (Figures 3E, 4C).

While plasma lipoproteins do not cross the blood–brain-barrier, many of the proteins involved in transporting cholesterol in the circulation are also present in the CNS, suggesting that these proteins are involved in cholesterol transport among cells of the brain (Björkhem and Meaney, 2004; Dietschy and Turley, 2004; Vance et al., 2005). The brain contains high-density lipoprotein-like transport vehicles for lipids (Vitali et al., 2014), and astrocytes in particular distribute lipids via lipoproteins (Wahrle et al., 2004; Karten et al., 2006; Kim et al., 2008; Mahley, 2016). The protein levels of apolipoprotein E (ApoE), which is the predominant apolipoprotein in the CNS and mainly expressed by astrocytes and microglia, were significantly decreased in the SC and CBR of *Pex2*^−/−^ mice (Figure 3F). However, levels of other lipoproteins such as ApoA1, ApoA4, ApoB, ApoC1, and ApoM, were also decreased in *Pex2*^−/−^ mice (Figure 3F).

In conclusion, the activities and protein levels of cholesterol biosynthetic enzymes in the SC, where white matter is more abundant, were decreased in *Pex2*^−/−^ mice, whereas they were significantly increased in the cerebral cortex, which is enriched for gray matter. Furthermore, decreased protein levels of several apolipoproteins suggest that intercellular cholesterol and lipid transport is negatively affected in the CNS of *Pex2*^−/−^ mice.

### Expression of cholesterol metabolic genes in the CNS of *Pex2*^−/−^ mice

We previously reported that the expression of SREBP-2-regulated cholesterol biosynthesis genes was significantly increased in the liver and extrahepatic tissues of *Pex2*^−/−^ mice at P10 (Kovacs et al., 2004, 2009; Charles et al., 2020). SREBP-2 regulates the expression of all cholesterol biosynthesis genes, however, the individual genes are regulated to varying degrees (Horton et al., 2002). Next, we examined the expression of *Srebf2* and its target genes in the CNS of P10 control and *Pex2*^−/−^ mice to further characterize the cholesterol biosynthetic pathway (Figure 4A). While the expression of *Srebf2* and *Insig2b* was comparable in all CNS regions of control and *Pex2*^−/−^ mice, the expression of *Insig1* was significantly increased in *Pex2*^−/−^ mice. *Insig1* is an SREBP-2 target gene and blocks the ER-to-Golgi trafficking of SCAP (SREBP cleavage-activating protein)/SREBPs (Brown et al., 2018). The expression of all cholesterol biosynthesis genes and *Ldlr* (low-density lipoprotein receptor) was significantly upregulated in the CBR of *Pex2*^−/−^ mice (Figure 4A). *Hmgcr*, *Fdps*, *Lss* (lanosterol synthase), and *Ldlr* mRNA levels were significantly increased in both CBL and DE, whereas *Sqle* (squalene epoxidase) and *Msmo1* (methylsterol monooxygenase 1) were significantly upregulated in DE (Figure 4A). The expression of *Hmgcs1*, *Pmvk* (phosphomevalonate kinase), *Mvd* (mevalonate diphosphate decarboxylase), *Idi1*, *Sqle*, *Msmo1*, *Lss*, *Dhcr24*, and *Ldlr* was significantly reduced in the SC of *Pex2*^−/−^ mice (Figure 4A). In contrast to genes involved in cholesterol biosynthesis, *Hmgcs2* expression was significantly increased in SC, CBL, DE and CBR, while showing a trend toward increased expression in BS (Figure 4A). In general, it can be said that altered enzyme activities and protein levels are reflected in altered transcription of these enzymes.

Next, we examined the spinal cord and cerebral cortex of newborn (P0) control and *Pex2*^−/−^ mice to see if the expression of cholesterol biosynthetic genes was already altered (Supplementary Figure S4B). In the spinal cord, only the expression of *Dhcr7* was significantly reduced, whereas in the cerebral cortex the expression of *Insig1*, *Hmgcr*, *Mvd* and *Dhcr7* was significantly increased.

Cells release lipids through the action of ATP-binding cassette (ABC) transporters, of which ABCA1 and ABCG1 are the most abundant ones in astrocytes, whereas ABCG4 mediates sterol efflux in neurons (Kim et al., 2008; Chen et al., 2013; Wang and Eckel, 2014). The nuclear hormone receptor liver X-activated receptors alpha (LXRα) and beta (LXRβ) are activated by certain oxysterols and desmosterol and positively regulate the expression of *Abca1*, *Abcg1*, *Abcg4*, *ApoE*, and *ApoD* (Wang et al., 2002, 2008; Whitney et al., 2002; Yang et al., 2006; Spann et al., 2012; Courtney and Landreth, 2016; Berghoff et al., 2022). Whereas *Lxrb* expression was similar in control and *Pex2*^−/−^ mice, *Lxra* expression was decreased in the BS, CBL, and CBR of *Pex2*^−/−^ mice (Figure 4B). *Abca1* expression was significantly decreased in the SC, BS, and DE and tended to be decreased in CBL and CBR (Figure 4B). ABCA1 protein levels were also significantly decreased in the BS of *Pex2*^−/−^ mice but were not detected in the other tissues (Figure 3F). *Abcg1* expression was very low and could not be reliably determined, and *Abcg4* expression was similar in control and *Pex2*^−/−^ mice. While ApoE protein levels were decreased in the SC, CBL, and CBR (Figure 3F), *Apoe* expression was significantly decreased only in the SC (Figure 4B). The expression of *Apod* was significantly decreased in the SC and BS, but increased in the CBL, DE, and CBR (Figure 4B). Interestingly, an increased expression of *Apod* was observed in cultured astrocytes and in the brain of *Npc1* knockout mice (Suresh et al., 1998), a model for the inherited lysosomal cholesterol storage disorder Niemann-Pick disease type C, which is associated with defects in cellular cholesterol homeostasis. The levels of ApoD were also increased in the hippocampus and cerebrospinal fluid of Alzheimer’s patients (Rassart et al., 2000).

### Expression of fatty acid metabolic genes and proteins in the CNS of *Pex2*^−/−^ mice

Fatty acid synthesis and myelination in the CNS are intimately connected, as myelination heavily relies on the synthesis of specific lipids, including fatty acids, to build the myelin sheath around neurons (Montani, 2021). It has been shown that endogenous fatty acid synthesis in oligodendrocytes (OLs) and Schwann cells is required for CNS and peripheral nervous system myelination, respectively (Montani et al., 2018; Dimas et al., 2019). Previous studies have shown that VLCFAs accumulate in the brain of *Pex2*^−/−^ mice at postnatal days 5, 11 and 13, as both the C26:0/C22:0 ratio and C26:0% were significantly increased (Faust et al., 2001). The increased levels of VLCFAs can be easily explained by the fact that in *Pex2*^−/−^ mice, the proteins of peroxisomal fatty acid β-oxidation are among the most reduced proteins (Figure 1E). In the CNS, the primary fatty acids involved in myelination are long-chain fatty acids, such as docosahexaenoic acid (C22:6n3; DHA) and arachidonic acid (C20:4n6), which are critical components of the myelin membrane. DHA is also considered to be essential for normal neurologic development, especially in the brain and retina, and a deficiency in brain DHA is associated with abnormal brain development in humans with ZSD (Faust et al., 2001). The synthesis of DHA requires one cycle of peroxisomal β-oxidation (Wanders et al., 2023), and accordingly it has been shown that the DHA content in the brain of *Pex2*^−/−^ mice is reduced compared to controls at birth and at 5, 11 and 13 days after birth (Faust et al., 2001).

**Figure 5:**
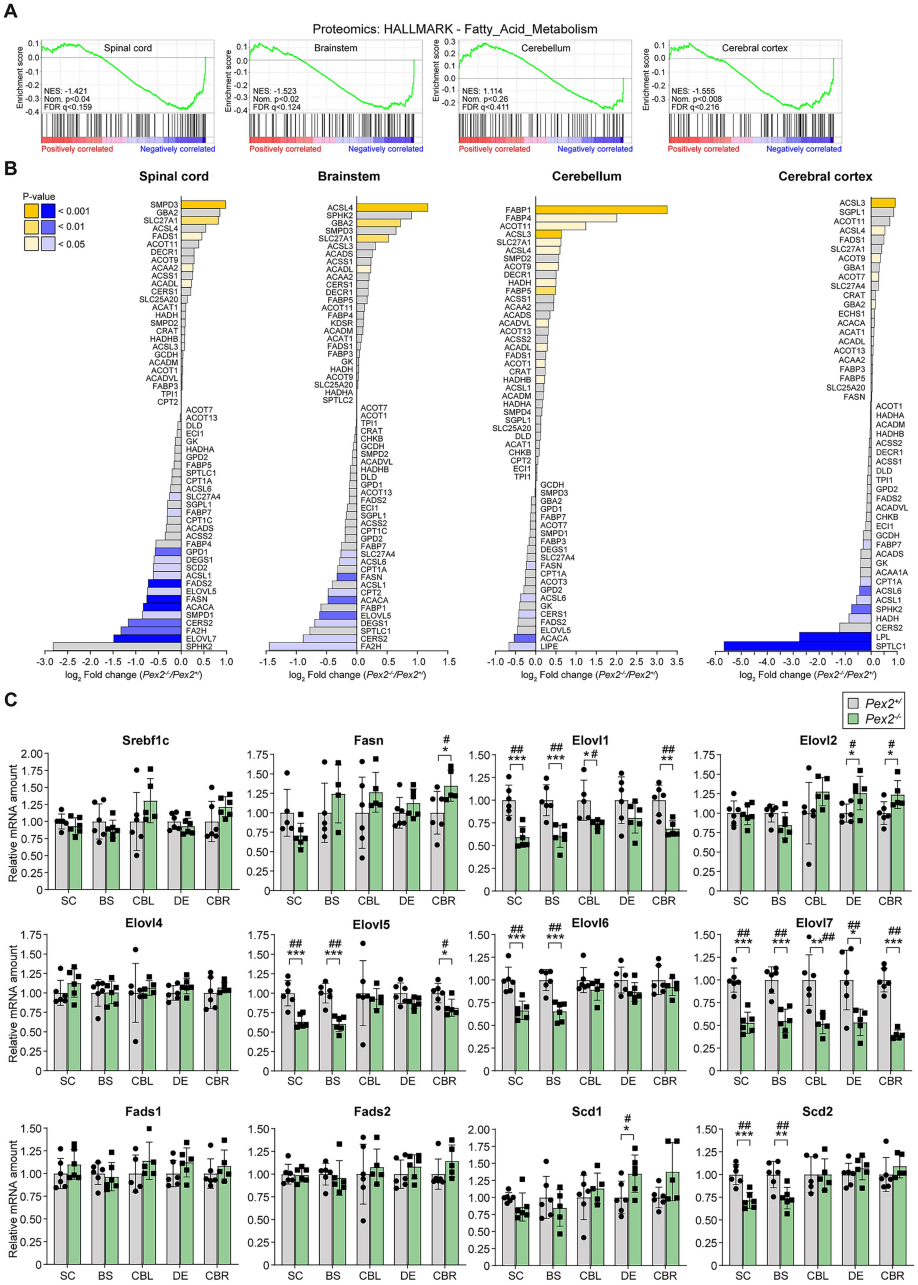
Analysis of fatty acid metabolism in the CNS. **(A)** GSEA of proteomics data revealed downregulation of fatty acid metabolism in the CNS of P10 *Pex2*^−/−^ mice. **(B)** Levels of proteins involved in fatty acid and sphingolipid metabolism in SC, BS, CBL, and CBR of P10 control and *Pex2*^−/−^ mice. Blue bars: significantly downregulated proteins; yellow bars: significantly upregulated proteins; gray bars: no significant change. **(C)** Expression of genes involved in fatty acid biosynthesis in the CNS of P10 control and *Pex2*^−/−^ mice. Data are mean ± SD. Statistical analysis was performed using Student’s *t*-test or Student’s *t*-test with Welch’s correction, followed by a multiple comparison correction using the Benjamini–Hochberg procedure. ^*^p < 0.05, ^**^p < 0.01, and ^***^p < 0.001; ^#^adjusted *p* < 0.1 and ^##^adjusted *p* < 0.05; versus the corresponding CNS region in control mice.

GSEA of proteomics data clearly showed a negative enrichment of “fatty acid metabolism” in the SC, BS, and CBR of *Pex2*^−/−^ mice (Figure 5A and Supplementary Table S5), and the downregulation of proteins under this term suggests impaired fatty acid activation, degradation, and synthesis. SLC27A1 (FATP1) protein levels were significantly increased in the SC, BS, and CBL of *Pex2*^−/−^ mice (Figure 5B). It mediates the import of long-chain fatty acids (LCFAs) into the cell by facilitating their transport at the plasma membrane and also functions as an acyl-CoA ligase, catalyzing the ATP-dependent formation of fatty acyl-CoA using LCFAs and VLCFAs as substrates, which prevents fatty acid efflux from cells and might drive more fatty acid uptake (Hirsch et al., 1998; Watkins, 2008; Murphy, 2017). The fatty acid-binding proteins FABP1, FABP4, and FABP5 were significantly increased in the CBL of *Pex2*^−/−^ mice (Figure 5B). FABP4, typically found in adipocytes, has been shown to be significantly overexpressed in cerebellar liponeurocytoma, a very rare CNS tumor, compared to normal adult cerebellum (Anghileri et al., 2012). In the infantile monkey cerebellum, both granule cell progenitors in the external granular layer and oligodendrocyte progenitors in the internal granular layer express FABP5 (Boneva et al., 2010). Protein levels of acyl-CoA synthetase long-chain family member 4 (ACSL4) were also increased in all CNS regions examined (Figure 5B). ACSL4 catalyzes the conversion of long-chain fatty acids to their active form acyl-CoA for both cellular lipid synthesis and degradation via β-oxidation. ACSL4 preferentially activates arachidonate and eicosapentaenoate as substrates and is therefore a likely regulator of lipid mediator production (Golej et al., 2011). Proteins of the mitochondrial beta-oxidation of fatty acids (e.g., ACADS, ACADM, ACADL, HADH, HADHA, HADHB, ACAA2, ECHS1) were generally not significantly altered or moderately upregulated (Figure 5B).

ACACA and FASN, which catalyze the first steps in fatty acid synthesis, were significantly decreased in the SC and BS (Figure 5B). Particularly in SC, proteins involved in fatty acid metabolism were significantly downregulated. Elongases and desaturases play a critical role in regulating the length and degree of unsaturation of fatty acids in mammalian cells (Supplementary Figure S5). Elongation of very long-chain fatty acids 5 (ELOVL5) was decreased in the SC, BS, and CBL, and ELOVL7 and fatty acid desaturase 2 were significantly downregulated in SC (Figure 5B). Another protein that was strongly reduced in SC and BS was fatty acid 2-hydroxylase (FA2H) (Figure 5B). FA2H catalyzes the hydroxylation of free fatty acids at the C-2 position to produce 2-hydroxy fatty acids, which are building blocks of sphingolipids and glycosphingolipids found in neural tissue and major constituents of myelin (Eckhardt, 2023). The levels of other proteins involved in sphingolipid metabolism were also significantly altered (Figure 5B). Levels of ceramide synthase 2 (CERS2) and dihydroceramide desaturase 1 (DEGS1) were significantly decreased in the SC and BS, while CERS1 levels decreased in the CBL (Figure 5B). Serine palmitoyl transferase 1 (SPTLC1) and sphingosine kinase 2 (SPHK2) levels were significantly decreased in the CBR (Figure 5B). In contrast, levels of sphingomyelin phosphodiesterase 3 (SMPD3), which catalyzes the hydrolysis of sphingomyelins, and glucosylceramidase beta 2 (GBA2), which catalyzes the hydrolysis of glucosylceramides, were significantly increased in the SC and BS (Figure 5B).

Next, we determined the mRNA expression of genes involved in fatty acid synthesis and their regulation (Figure 5C). The activities of the enzymes involved in the elongation and desaturation of fatty acids are primarily regulated at the transcriptional level rather than by post-translational modifications (Guillou et al., 2010). While the expression of *Srebf1c* and fatty acid synthase (*Fasn*) was comparable in all CNS regions of control and *Pex2*^−/−^ mice, the expression of Elovl7 was significantly decreased in *Pex2*^−/−^ mice (Figure 5C). In addition, the mRNA levels of *Elovl1*, *Elovl5*, and *Elovl6* were significantly decreased in the SC and BS of *Pex2*^−/−^ mice (Figure 5C). The expression of *Elovl1* was also significantly decreased in the CBL and CBR and tended to be decreased in the DE of *Pex2*^−/−^ mice, whereas the expression of *Elovl2* was significantly increased in the DE and CBR (Figure 5C). Interestingly, ELOVL1 has been shown to catalyze the synthesis of both saturated VLCFA (C26:0) and monounsaturated VLCFA (C26:1) in X-linked adrenoleukodystrophy (X-ALD) skin fibroblasts (Ofman et al., 2010; Schackmann et al., 2015). ELOVL1 was not detected at the protein level, but since *Elovl1* expression was significantly reduced while C26:0 levels were increased in the brain, other elongases might be responsible for the synthesis of C26:0 in the CNS of *Pex2*^−/−^ mice. *Elovl3* expression was very low and could not be reliably determined. Among the desaturases, only the expression of *Scd2* was significantly reduced in SC and BS, and the expression of *Scd1* was increased in the DE (Figure 5C).

We examined the spinal cord and cerebral cortex of newborn control and *Pex2*^−/−^ mice to see if the expression of fatty acid biosynthetic genes was already altered (Supplementary Figure S6A). In the spinal cord, only the expression of *Fasn*, *Scd1*, *Elovl7* and Fa2h was significantly reduced, whereas in the cerebral cortex the expression of genes encoding fatty acid biosynthetic enzymes was not altered.

We investigated whether the changes in the levels of proteins involved in sphingolipid metabolism were also reflected at the level of gene expression. In general, the expression of genes encoding proteins involved in sphingolipid metabolism was unchanged or only slightly altered (Supplementary Figure S6B). While CERS1 protein levels were reduced in the cerebellum, *Cers1* expression was significantly increased. In line with the altered protein levels in the SC, the mRNA levels of *Cers2*, *Degs1*, *Sphk2*, and *Sptlc1* decreased significantly, while *Smpd3* expression increased. *Smpd3* expression increased significantly in the CBL, DE, and CBR, and increased slightly in the BS. Although SPTLC1 and SPHK2 protein levels were significantly decreased in the CBR, their gene expression remained unchanged. The expression of Degs1 was significantly decreased in the BS, which is in line with the reduced protein level.

### Myelination is decreased in the CNS of *Pex2*^−/−^ mice

**Figure 6:**
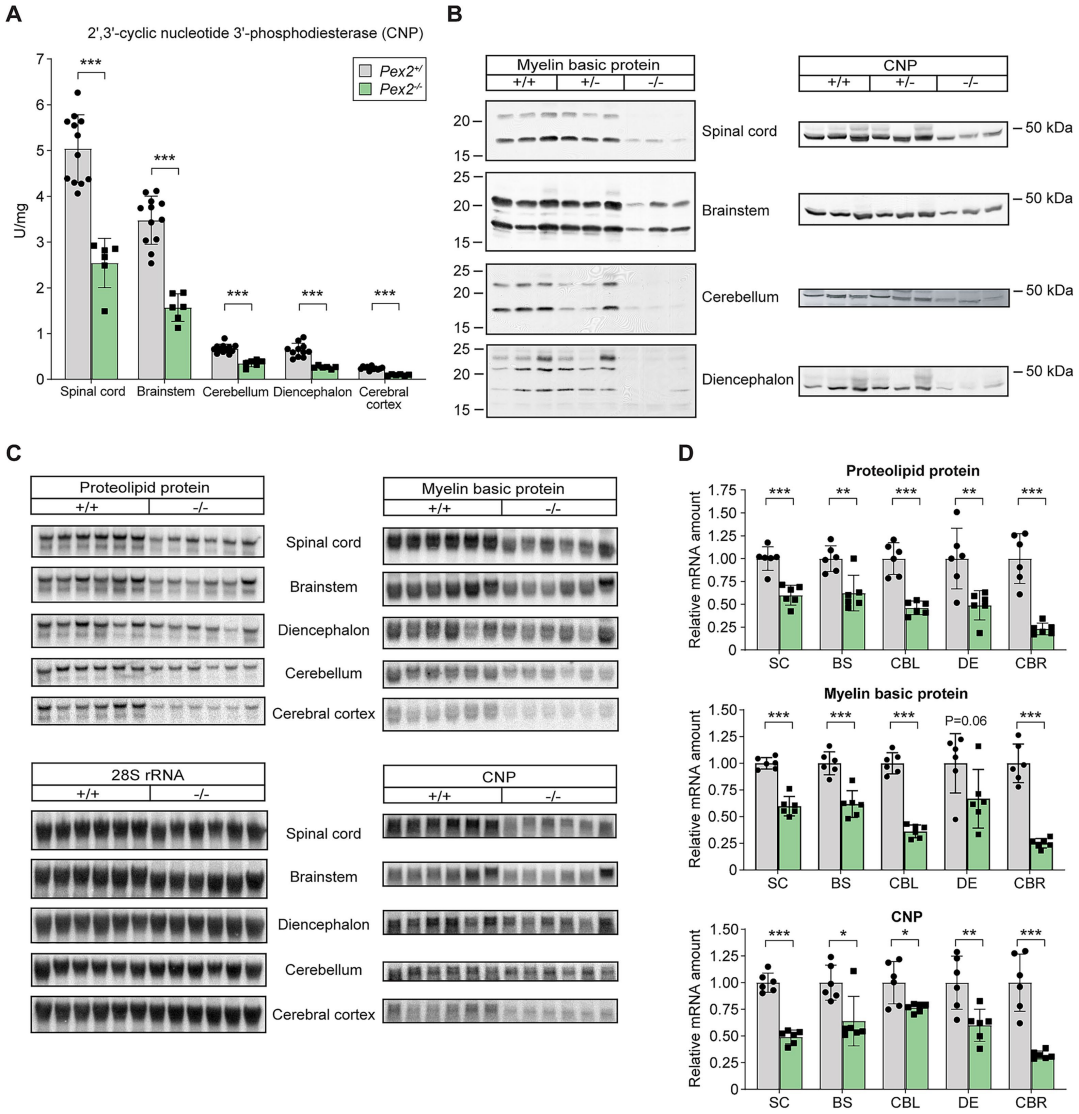
Activities, protein, and expression levels of myelin proteins in different regions of the CNS of P10 control and *Pex2*^−/−^ mice. **(A)** Activities of 2′,3′-cyclic nucleotide 3′-phosphodiesterase (CNP), a marker protein of myelin-forming glial cells, were determined in homogenates of the indicated CNS regions. Data are mean ± SD (*n* = 12 for control mice; *n* = 6 for *Pex2*^−/−^ mice). Statistical analysis was performed using Student’s *t*-test or Student’s *t*-test with Welch’s correction. ^***^p < 0.001 versus the corresponding CNS region in control mice. **(B)** Whole tissue lysates were assessed by immunoblotting for CNP and myelin basic protein. The exposure time for the immunoblots of the different CNS regions was different. **(C,D)** Expression of proteolipid protein (*Plp1*), myelin basic protein (*Mbp*), and *Cnp* mRNA in different CNS regions of P10 control and *Pex2*^−/−^ mice. **(C)** 20 g of total RNA were subjected to electrophoresis and blot hybridization with the indicated ^32^P-labeled probe. The amount of radioactivity in each band was quantified by PhosphorImaging and normalized to the signal generated by 28S ribosomal RNA. **(D)** The fold change in expression in *Pex2*^−/−^ mice was expressed relative to matching control mice, which was arbitrarily set at 1. Data are mean ± SD (*n* = 6). ^*^p < 0.05, ^**^p < 0.01, and ^***^p < 0.001 versus the corresponding CNS region in control mice.

Myelin formation in the rodent CNS occurs mainly during the first 3 weeks of postnatal development. It has been proposed that myelination proceeds in a strictly rostral-caudal direction in the CNS, with the exception of the dorsal spinal cord, where myelination starts first in the cervical enlargement and continues in both rostral and caudal directions (Foran and Peterson, 1992; Ozarkar et al., 2025). To examine the effect of peroxisome deficiency on the myelination in the CNS, we analyzed activities, expression and protein levels of myelin proteins in P10 control and *Pex2*^−/−^ mice (Figure 6). The specific activity of 2′,3′-cyclic nucleotide 3′-phosphodiesterase (CNP), a widely used marker protein of myelin-forming glial cells (Vogel and Thompson, 1988; Sprinkle, 1989; Chandross et al., 1999), in the individual parts of the CNS is a good representation for the stage of myelination, with the highest activities in the spinal cord and brain stem and the lowest in the cerebral cortex (Figure 6A). CNP activity was decreased by 50–60% in all CNS regions of P10 *Pex2*^−/−^ mice compared to controls (Figure 6A).

Immunoblot analysis of myelin proteins CNP and myelin basic protein (MBP) was performed to determine whether the decreased CNP activities are a reflection of decreased myelination in *Pex2*^−/−^ mice. The protein levels of CNP and MBP were significantly decreased in the SC, BS, CBL, and DE of P10 *Pex2*^−/−^ mice (Figure 6B). The heterogeneity of MBPs is generated by alternative splicing of a single gene. CNP and MBP were barely detectable in the cerebral cortex of P10 control mice and were undetectable in *Pex2*^−/−^ mice (data not shown).

We performed comparative Northern blot analysis to quantify the mRNA expression of myelin-synthesizing proteins by measuring the expression levels of mRNAs encoding for *Mbp*, *Cnp*, and proteolipid protein (*Plp1*) in control and *Pex2*^−/−^ mice (Figures 6C,D). The expression of *Mbp*, *Cnp*, and *Plp1* was significantly decreased in all CNS regions of P10 *Pex2*^−/−^ mice (Figures 6C,D). Thus, myelin gene expression does not reach its normal peak in peroxisome-deficient *Pex2*^−/−^ mice.

**Figure 7:**
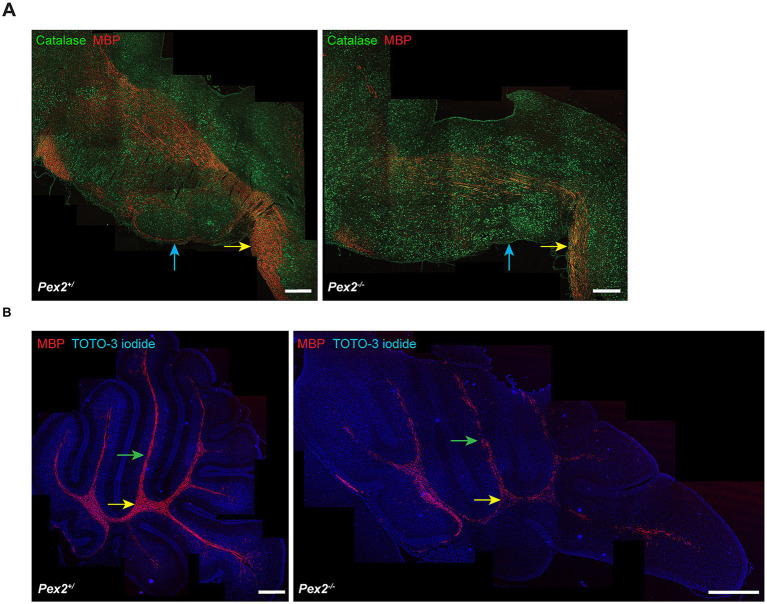
Immunohistochemical staining for myelin basic protein (MBP) shows hypomyelination in *Pex2*^−/−^ mice at P10. **(A)** Sagittal sections of spinal cord tract fibers and axonal fibers overlying the inferior olivary nucleus. Double immunofluorescence for catalase and MBP. Yellow arrows indicate spinal cord tract fibers and blue arrows indicate inferior olivary nucleus. At P10, there is a markedly reduced level of spinal cord and medullary axonal myelination in *Pex2*^−/−^ as compared to control. **(B)** Immunofluorescence for MBP demonstrates greatly attenuated myelination in both deep cerebellar white matter (yellow arrows) and folial white matter (green arrows). Cerebellar foliation abnormality is observed in *Pex2*^−/−^ mice. Nuclei were stained with TOTO-3 iodide. Image stitching was performed using ImageJ to combine multiple images to create overview images of the SC, BS, and CBL. Bars, 250 μm.

Representative immunohistochemical staining for MBP in the SC and BS (Figure 7A) and CBL (Figure 7B) demonstrated a prominently reduced level of myelination in P10 *Pex2*^−/−^ mice. Spinal cord and medullary axonal myelination were markedly reduced in *Pex2*^−/−^ mice (Figure 7A). The reduced area in which MBP is present illustrated the abnormally reduced amount of myelinated deep cerebellar white matter and folial white matter at P10. The previously described cerebellar foliation anomaly is also seen (Figure 7B) (Faust et al., 2001, 2005; Faust, 2003).

### Comparison of *Pex2*^−/−^ CNS proteins with the mouse myelin proteome

**Figure 8:**
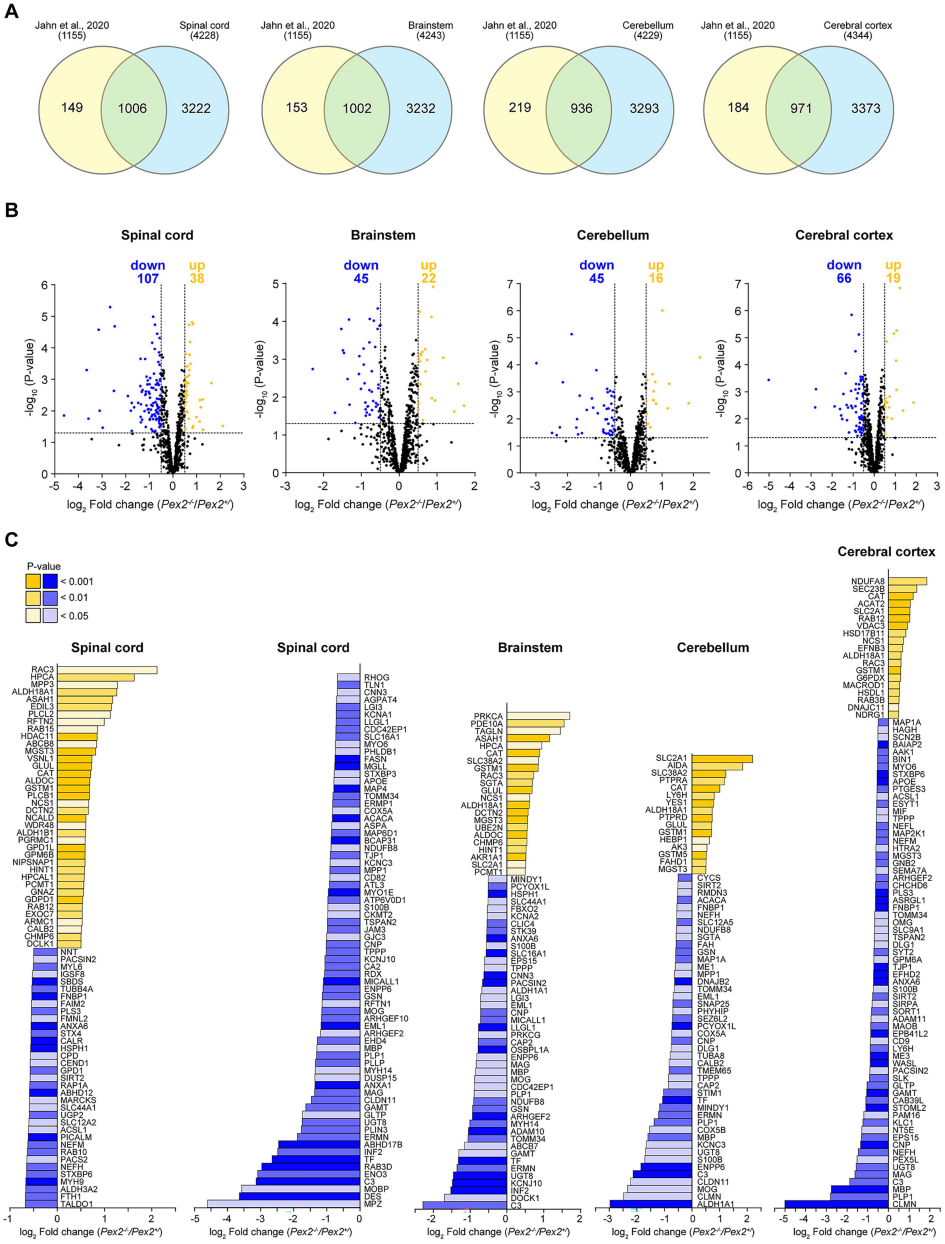
**(A)** Venn diagrams showing the overlap of proteins identified in SC, BS, CBL, and CBR of P10 control and *Pex2*^−/−^ mice (blue) with the mouse myelin proteome (yellow) reported by Jahn et al. (2020). **(B)** Volcano plots of SC, BS, CBL, and CBR proteins from control and *Pex2*^−/−^ mice overlapping with the myelin proteome reported by Jahn et al. (2020). Cut-offs for log_2_ fold change and *p*-value were set at ±0.5 and 0.05, respectively. Blue dots: significantly downregulated proteins; yellow dots: significantly upregulated proteins; black dots: no significant change. Numbers of significantly up- or downregulated proteins are indicated in the plot. **(C)** Protein levels of significantly altered myelin-associated proteins from **(B)** are shown. Blue bars: significantly downregulated proteins; yellow bars: significantly upregulated proteins; gray bars: no significant change.

Using various proteomic approaches, the proteome of biochemically purified myelin from the brains of C56Bl/6N mice was determined and 1,155 proteins were identified (Jahn et al., 2020). It was shown that PLP1, MBP, CNP, and myelin oligodendrocyte glycoprotein (MOG) represent about 73% of the total myelin protein (38, 30, 5, and 1%, respectively) (Jahn et al., 2020). However, this myelin protein dataset extends well beyond the most abundant myelin components. We showed that myelination is significantly reduced in *Pex2*^−/−^ mice at P10 using immunoblots against MBP and CNP, the second and third most abundant myelin proteins (Figure 6B). Next, we compared all proteins detected in SC, BS, CBL and CBR in our study with the mouse CNS myelin proteome determined by Jahn et al. (2020) to investigate how other myelin-related proteins are altered in *Pex2*^−/−^ mice at P10. Among the detected proteins, 1,006, 1,002, 936, and 971 proteins in SC, BS, CBL, and CBR, respectively, overlapped with the mouse myelin proteome (Figure 8A and Supplementary Table S7). We then compared all significantly altered proteins (log_2_ FC and *p*-value cut-offs were set at ±0.5 and 0.05, respectively) in SC, BS, CBL and CBR (706, 467, 361, 447, respectively) with the mouse myelin proteome. Among the significantly altered proteins, 145, 67, 61, and 85 proteins in SC, BS, CBL, and CBR, respectively, overlapped with the mouse myelin proteome (Figures 8B,C; Supplementary Figure S7A). The protein levels of the majority of the overlapping and significantly altered proteins were decreased in the SC, BS, CBL, and CBR of *Pex2*^−/−^ mice at P10 (Figures 8B,C). For example, the levels of major myelin structure proteins such as PLP1, MBP, CNP, MOG, and MAG were significantly decreased in *Pex2*^−/−^ mice (Figure 8C; Supplementary Figure S7B).

In addition, 20 of the overlapping proteins were detected in the SC, BS, and CBL, with the levels of 15 proteins being down-regulated and 5 proteins being up-regulated (Supplementary Figure S7B). The downregulated proteins PLP1, MBP, CNP, TPPP (tubulin polymerization promoting protein), ENPP6 (ectonucleotide pyrophosphatase/phosphodiesterase 6), GSN (gelsolin), MOG, and UGT8 (UDP-glycosyltransferase 8) are well-known myelin proteins (Jahn et al., 2020). TPPP acts as a microtubule nucleation factor in oligodendrocytes and promotes the elongation of the myelin sheath, and *Tppp* knockout mice have hypomyelination and motor coordination defects (Fu et al., 2019). ENPP6 has been identified as a marker of newly forming oligodendrocytes (Xiao et al., 2016), it is required for the development of the myelin sheath and *Enpp6*^−/−^ mice exhibit hypomyelination (Morita et al., 2016). UGT8 catalyzes the transfer of galactose to ceramide, a key enzymatic step in the biosynthesis of galactocerebrosides, which are abundant sphingolipids of the CNS myelin sheath. Hypomyelination has been reported in *Ugt8*^−/−^ mice (Dupree et al., 1998). The actin disassembly factor gelsolin is required for normal CNS myelin wrapping (Zuchero et al., 2015). Similarly, MBP is also essential for CNS myelin wrapping and actin disassembly (Zuchero et al., 2015). S100B (S100 calcium binding protein B) is expressed in oligodendrocytes, highly expressed in astrocytes, and one of the most abundant soluble proteins in the brain (Michetti et al., 2019, 2023). It is also used as an astrocyte activation marker. S100B was significantly downregulated in the CNS of *Pex2*^−/−^ mice at P10 (Supplementary Figure S7B). The upregulated proteins catalase (CAT), GSTM1 (glutathione S-transferase mu 1), and MGST3 (microsomal glutathione S-transferase 3) are involved in the response to oxidative stress. Increased levels of GLUL (glutamate-ammonia ligase), which regulates the levels of toxic ammonia and converts neurotoxic glutamate to harmless glutamine, may be part of the response of the *Pex2*^−/−^ CNS to inflammation or cellular stress. ALDH18A1 plays a role in glutamate metabolism and may also be involved in the response to oxidative stress in the CNS of *Pex2*^−/−^ mice.

### Expression of genes encoding myelin-associated proteins in the CNS of *Pex2*^−/−^ mice

**Figure 9:**
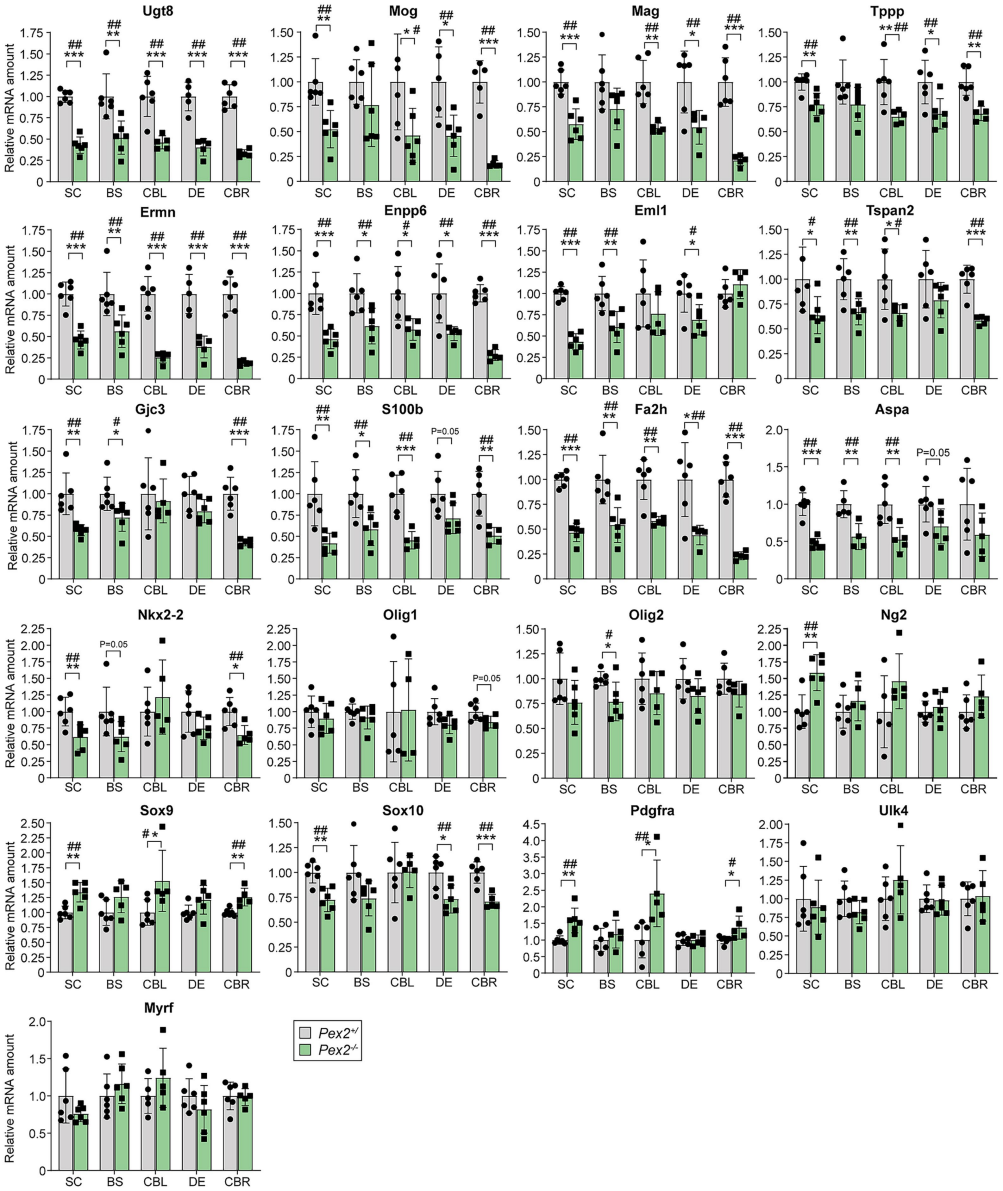
Expression of genes encoding myelin-associated proteins and proteins involved in oligodendrocyte lineage progression and differentiation in the CNS of P10 control and *Pex2*^−/−^ mice. Data are mean ± SD. Statistical analysis was performed using Student’s *t*-test or Student’s *t*-test with Welch’s correction, followed by a multiple comparison correction using the Benjamini–Hochberg procedure. ^*^p < 0.05, ^**^p < 0.01, and ^***^p < 0.001; ^#^adjusted *p* < 0.1 and ^##^adjusted *p* < 0.05; versus the corresponding CNS region in control mice.

Next, we investigated whether the changes in the protein levels of myelin-associated proteins are also reflected at the level of gene expression. In accordance with the significantly reduced protein levels of UGT8, MOG, MAG, TPPP, ENPP6 and S100B (Figure 8C), the mRNA levels of these proteins were also significantly reduced (Figure 9). Not only were the protein levels of ERMN (ermin) significantly decreased in the SC, BS, and CBL, but the gene expression was also reduced in all regions of the CNS of *Pex2*^−/−^ mice. ERMN is an actin-binding protein found almost exclusively in the CNS and expressed almost exclusively in myelinating oligodendrocytes where it plays a role in the late wrapping and/or compaction phases of myelinogenesis as well as in the maintenance and stability of the myelin sheath (Ziaei et al., 2022). The mRNA levels of *Tspan2* (tetraspanin-2) were significantly reduced in the SC, BS, CBL, and CBR and tended to be decreased in the DE (Figure 9). *Tspan2* was shown to be expressed predominantly by cells of the oligodendrocyte lineage, but *Tspan2*^−/−^ mice showed normal development and biogenesis of myelin (Yaseen et al., 2017). However, TSPAN2 and PLP1 may play a role in regulating neuroinflammation, since they were shown to be involved in suppressing astrocyte and microglia activity (Zeis et al., 2016). The protein levels of EML1 (echinoderm microtubule-associated protein-like 1), a microtubule-associated protein, were significantly decreased in the SC, BS, and CBL, and its expression was also downregulated in the SC, BS, CBL, and DE (Figure 9). EML1 indirectly affects myelination by playing a role in axon guidance and neuronal migration (Collins et al., 2019; Zhang et al., 2024).

The expression of aspartoacylase (*Aspa*), the enzyme responsible for hydrolyzing N-acetylaspartate (NAA) into acetate and aspartate, was significantly decreased in the SC, BS, and CBL and tended to be decreased in the DE and CBR (Figure 9). Protein levels of ASPA were decreased by ~45 and 30% in the SC and BS, respectively, while ASPA was not detected in the CBL and CBR (Figure 8C). NAA is highly concentrated in the brain and numerous studies have implicated that acetate produced from the hydrolysis of NAA is utilized in the synthesis of lipids and acetylation of nucleosomal histones (Mattan et al., 2010; Appu et al., 2017). Accordingly, it has been shown that *Aspa* gene expression peaks during myelination, and that a loss of ASPA activity leads to defective myelin synthesis (Namboodiri et al., 2006; Mattan et al., 2010; Moffett et al., 2011).

In the SC, BS, and CBR of *Pex2*^−/−^ mice, the mRNA levels of *Gjc3* (gap junction protein gamma 3), which is highly expressed in myelinating glial cells of the CNS and peripheral nervous system (Altevogt et al., 2002), were significantly decreased (Figure 9). Sphingolipids containing 2-hydroxylated fatty acids are among the most abundant lipid components of the myelin sheath, and both protein and mRNA levels of *Fa2h* were decreased in *Pex2*^−/−^ mice (Figures 5B, 9). *Fa2h* expression was already reduced in the SC of newborn *Pex2*^−/−^ mice, whereas expression in the P0 cerebral cortex was not altered (Supplementary Figure S6A). Unc-51 Like Kinase 4 (ULK4) is a crucial regulator of myelination and it has been shown that in *Ulk4* hypomorphic mice, myelination is reduced by ~50% and the expression of myelination-related genes, major oligodendrogeneic transcription factors, and stage-specific oligodendrocyte factors is significantly decreased (Liu et al., 2018). Interestingly, *Ulk4* was similarly expressed in control and *Pex2*^−/−^ mice (Figure 9).

Oligodendrocyte (OL) lineage progression and terminal differentiation into mature oligodendrocytes are under tight transcriptional and post-transcriptional control (Elbaz and Popko, 2019; Montani, 2021). The OL development can be divided into the following four stages: oligodendrocyte progenitor cells (OPCs), late OPCs, premyelinating OLs, and myelinating OLs. OPCs proliferate and migrate to their final destination where they differentiate into mature OLs and myelinate axons. All OPCs can be identified by the expression of the proteoglycan *Ng2* (*Cspg4*), the platelet-derived growth factor receptor alpha (*Pdgfra*), and the transcription factors *Olig1/2* and *Sox10*. These transcription factors are expressed along the entire oligodendrocyte lineage, including mature OLs. NKX2-2 is an important determinant of oligodendrocyte differentiation, and its ablation does not affect OPCs but leads to loss of OLs (Qi et al., 2001). However, the expression of these markers showed a mixed picture. The expression of *Sox10* was decreased by ~25% in the SC, DE, and CBR of *Pex2*^−/−^ mice, whereas *Nkx2-2* was significantly decreased in the SC and CBR (Figure 9). The mRNA levels of *Olig1* and *Olig2* were similar in control and *Pex2*^−/−^ mice, whereas the expression of *Sox9* and *Pdgfra* was significantly increased in the SC, CBL, and CBR of *Pex2*^−/−^ mice. Sox9 is another transcription factor that controls gliogenesis and its ablation results in the loss of OPCs (Elbaz and Popko, 2019). *Ng2* expression was significantly increased in the SC of *Pex2*^−/−^ mice and similar in other CNS regions of control and *Pex2*^−/−^ mice. The differentiation of OPCs into oligodendrocytes and regulation of myelin genes depends on the transcription factor MYRF, and its ablation results in the loss of OLs (Emery et al., 2009; Koenning et al., 2012; Elbaz and Popko, 2019). However, the expression of *Myrf* was similar in control and *Pex2*^−/−^ mice. In conclusion, while changes in the expression of markers of lineage progression and terminal differentiation of OL are observed, a dramatic reduction in gene expression that would cause the absence of these proteins and thus prevent the formation of mature OL is not evident.

### AMPK, AKT/mTOR and MAPK signaling in the CNS of *Pex2*^−/−^ mice

**Figure 10:**
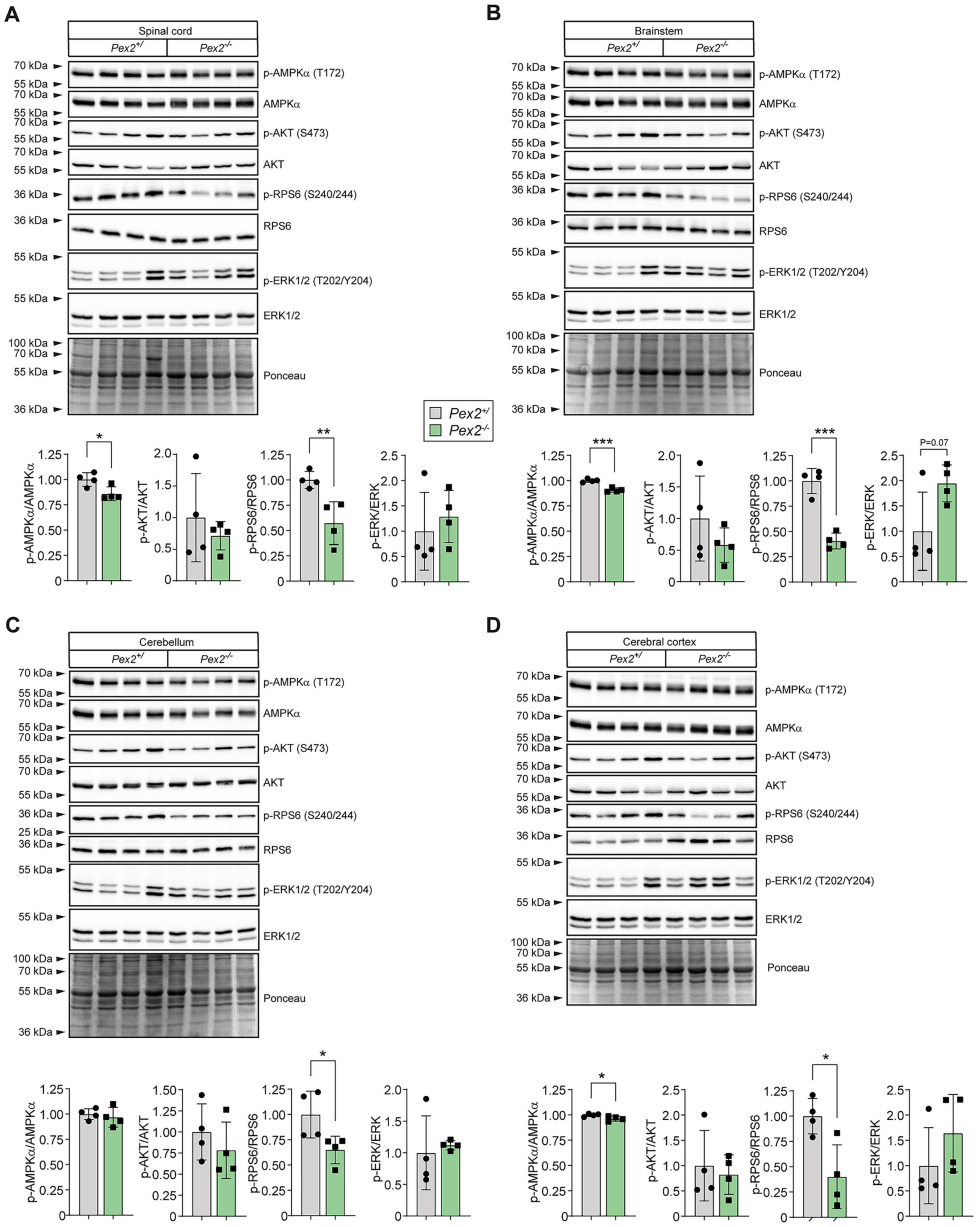
Representative Western blots and quantification of AMPK, AKT, RPS6, and MAPK signaling in the SC **(A)**, BS **(B)**, CBL **(C)**, and CBR **(D)** of P10 *Pex2*^−/−^ mice. mTOR activity was measured by phosphorylation of the downstream target RPS6. For quantification, p-AMPK, p-AKT, p-RPS6, and p-ERK were normalized to the corresponding total AMPK, AKT, RPS6, and ERK, respectively. Protein ratios are expressed relative to that in control mice, which were arbitrarily defined as 1. Data are mean ± SD. Statistical analysis was performed using Student’s *t*-test or Student’s *t*-test with Welch’s correction. ^*^p < 0.05, ^**^p < 0.01, and ^***^p < 0.001; versus control mice.

Next, we investigated whether altered AMPK, AKT/mTOR and MAPK signaling is associated with the hypomyelination phenotype in P10 *Pex2*^−/−^ mice. Adenosine monophosphate-activated protein kinase (AMPK) is a metabolic sensor that is activated by falling energy levels to restore energy balance (Steinberg and Hardie, 2023). Studies have shown that proper AMPK signaling is crucial for normal brain development and myelination (Dasgupta and Milbrandt, 2009). Liver kinase B1 activates AMPKα at Thr172 in response to ATP depletion. The SC, BS and CBR of *Pex2*^−/−^ mice showed a moderate, but significant, reduction in phosphorylation of AMPKα (Figure 10). Activated AMPK phosphorylates HMGCR at Ser 872 and decreases its activity under conditions of energy stress (Burg and Espenshade, 2011). Since AMPK was not activated, we also did not detect increased phosphorylation of HMGCR in the SC, which could contribute to its reduced activity (Supplementary Figure S4C).

It has been shown that signal transduction through the AKT and mTOR (mechanistic Target Of Rapamycin) pathway regulates myelin biogenesis and myelin maintenance in the CNS and PNS (Bercury et al., 2014; Lebrun-Julien et al., 2014; Norrmén et al., 2014; Wahl et al., 2014; Bercury and Macklin, 2015; Khandker et al., 2022). Knocking out *Pten* (phosphatase and tensin homologue), an upstream inhibitor of the phosphatidylinositol 3-kinase (PI3K)/AKT pathway, in oligodendrocytes leads to significant hypermyelination throughout the CNS, without affecting OPC differentiation (Gaesser and Fyffe-Maricich, 2016). Myelination mainly depends on mTORC1 (mTOR complex 1) function, with minor contributions from mTORC2. Studies using rapamycin, an inhibitor of mTORC1 and partial inhibitor of mTORC2, suggested that mTOR regulates oligodendrocyte differentiation (Figlia et al., 2018). It has also been shown that ribosomal protein S6 (RPS6) activation, which is a main target of mTORC1, in differentiating oligodendrocytes correlates with the peak of myelination in the brain and spinal cord (Benardais et al., 2022). mTOR also acts as a key regulator of cholesterol biosynthesis (Khandker et al., 2022). mTORC1 has been shown to modulate the activity of SREBPs in mammalian cells through various mechanisms, including p70 ribosomal S6 kinase (p70 S6K) (Peterson et al., 2011; Wang et al., 2011; Eid et al., 2017). p70 S6K is the only kinase known to be directly activated by mTORC1, which then activates RPS6 (Laplante and Sabatini, 2012). We assessed mTORC1 activity by analyzing the phosphorylation of RPS6 in lysates from the SC, BS, CBL, and CBR of P10 control and *Pex2*^−/−^ mice. Phosphorylation of RPS6 at Ser240/244 was significantly decreased in all CNS regions of *Pex2*^−/−^ mice (Figure 10), suggesting that mTORC1 activity is lower in the peroxisome-deficient CNS.

The mTOR pathway is linked with AKT signaling, both upstream of mTORC1 and downstream of mTORC2 (Laplante and Sabatini, 2012). The PI3K/AKT pathway is also involved in the transport of SCAP/SREBP-2 from the endoplasmic reticulum to the Golgi, thereby contributing to the control of SREBP-2 activation (Du et al., 2006). Unexpectedly, AKT phosphorylation at Ser473 was similar in control and *Pex2*^−/−^ mice (Figure 10).

The extracellular signal-regulated kinases (ERK1 and ERK2) are prototypical members of the mitogen-activated protein (MAP) kinase family, which links gene expression to extracellular stimuli (Gaesser and Fyffe-Maricich, 2016). *In vitro* studies have shown that the mature form of SREBP-2 is a substrate of ERK–MAPK, which affects its trans-activity (Kotzka et al., 2000, 2004). ERK signaling has also been associated with various aspects of oligodendrocyte development, including proliferation, migration, survival, differentiation and myelination (Gaesser and Fyffe-Maricich, 2016). There is also a significant crosstalk between the PI3K/AKT/mTOR and RAS/MAPK pathways, which can influence each other negatively or positively (Aksamitiene et al., 2012). Furthermore, *Erk1/2* double knockout mice showed reduced myelination and decreased myelin gene and protein expression, which persisted into adulthood (Ishii et al., 2012). To examine a potential link between the MAPK pathway and the expression of cholesterol biosynthetic genes and myelination in *Pex2*^−/−^ mice, we measured total protein levels and phosphorylation of ERK1/2 in P10 *Pex2*^−/−^ mice compared to controls. Total ERK levels were similar in control and *Pex2*^−/−^ mice, while phosphorylated ERK levels showed a trend toward increase in *Pex2*^−/−^ mice (Figure 10).

Our findings suggest that reduced mTOR activity plays a role in the observed hypomyelination in *Pex2*^−/−^ mice.

### Neuroinflammation in the CNS of *Pex2*^−/−^ mice

**Figure 11:**
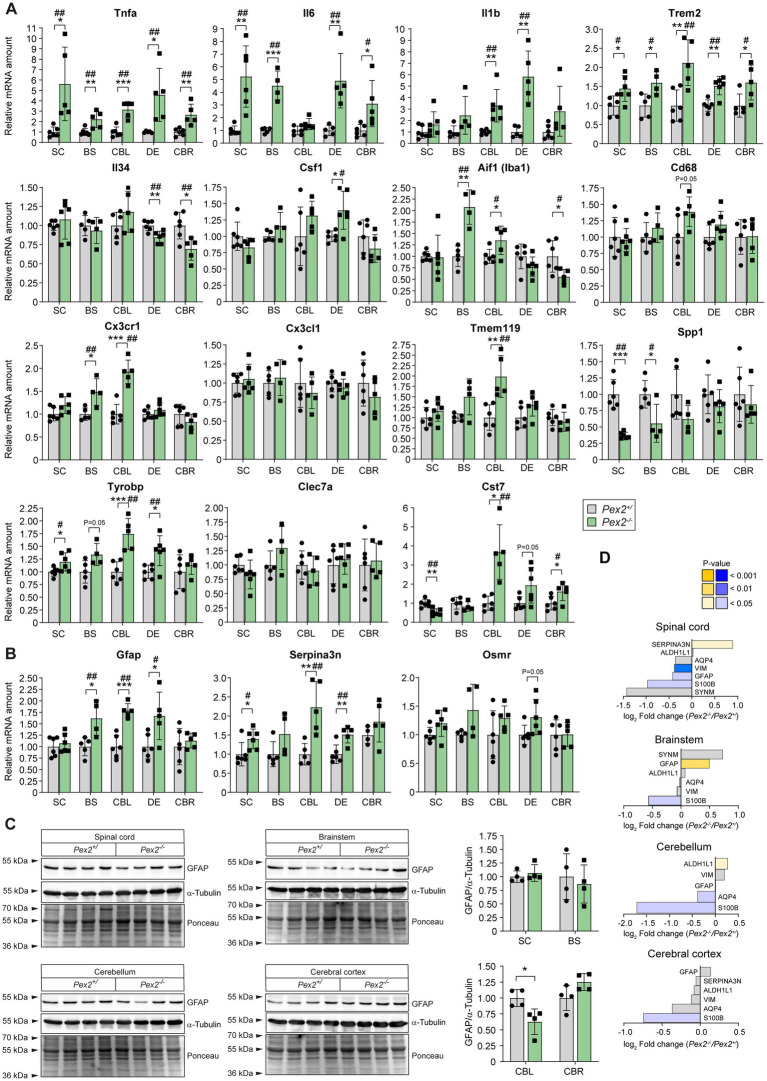
Expression of genes encoding **(A)** pro-inflammatory cytokines and microglial markers and **(B)** astrocytic reactivity markers in the CNS of P10 control and *Pex2*^−/−^ mice. **(C)** Immunoblot analysis of GFAP in whole tissue lysates of P10 control and *Pex2*^−/−^ mice. α-tubulin serves as loading control. Protein ratios are expressed relative to that in control mice, which were arbitrarily defined as 1. **(D)** Protein levels of astrogliosis markers in the SC, BS, CBL, and CBR of P10 control and *Pex2*^−/−^ mice. Blue bars: significantly downregulated proteins; yellow bars: significantly upregulated proteins; gray bars: no significant change. Data are mean ± SD. Statistical analysis was performed using Student’s *t*-test or Student’s *t*-test with Welch’s correction, followed by a multiple comparison correction using the Benjamini–Hochberg procedure. ^*^p < 0.05, ^**^p < 0.01, and ^***^p < 0.001; ^#^adjusted *p* < 0.1 and ^##^adjusted *p* < 0.05; versus the corresponding CNS region in control mice.

Since the expression of *Tspan2*, which plays a role in regulating neuroinflammation by suppressing astrocyte and microglia activity, was reduced in the CNS of *Pex2*^−/−^ mice at P10, we investigated whether inflammation in the CNS might already play a role in P10 *Pex2*^−/−^ mice. Indeed, the expression of the pro-inflammatory cytokines *Tnfa*, *Il6*, and *Il1b* was increased in the CNS of *Pex2*^−/−^ mice (Figure 11A). The expression of *Il10*, which is primarily anti-inflammatory in the brain, was very low and could not be reliably determined. The cytokines CSF1 (colony stimulating factor 1) and IL34 are ligands for the CSF1 receptor, which is essential for microglial development, survival and proliferation. It has been shown that they induce microglial proliferation during neurodegeneration. The expression of *Il34* was reduced in the DE and CBR, and *Csf1* mRNA levels were only increased in the DE (Figure 11A). Next, we examined whether NF-κB (Nuclear Factor kappa-light-chain-enhancer of activated B cells), which is a central transcription factor that is known to regulate the expression of pro-inflammatory cytokines, was activated. As marker for NF-κB activation we determined the phosphorylation of IκB-α at Ser32 (Vallabhapurapu and Karin, 2009). The phosphorylation of IκB-α was increased in the SC and CBR of *Pex2*^−/−^ mice (Supplementary Figures S8A,B).

To gain further insight into neuroinflammation in P10 *Pex2*^−/−^ mice, we examined the expression of homeostatic and disease-associated microglial signature genes. Triggering receptor expressed on myeloid cells 2 (TREM2) is predominantly expressed on microglia and is a critical regulator of their proliferation, survival, phagocytosis, polarization, inflammation, and metabolism (Ulland and Colonna, 2018; Deczkowska et al., 2020; Yu et al., 2025). *Trem2* and its downstream adaptor *Tyrobp/Dap12* (TYRO protein tyrosine kinase-binding protein) belong to the homeostatic microglial genes, and their expression is upregulated in disease-associated microglia (DAM) (Deczkowska et al., 2018). While *Trem2* expression was significantly increased in all CNS regions of *Pex2*^−/−^ mice examined, *Tyrobp* mRNA levels were significantly increased in the CBL, DE, and SC, and tended to increase in the BS (Figure 11A). We also determined the mRNA levels of *Aif1* (allograft inflammatory factor 1, also known as *Iba1*), whose expression is upregulated when microglia become activated. Aif1 expression was significantly increased in the BS and CBL of *Pex2*^−/−^ mice but decreased in the CBR (Figure 11A). DAM state is characterized by the repression of several homeostatic genes, such as the chemokine receptor *Cx3cr1* and *Tmem119*. However, *Cx3cr1* expression was significantly increased in the BS and CBL (Figure 11A). The expression of *Tmem119*, a protein that marks brain-resident microglia but not CNS-associated macrophages (Ruan and Elyaman, 2022), was increased in the CBL of *Pex2*^−/−^ mice, but not in other regions of the CNS (Figure 11A). The expression of *Cx3cl1*, a ligand for CX3CR1 that is primarily produced in neurons, was similar in control and *Pex2*^−/−^ mice (Figure 11A). We also examined the expression of genes involved in the phagocytic activity of microglia which are upregulated in DAM, including *Cd68*, *Clec7a* and *Cst7*. The expression of *Cd68* and *Clec7a* was similar in control and *Pex2*^−/−^ mice, but *Cst7* mRNA levels were increased in the CBL and CBR and decreased in the SC (Figure 11A). Interestingly, the expression of the pro-inflammatory *Spp1* (osteopontin), a gene associated with DAM and the microglial neurodegenerative phenotype (MGnD), was significantly decreased in the SC and BS of *Pex2*^−/−^ mice.

Microgliosis is associated with astrogliosis in CNS pathologies, and it has been hypothesized that activated microglia initiate and maintain astrogliosis (Zhang et al., 2010). We examined the expression of astrocytic reactivity markers *Gfap* (glial fibrillary acidic protein), *Osmr*, and *Serpina3n* (Figure 11B). The mRNA levels of *Gfap* were significantly higher in the BS, CBL, and DE of *Pex2*^−/−^ mice, while *Serpina3n* expression increased in the SC, CBL and DE (Figure 11B). Osmr mRNA levels were similar in control and *Pex2*^−/−^ mice. The mRNA and protein levels of S100B, a marker of astrocyte activation, were significantly decreased in the CNS of *Pex2*^−/−^ mice at P10 (Figures 9, 11D; Supplementary Figure S7B). Next, we investigated by Western blotting whether the changes in the expression of *Gfap* were also reflected at the protein level. Western blot analysis showed that GFAP protein levels were similar in the SC, BS and CBR, but decreased in the CBL (Figure 11C). In contrast, proteomics analysis showed that GFAP levels decreased in the SC and increased in the BS (Figure 11D). We also examined the protein levels of additional astrogliosis markers (Zhang et al., 2010; Jurga et al., 2021), including vimentin (VIM), ALDH1L1 (aldehyde dehydrogenase 1 family member L1), aquaporin 4 (AQP4) and synemin (SYNM), in the proteomics data set. However, no pronounced increase in astrogliosis marker protein levels was observed. SERPINA3N was significantly increased in SC, GFAP in BS and ALDH1L1 in CBL (Figure 11D). Conversely, VIM and AQP4 protein levels were significantly decreased in SC and CBL, while the levels of other proteins that did not show significant changes tended to decrease (Figure 11D). SYNM is an intermediate filament protein that associates with GFAP and vimentin in astrocytes, and several studies have shown that SYNM expression increases in reactive astrocytes (Jing et al., 2007; Potokar et al., 2020). SYNM protein levels showed a downward trend in the SC and an upward trend in the BS (Figure 11D).

In summary, the expression of pro-inflammatory cytokines is increased and the expression of some homeostatic and disease-associated microglial genes has changed. However, full DAM activation is not yet observed in P10 *Pex2*^−/−^ mice.

## Discussion

**Figure 12:**
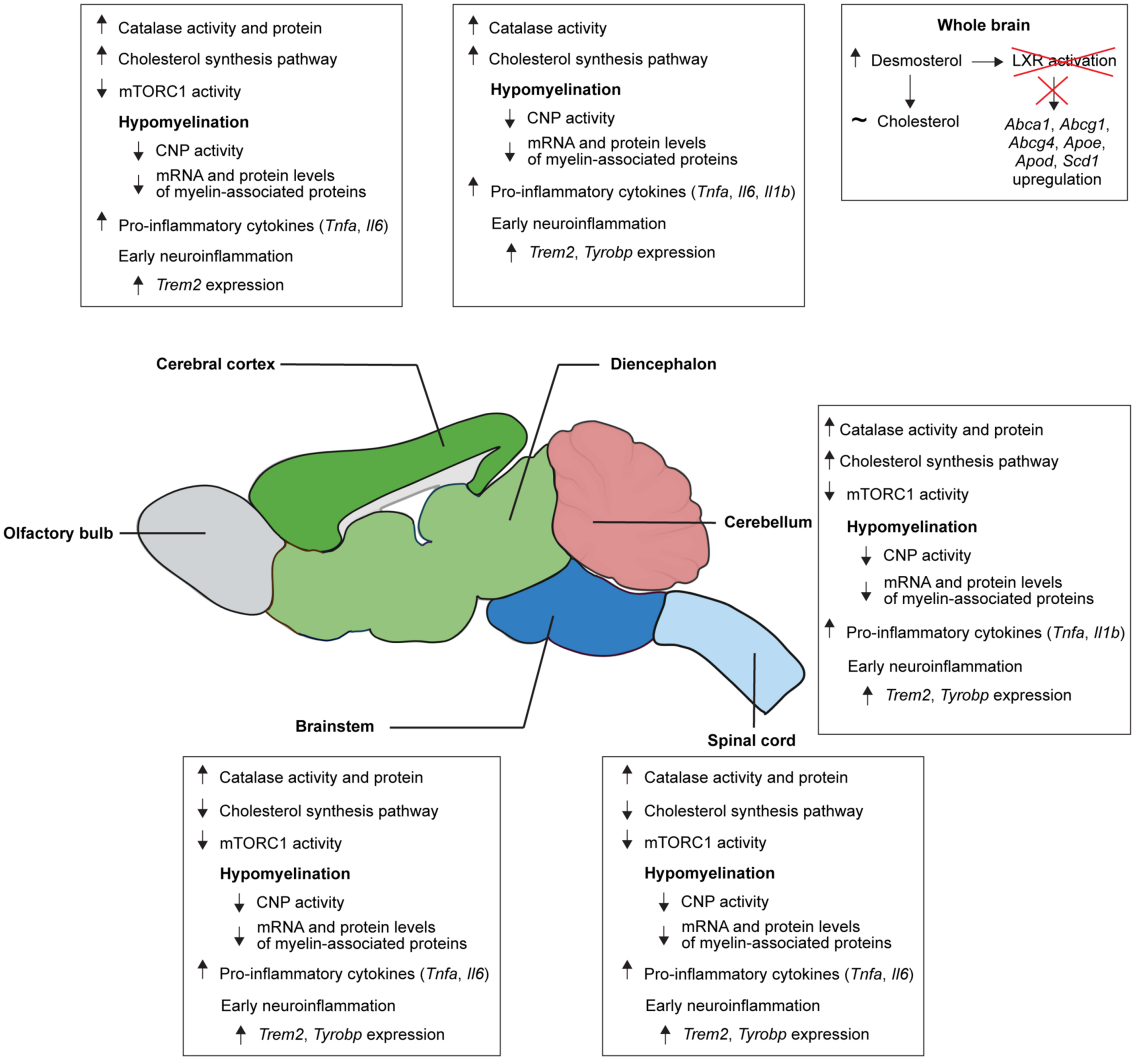
Model illustrating how peroxisome deficiency affects cholesterol biosynthesis, myelination, and neuroinflammation in different CNS regions of P10 *Pex2*^−/−^ mice. Desmosterol levels—a precursor in the Bloch pathway—were significantly increased in the brain of *Pex2*^−/−^ mice. Although desmosterol is a potent LXR agonist, the expression of apolipoproteins and lipid transporters, which are positively regulated by LXRs, was downregulated in *Pex2*^−/−^ mice. We show through enzyme activity measurements and the determination of mRNA and protein levels that the cholesterol biosynthesis pathway is downregulated in CNS regions with a higher proportion of white matter (i.e., SC, BS) and upregulated in regions enriched for gray matter (i.e., cerebral cortex). The systemic deficiency of functional peroxisomes in P10 *Pex2*^−/−^ mice results in severe hypomyelination in the CNS, evidenced by decreased activity of CNP, as well as decreased mRNA and protein levels of major myelin structural proteins and other myelin-associated proteins. The consistent decrease in RPS6 phosphorylation in the CNS of *Pex2*^−/−^ mice suggests that decreased mechanistic target of rapamycin complex 1 (mTORC1) activity contributes to hypomyelination. Although the expression of pro-inflammatory cytokines has increased and the expression of some homeostatic and disease-associated microglial genes has changed, full DAM activation has not yet been observed in P10 *Pex2*^−/−^ mice.

Myelination is a complex, developmentally regulated process that involves the coordinated expression of genes encoding myelin proteins and lipid biosynthetic enzymes for the synthesis of myelin-specific lipids, particularly cholesterol and sphingolipids. In the CNS the majority of cholesterol biosynthesis must be coordinated with myelin formation, in contrast to extraneuronal tissues where cholesterol synthesis is regulated by feedback mechanisms related to cholesterol levels (Horton et al., 2002; Loewen and Levine, 2002; Goldstein et al., 2006). In our previous study we showed that the rate of cholesterol synthesis is decreased in the brain of P10 *Pex2*^−/−^ mice (Kovacs et al., 2004). Here we show through enzyme activity measurements and the determination of mRNA and protein levels that the cholesterol biosynthesis pathway is downregulated in CNS regions with a higher proportion of white matter (i.e., SC, BS) and upregulated in regions enriched for gray matter (i.e., cerebral cortex) (Figure 12). As expected, enzymes involved in peroxisomal fatty acid β-oxidation and etherlipid synthesis were markedly decreased at the protein level in the CNS of P10 *Pex2*^−/−^ mice. In contrast, enzymes involved in mitochondrial fatty acid β-oxidation were generally not significantly altered. Furthermore, enzymes involved in the synthesis and elongation of fatty acids and sphingolipid synthesis were downregulated in the CNS of *Pex2*^−/−^ mice. Changes in the expression of proteins involved in cholesterol and fatty acid synthesis mainly occur postnatally, as no or only minor changes were observed in newborn mice. A central finding of this study is that the systemic deficiency of functional peroxisomes in P10 *Pex2*^−/−^ mice results in severe hypomyelination in the CNS, evidenced by decreased activity of CNP, as well as decreased mRNA and protein levels of major myelin structural proteins and other myelin-associated proteins (Figure 12). This level of severe hypomyelination, which is not caused by demyelination, has not been observed in conditional models (Kassmann et al., 2007; Hulshagen et al., 2008; Bottelbergs et al., 2010) or whole-body knockout models (Huyghe et al., 2006; Brites et al., 2009) of peroxisomal disorders at this stage of development.

Diseases affecting myelin can be divided into those that specifically target myelin, referred to as primary diseases of myelin, and those that non-specifically affect the white matter of the CNS (i.e., secondary demyelination) (Faust et al., 2010). Primary myelin diseases are further divided into those with demyelination versus dysmyelination. In demyelination, abnormalities in myelin occur after normal myelination has taken place, the most classic example being the disease multiple sclerosis. Dysmyelinating diseases are typically inherited genetic disorders that lead to an abnormal biochemical composition of myelin and/or paucity in its initial formation (i.e., hypomyelination). Our data in *Pex2*^−/−^ mice show severe hypomyelination throughout the CNS of *Pex2*^−/−^ mice at P10, the active phase of myelination, suggesting that systemic peroxisome deficiency belongs to the dysmyelination category of primary myelin diseases as there is abnormal myelin formation or maintenance.

In humans, it has been suggested that the myelin lesions in Zellweger syndrome are in part hypomyelinative, but probably also reflect some secondary myelin loss due to the severe systemic abnormalities from which these infants suffer (Faust et al., 2010). Postnatal peroxisome-deficient *Pex2*^−/−^ mice suffer from intestinal lipid malabsorption due to impaired bile acid synthesis (Keane et al., 2007), and the combination of inadequate dietary fat intake and impaired fatty acid processing can lead to severe energy deficits and developmental delays such as hypomyelination. Interestingly, we did not observe AMPK activation, which is an indicator of low cellular energy status, in the CNS of P10 *Pex2*^−/−^ mice. Oral bile acid treatment increased survival and alleviated intestinal malabsorption in *Pex2*^−/−^ mice (Keane et al., 2007) and improved some but not all aspects of CNS development (Faust et al., 2005), suggesting a role for nutritional status in CNS function. While BA treatment led to a variable but significant improvement in the extent of Purkinje-cell dendritic arborization, BA-treated *Pex2*^−/−^ mice still showed the spastic hind limb crossing seen in untreated mutant mice when hung by their tails (Faust, 2003; Faust et al., 2005). Prior studies have shown that starvation from birth to 14–20 days results in persistent, significant myelin deficits in the cerebrum, cerebellum, medulla, midbrain, hippocampus, hypothalamus, and striatum of the rat brain (Wiggins et al., 1976; Wiggins and Fuller, 1978). Starvation early in development, during the period of oligodendroglial cell proliferation, causes an immediate reduction in myelin synthesis, and the resulting myelin deficit proves irreversible upon subsequent nutritional rehabilitation (Wiggins and Fuller, 1978). Another study also showed that hypomyelination of the rat brain resulting from early nutritional deprivation is characterized by a specific reduction in myelin membrane synthesis rather than increased turnover (Royland et al., 1993).

Previous studies have investigated myelination using conditional knockout mouse models of peroxisomal disorders. These studies found that myelination was affected differently and at a later stage than in our study, in which P10 *Pex2*^−/−^ mice with a systemic peroxisome deficiency were used. In X-ALD patients, myelin lesions primarily involve inflammatory demyelination, and milder degrees of inflammatory demyelination are also observed in neonatal adrenoleukodystrophy and MFP2 (HSD17B4, peroxisomal multifunctional protein 2) deficiency (Faust et al., 2010; Berger et al., 2016). However, mouse models for X-ALD by targeted inactivation of the *Abcd1* gene did not experience brain inflammation and demyelination as seen in humans with cerebral ALD (Berger et al., 2016). Myelin anomalies were only detectable in the SC and sciatic nerve after 18 months of age, but not in the brain (Pujol et al., 2002). The importance of peroxisomes for the maintenance of myelin and axonal integrity was demonstrated in a mouse model with *Nestin-Cre*-directed deletion of the *Pex5* gene in all neural precursor cells at embryonic stages (Hulshagen et al., 2008; Bottelbergs et al., 2010, 2012). These mice form abnormal myelin, decreased myelin sheath thickness, abnormal compaction and irregularities at the node of Ranvier, and develop early (3 weeks), progressive axonal damage and myelin loss. Some fibers lacked myelin in the cerebellar folia at the age of 2 weeks (Bottelbergs et al., 2012). These alterations were accompanied by an accumulation of VLCFAs and a depletion of plasmalogens. A mouse model with *CNP-Cre*-directed conditional deletion of *Pex5* in oligodendrocytes showed normal myelination at the ultrastructural level, but inflammatory demyelination developed with disease onset after myelination (by 3 months) (Kassmann et al., 2007). This mouse model most closely mimics human cerebral ALD and demonstrates the importance of oligodendroglial peroxisomes in the maintenance of brain myelin (Kassmann et al., 2007). Interestingly, axonal integrity and normal behavior was preserved when peroxisomes were deleted from astrocytes (*Gfap-Pex5* mutants) or neurons (*NEX-Pex5* mutants), and staining for myelin was normal in 12- to 15-month-old knockout mice (Bottelbergs et al., 2010). However, an accumulation of VLCFAs and a reduction of plasmalogens were observed in isolated myelin of *Gfap-Pex5*^−/−^ mice, indicating that peroxisomal metabolites are shuttled between different brain cell types (Bottelbergs et al., 2010). A mouse model deficient in both *Abcd1* and *Pex7* shows moderate myelin loss by 10 months of age that is not seen in single knockout mice, demonstrating an interaction between plasmalogen loss and VLCFA-induced pathology (Brites et al., 2009). *Hsd17b4* knockout mice show a similar clinical course and axonal damage as *Nestin-Pex5*^−/−^ mice, but myelin lipid and protein staining is preserved (Huyghe et al., 2006). Liver-specific deletion of *Pex5* using the albumin/α-fetoprotein (*Alfp*)-*Cre* transgene had a severe and permanent impact on cortical neuronal migration and cerebellar maturation; however, the effect on myelination has not been investigated in these mice (Krysko et al., 2007). Reconstitution of peroxisomes in the liver of *Pex5*^−/−^ mice by overexpressing *Pex5* resulted in partial rescue of the brain defects and was not accompanied by changes in VLCFAs, DHA, or plasmalogen levels in brain, indicating that brain-extrinsic effects influence the CNS development in peroxisomal disorders (Janssen et al., 2003).

Altered signal transduction pathways are also likely to contribute to hypomyelination in *Pex2*^−/−^ mice, particularly the significantly reduced mTORC1 activity, which was not investigated in earlier studies with conditional *Pex5* and *Pex13* knockout mice. Interestingly, while studies showed that the spinal cord seems more vulnerable to the deletion of mTOR than the brain and that the loss of mTOR has differential impact on oligodendrocyte development in different CNS regions (Bercury and Macklin, 2015; Yu et al., 2023), we observed a downregulation of mTORC1 signaling and hypomyelination in all CNS regions of P10 *Pex2*^−/−^ mice (Figure 12). Together, the reduced p-AMPK at P10 and the consistent decrease in p-RPS6 suggest that hypomyelination is primarily driven by decreased mTORC1 activity rather than by AMPK-mediated inhibition. Downregulation of both myelin proteins and mRNA, as observed in *Pex2*^−/−^ mice, was also detected in oligodendrocyte-specific *Raptor* knockout mice with impaired mTORC1 function. Although mTORC1 is classically known as a regulator of protein translation, the downregulation of myelin proteins and mRNAs in *Raptor* knockout mice showed that mTORC1 plays a key role in mediating myelination at the translational and transcriptional levels (Bercury et al., 2014; Lebrun-Julien et al., 2014).

mTORC1 was also shown to modulate the activity of mature SREBPs, and nuclear accumulation of SREBPs was inhibited when mTORC1 was suppressed. However, despite the downregulation of mTORC1 signaling, the SREBP-2 pathway was upregulated in gray matter regions of P10 *Pex2*^−/−^ mice. A recent study suggested that acetyl-CoA derived from peroxisomal β-oxidation promotes Raptor acetylation and mTORC1 activation in hepatocytes, and hepatic *Acox1* deficiency resulted in inhibition of mTORC1 (He et al., 2020). Therefore, it is reasonable to hypothesize that peroxisome-derived acetyl-CoA also contributes to mTORC1 activation in the CNS.

It has been shown that during the first 3 weeks after birth, the rates of cholesterol synthesis are significantly higher in the spinal cord and brainstem than in the cerebral cortex and cerebellum. These predominantly white matter regions also attain the highest cholesterol concentrations (Turley and Dietschy, 1997; Turley et al., 1998; Quan et al., 2003). Therefore, the rate of cholesterol accumulation in each CNS region is closely correlated with the rate of cholesterol synthesis. Despite hypomyelination and a decreased rate of cholesterol synthesis in the brain of *Pex2*^−/−^ mice (Kovacs et al., 2004), total cholesterol levels were comparable in control and *Pex2*^−/−^ mice. In contrast, desmosterol levels - a precursor in the Bloch pathway - were significantly increased in *Pex2*^−/−^ mice. Although BA feeding increased the rate of cholesterol synthesis in the *Pex2*^−/−^ brain to the level of control mice (Kovacs et al., 2009), desmosterol levels remained elevated as in untreated mice. This indicates that sterol metabolism is dysregulated in the peroxisome-deficient brain. Interestingly, the protein levels of DHCR24, which catalyzes the conversion of desmosterol to cholesterol, were significantly decreased in the SC and BS, while in the CBL and CBR only DHCR7 was detected. In fact, there is evidence that glial cells (which are abundant in white matter) predominantly use the Bloch pathway, while neurons (abundant in gray matter) preferentially use the Kandutsch–Russell pathway (Figure 3B) (Nieweg et al., 2009). The significant downregulation of the cholesterol biosynthetic pathway in the white matter regions SC and BS, alongside a significant upregulation in the CBR, a predominantly gray matter region, highlights the complex and region-specific metabolic adaptations to peroxisome deficiency (Figures 3C,D, 4A; Supplementary Figure S4A). A limitation of our study is that we analyzed the entire tissue of different regions of the CNS, so that observed changes in the activity of enzymes, or the expression of genes and proteins cannot be assigned to a specific cell type (neurons, astrocytes, oligodendrocytes, and microglia). However, it can be assumed that the downregulation in the white matter regions affects the myelinating oligodendrocytes, while the upregulation in the gray matter regions is due to neurons and astrocytes.

Desmosterol is found in the developing brain of many species, but it is absent or present at low levels in the mature CNS (Dennick et al., 1974; Jansen et al., 2013; Allen et al., 2019). It accounts for up to 30% of total sterols and 80% of newly synthesized sterols in the CNS of newborn mice (Jurevics and Morell, 1995; Jansen et al., 2013; Saher and Stumpf, 2015) and represents an important constituent of membranes and lipoproteins in the developing brain (Mutka et al., 2004). The highest desmosterol levels were detected before and during the early stages of myelination, whereas desmosterol decreases as cholesterol levels increase during the period of active myelination (Fumagalli and Paoletti, 1963; Dennick et al., 1974; Saher and Stumpf, 2015). Desmosterol is released from cells more efficiently than cholesterol (Spann et al., 2012), and since desmosterol synthesis is high in young mice, it is conceivable that oligodendrocytes are supplied with desmosterol during myelination. Despite its small structural differences, desmosterol behaves differently from cholesterol and may have a distinct function (Jansen et al., 2013). It has a lower affinity to caveolin and perturbs caveolar morphology (Jansen et al., 2008), and is less efficient in promoting membrane order (Megha et al., 2006; Vainio et al., 2006). Studies with cortical neuronal cultures from embryonic day 15 showed that elevated desmosterol levels increased the arborization of neurons compared to the neuronal outgrowth in control cells (Allen et al., 2019). Since desmosterol is a potent LXR agonist (Yang et al., 2006; Spann et al., 2012), one would expect upregulation of cholesterol efflux genes. Instead, we observed downregulation of apolipoproteins and lipid transporters (e.g., ABCA1) in *Pex2*^−/−^ mice. This implies that there is impaired intercellular sterol transport, which may contribute to the observed hypomyelination.

Myelination has been studied using conditional knockout mouse models of cholesterol biosynthesis. The lack of cholesterol synthesis in oligodendrocyte-specific *Cnp-Fdft1* knockout mice results in a severely delayed onset of myelination, but myelination is able to proceed to completion, albeit at a slower rate (Saher et al., 2005). A study using *Cnp-Cre Scap* knockout mice showed that *Scap* deletion affected the acute phase of myelination, i.e., CNS myelination was significantly delayed, but myelin appeared normal at 3 months of age (Camargo et al., 2017). Inactivation of *Hmgcs1* in zebrafish caused OPCs to migrate past their target axons and to fail to express myelin genes (Mathews et al., 2014). Using a combination of pharmacological inhibitor and rescue experiments, it was shown that isoprenoids and protein prenylation, but not cholesterol, were required in OPCs to halt their migration at target axons (Mathews et al., 2014). Cholesterol, but not isoprenoids, was required for axon ensheathment and myelin gene expression (Mathews et al., 2014). In contrast, impaired cholesterol synthesis in astrocyte-specific *Scap* knockout mice results in persistent hypomyelination, demonstrating an important role for astrocytes in contributing to the cholesterol and fatty acid pool required for myelination (Camargo et al., 2017). Cholesterol metabolism in astrocytes is also a major contributor to neuronal cholesterol homeostasis. Astrocyte-specific *Scap* knockout mice showed reduced numbers of synaptic vesicles and defective synaptic plasticity (van Deijk et al., 2017). In addition, astrocyte-specific *Srebf2* knockout resulted in reduced neurite outgrowth and impaired brain development, as well as behavioral and motor defects (Ferris et al., 2017). Although the blood–brain barrier is not yet fully formed in the early developmental phase, as in our study, it has been shown that the brain produces almost all of its own cholesterol (Saher and Stumpf, 2015; Montani, 2021). Oligodendrocytes can synthesize their own cholesterol or obtain it from astrocytes, which are known to distribute lipids via lipoproteins.

ORA and GSEA analysis revealed that fatty acid metabolism is severely impaired in *Pex2*^−/−^ mice. The blood–brain-barrier is permeable to fatty acids and allows the transport of both essential and non-essential fatty acids into the CNS (Montani, 2021). OPCs do not express FASN at detectable levels, and FASN depletion from OPC cells did not affect their proliferation and differentiation into the oligodendrocyte lineage (Dimas et al., 2019). However, endogenous fatty acid synthesis is essential to establish the correct myelin lipid composition and myelin growth (Dimas et al., 2019). We observed significant changes in the mRNA and protein levels of enzymes involved in fatty acid synthesis, particularly in fatty acid elongation, and sphingolipid metabolism. Myelin formation requires the synthesis of large amounts of sphingolipids, particularly sphingomyelin (Baumann and Pham-Dinh, 2001). mRNA and protein levels of UGT8 were significantly decreased in the CNS of *Pex2*^−/−^ mice. Hypomyelination and unstable myelin sheaths have been reported in *Ugt8*^−/−^ mice (Dupree et al., 1998). In addition, mRNA and protein levels of FA2H were significantly decreased in the CNS of *Pex2*^−/−^ mice. Mutations in *FA2H* have been associated with leukodystrophy and spastic paraparesis in humans, underscoring the importance of 2-hydroxy fatty acid-sphingolipids in the nervous system (Hama, 2010; Eckhardt, 2023). CERS2, which catalyzes synthesis of ceramides with a preference for VLCFAs (C22:0–C24:0) (Imgrund et al., 2009), and DEGS1, which catalyzes the final step of *de novo* ceramide biosynthesis, were significantly decreased in white matter regions of *Pex2*^−/−^ mice. It has been shown that *Cers2* is expressed in peripheral and central myelinating cells, and its expression transiently increased during the period of active myelination (Becker et al., 2008). Accordingly, the quantity of compact myelin and MBP isolated from 10-week-old *Cers2* knockout mice was significantly reduced (Imgrund et al., 2009). The loss of DEGS1 impairs nervous system function in humans and causes hypomyelinating leukodystrophy (Karsai et al., 2019; Pant et al., 2019). Several sphingomyelin species were found to be reduced in the plasma of patients with ZSD, but the cause is unknown (Wangler et al., 2018). This finding and our observations suggest that peroxisomes play a role in sphingomyelin metabolism, which warrants further investigation.

An interesting finding was the significant upregulation of HMGCS2 (Figures 3E, 4A), which is a key enzyme of ketone body synthesis and is most strongly associated with the liver, in the CNS of *Pex2*^−/−^ mice. Ketone bodies serve as an alternative energy source for the brain, particularly when glucose is scarce during prolonged fasting or carbohydrate restriction. Oligodendrocytes preferentially use ketone bodies rather than glucose as a substrate for cholesterol synthesis (Koper et al., 1981). Recent single-cell and tissue expression data (e.g., the Allen Brain Atlas) indicate low but detectable expression of HMGCS2 in astrocytes, with minimal to no expression in neurons, oligodendrocytes or microglia. This aligns with the role of astrocytes in metabolic support and ketone body metabolism in the brain (Blázquez et al., 1998; Cullingford et al., 1998). It is possible that under certain conditions (e.g., injury, inflammation, metabolic stress) *Hmgcs2* may be induced in glial cells. In fact, *Hmgcs2* expression is markedly upregulated and drives metabolic reprogramming in the brain nucleus accumbens of mice exposed to cocaine (Shao et al., 2019). Furthermore, studies have shown that HMGCS2 promotes the autophagic degradation of the β-amyloid precursor protein and tau through ketone body-mediated mechanisms (Hu et al., 2017, 2023). Further studies are necessary to investigate the role of HMGCS2 in the peroxisome-deficient CNS, but the observed upregulation supports the concept of metabolic rewiring in astrocytes toward alternative substrates.

The increased expression of the pro-inflammatory cytokines *Tnfa*, *Il6* and *Il1b* and the altered expression of microglial signature genes (e.g., *Trem2*, *Tyrobp*, *Aif1*) in the CNS of P10 *Pex2*^−/−^ mice point to early neuroinflammatory processes (Figure 12). The increased phosphorylation of IκB-α in the SC and CBR further supports NF-κB activation, a hallmark of microglial activation and neuroinflammation. However, the lack of full DAM activation at P10 suggests that neuroinflammation may progress with disease severity, as seen in other neurodegenerative models. The extent of neuroinflammation in patients with peroxisomal biogenesis disorders has not been thoroughly studied (Sarkar and Lipinski, 2024). Neuroinflammation has been observed in mouse models of peroxisomal disorders, such as oligodendrocyte- and neural-specific *Pex5*^−/−^ mice, albeit only in older animals (>3 months of age) (Kassmann et al., 2007; Hulshagen et al., 2008; Bottelbergs et al., 2010, 2012). The degeneration of myelinated axons in these knockout mice was accompanied with extensive astro- and microgliosis. In contrast, signs of neuroinflammation were absent in *NEX*- and *GFAP-Pex5*^−/−^ mice (Bottelbergs et al., 2010). Increased staining for GFAP and IBA1 has also been observed in the cerebellum of *Nestin-Pex13*^−/−^ mice at P20 (Müller et al., 2011). Neuroinflammation is frequently associated with oxidative stress (Sarkar and Lipinski, 2024). Upregulation of catalase in white matter areas has been observed from an age of 3 weeks in *Nestin-Pex5*^−/−^ mice (Hulshagen et al., 2008; Bottelbergs et al., 2010), and in the CNS of *Pex2*^−/−^ mice catalase was significantly increased at P10. Little is known about the causative metabolic abnormalities underlying neurodegeneration and neuroinflammation in peroxisomal disorders. For example, a strong and chronic pro-inflammatory reaction proceeded throughout the brain of systemic *Hsd17b4*^−/−^ mice, whereas *Nestin-Hsd17b4* knockout mice showed only minor neuroinflammation (Verheijden et al., 2013; Beckers et al., 2018). However, the striking difference in microgliosis between these genotypes was not correlated with altered levels of peroxisomal substrates such as VLCFAs.

The anti-inflammatory effects of TREM2 are widely accepted (Deczkowska et al., 2020), and studies have shown that desmosterol-activated LXR signaling can reduce neuroinflammation in demyelinating diseases, creating an environment favorable for remyelination (Berghoff et al., 2021). LXRs ability to repress inflammatory gene expression derives from their ability to regulate lipid metabolism, and ABCA1 is a critical mediator of LXRs anti-inflammatory effects (Ito et al., 2015). As no activation of the LXR signaling pathway was observed in *Pex2*^−/−^ mice, with the expression of LXR target genes (i.e., *Abca1*, *Apoe*, *Abcg4*, *Scd1*) either reduced or unchanged, we suspect a discrepancy between desmosterol accumulation and canonical LXR responses (Figure 12). LXR signaling is either functionally impaired or pro-inflammatory cues override LXR activity. One possibility is that NF-κB-mediated signaling dominates over LXR transcriptional programs, since pro-inflammatory cytokines can antagonize LXR target gene expression (Joseph et al., 2003). Alternatively, desmosterol’s localization within specific subcellular pools may limit its ability to engage nuclear LXR receptors.

The upregulation of pro-inflammatory cytokines in the CNS of *Pex2*^−/−^ mice should not be considered solely as downstream consequences of metabolic failure from peroxisomal dysfunction, but rather as drivers of a self-amplifying pathological loop. An accumulation of danger signals (e.g., VLCFAs, ROS) would lead to the activation of microglia and astrocytes and the release of cytokines. This, in turn, would induce oligodendrocyte stress, leading to impaired myelin formation and demyelination. Importantly, inflammation in this context may not only exacerbate hypomyelination but also contribute to neuronal stress, consistent with our proteomic enrichment of neurodegenerative pathways.

It should also be noted that, unlike *Nestin-Pex5* and *Cnp-Pex5* knockout mice, functional peroxisomes are also absent in microglia in *Pex2*^−/−^ mice, and microglial peroxisomal dysfunction could contribute to the phenotype observed in the CNS of *Pex2*^−/−^ mice. The effects of peroxisomal defects have been studied mainly in the murine BV-2 microglial cell model deficient for the peroxisomal proteins ACOX1, ABCD1, and ABCD2 (Raas et al., 2019a, 2019b, 2023; Tawbeh et al., 2023, 2025). It was shown that HSD17B4-deficient microglia of *Cx3cr1-Hsd17b4* knockout mice adopt an inflammatory activated and proliferative state in a genetically intact CNS environment, but this change did not affect neuronal function, motor function, cognition, and microglial response to injury (Beckers et al., 2019). Mutant BV-2 cells upregulate the expression of *Trem2* and *Tyrobp* and adopt a DAM-like signature, which is accompanied by reprogramming of lipid metabolism, signaling, and lysosomal and autophagic genes. Additionally, it was demonstrated that peroxisomal dysfunction in these cells exacerbates the inflammatory response and ROS/NOS production, affecting the survival of neurons and oligodendrocytes, as well as neuronal morphology and function. Recently, it was shown that peroxisome deficiency in microglia of *Cx3cr1-Pex5* knockout mice had a minimal impact on homeostatic microglia (Barnes-Vélez et al., 2025). However, peroxisomal integrity is necessary during cuprizone-induced demyelination to enable the clearance of cellular debris and the renewal of myelin (Barnes-Vélez et al., 2025).

In conclusion, the postnatal survival of SW/129 *Pex2*^−/−^ mice allowed the study of myelination in its active phase and showed that systemic deficiency of functional peroxisomes leads to severe hypomyelination. While this study offers a detailed snapshot of the CNS at P10, longitudinal analyses would be needed to determine how these early deficits evolve with age and contribute to progressive neurodegeneration. However, such analyses are not feasible due to the severely limited survival of *Pex2*^−/−^ mice beyond P10. This study also provides a novel proteomic resource for future studies of processes that are dysregulated in the CNS due to the absence of functional peroxisomes.

## Data Availability

The datasets presented in this study can be found in online repositories. The names of the repository/repositories and accession number(s) can be found at: https://www.ebi.ac.uk/pride/archive/, PXD063585.

## Footnotes

1
http://www.ebi.ac.uk/pride
