# Supplementary material for: Dysregulated lipid metabolism and hypomyelination in postnatal peroxisome-deficient Pex2 knockout Zellweger mice
Source: Front Mol Neurosci. 2026 Feb 24;19:1636268. doi: 10.3389/fnmol.2026.1636268 (PMC12971716; doi:10.3389/fnmol.2026.1636268)
Supplement: Supplementary file 6 [file Data_Sheet_1.pdf]

SUPLEMENTAL INFORMATION

Dysregulated lipid metabolism and hypomyelination in postnatal peroxisome-deficient *Pex2* knockout Zellweger mice

Tanja Eberhart, Khanichi N. Charles, Brenda Salumbides-Torres, Nia Price, Steven J. Fliesler, Phyllis L. Faust, Werner J. Kovacs

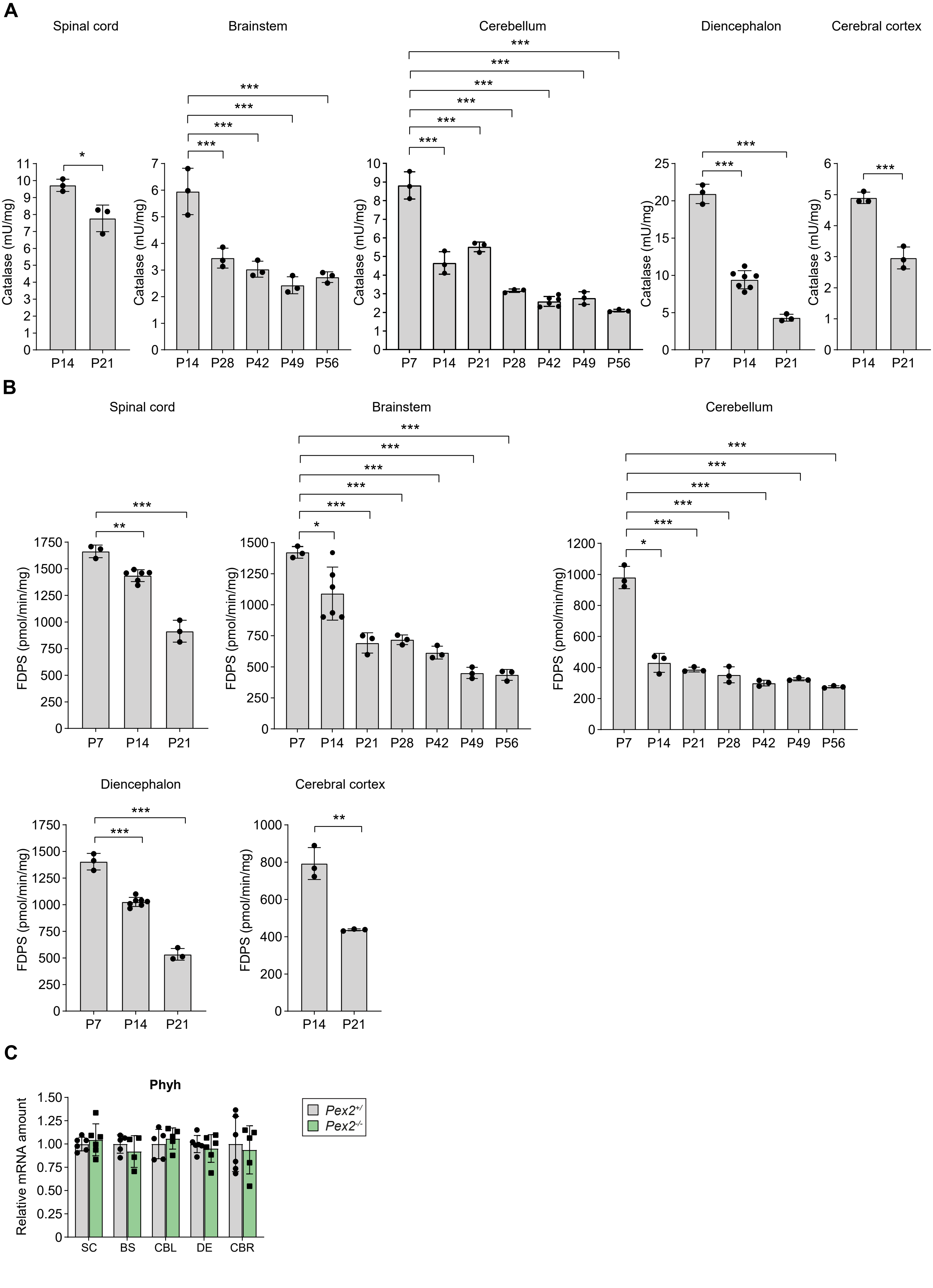

Supplemental Figure 1

Figure S1. (**A**) Catalase activity in the spinal cord (SC), brainstem (BS), cerebellum (CBL), diencephalon (DE) and cerebral cortex (CBR) of P7-56 wild-type BALB/c mice. (**B**) FDPS activity in the SC, BS, CBL, DE, and CBR of P7-56 wild-type BALB/c mice. (**C**) Expression of *Phyh* in the CNS of P10 control and *Pex2*$^{-/-}$ mice. Data are mean ± SD (n = 3-6). Statistical analysis was performed using Student's t-test or Student's t-test with Welch's correction or ordinary one-way ANOVA followed by Tukey's multiple comparisons test. *, P<0.05; **, P<0.01; ***, P<0.001.

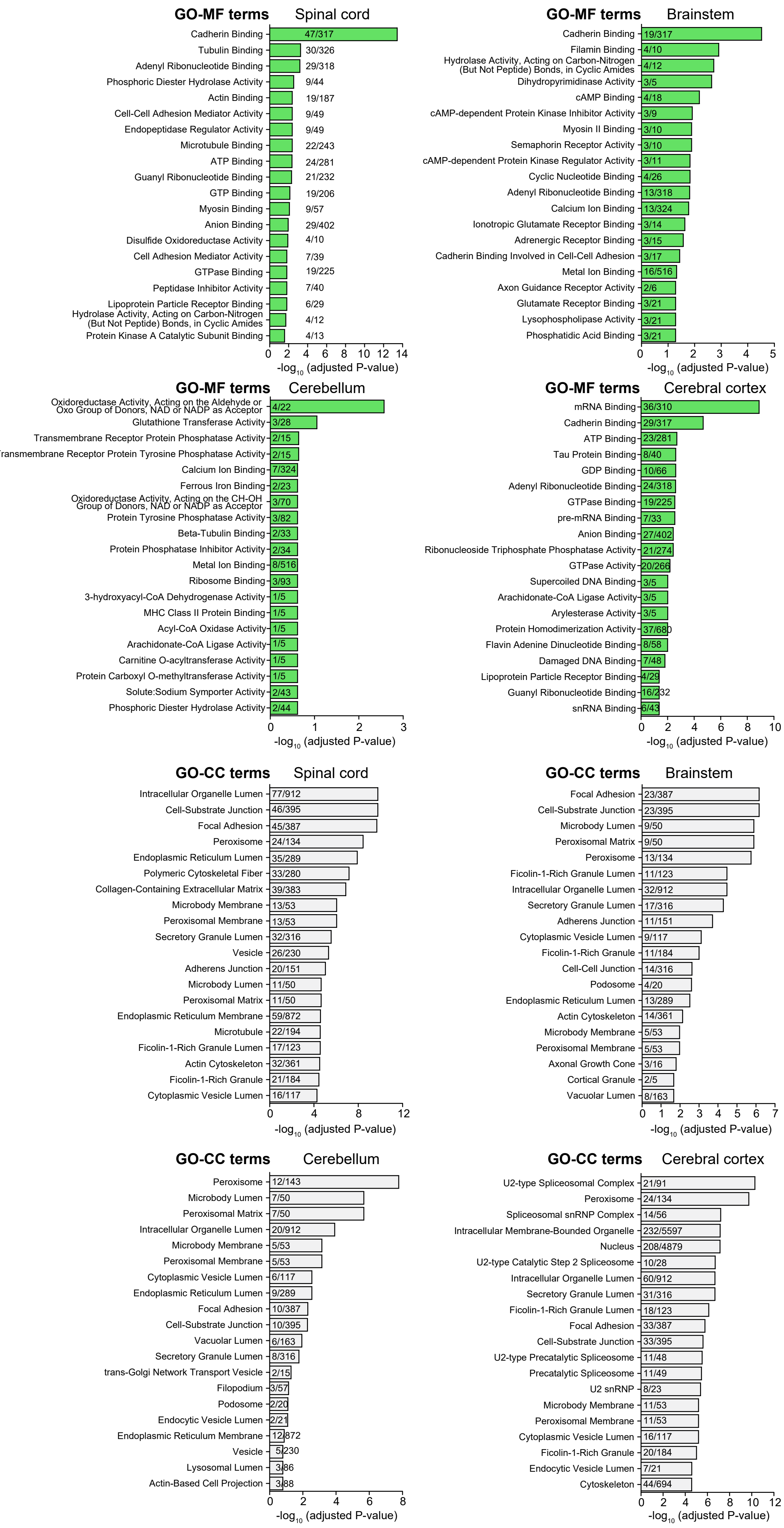

Supplemental Figure 2

Figure S2. Functional analysis of the identified proteins. ORA analysis of differentially expressed proteins in SC, BS, CBL, and CBR of P10 control and *Pex2*$^{-/-}$ mice using the Gene ontology molecular function (GO-MF) and GO cellular compartment (GO-CC) databases. All proteins with an adjusted *P*-value <0.1 were included. No fold change threshold was set, as some of the proteins may show moderate but biologically relevant changes. Numbers in bars indicate differentially expressed proteins compared to the number of proteins in each term.

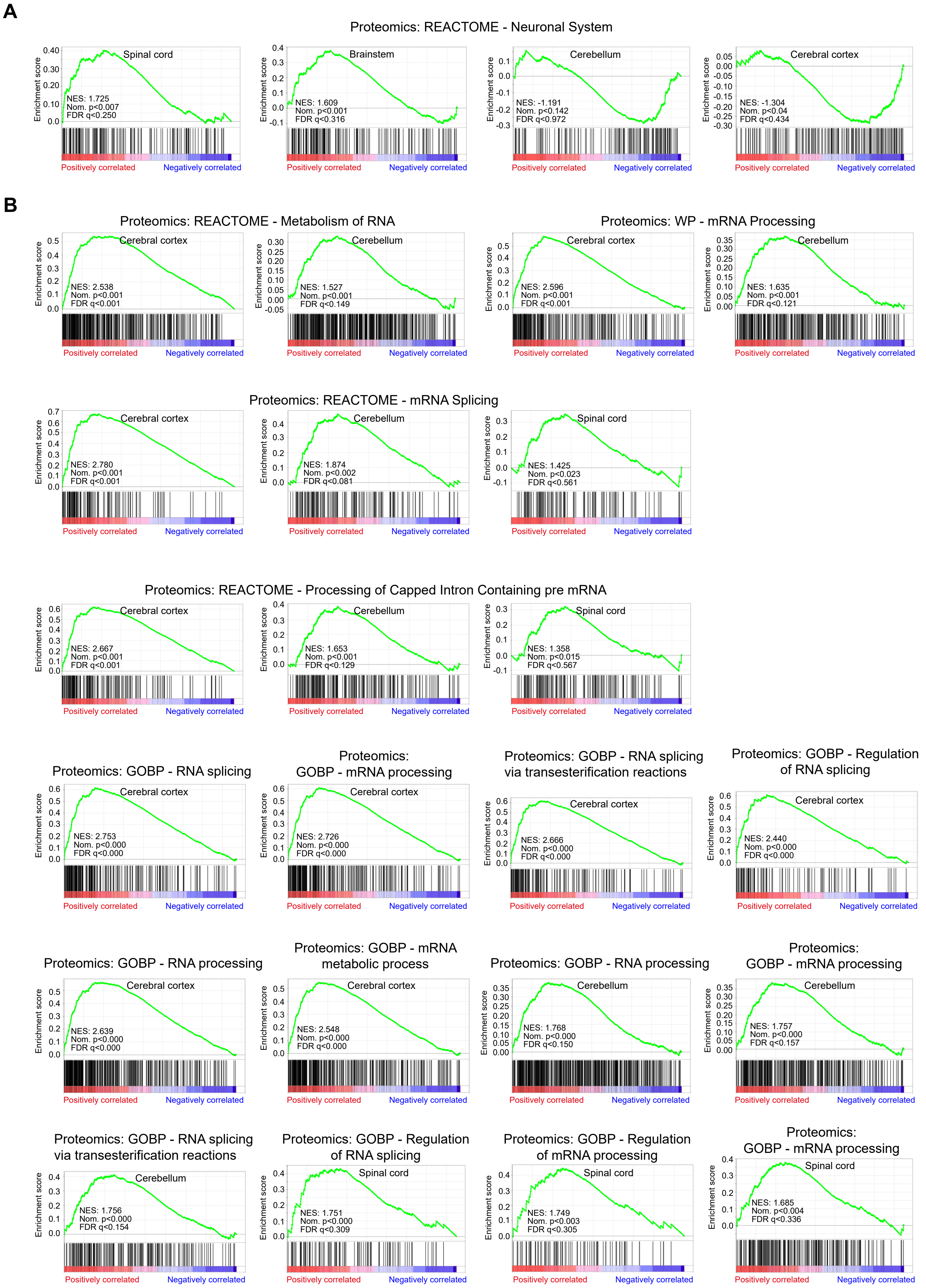

Supplemental Figure 3

Figure S3. GSEA of proteomics data for gene sets related to (**A**) neuronal systems and (**B**) metabolism of RNA, mRNA processing, and mRNA splicing.

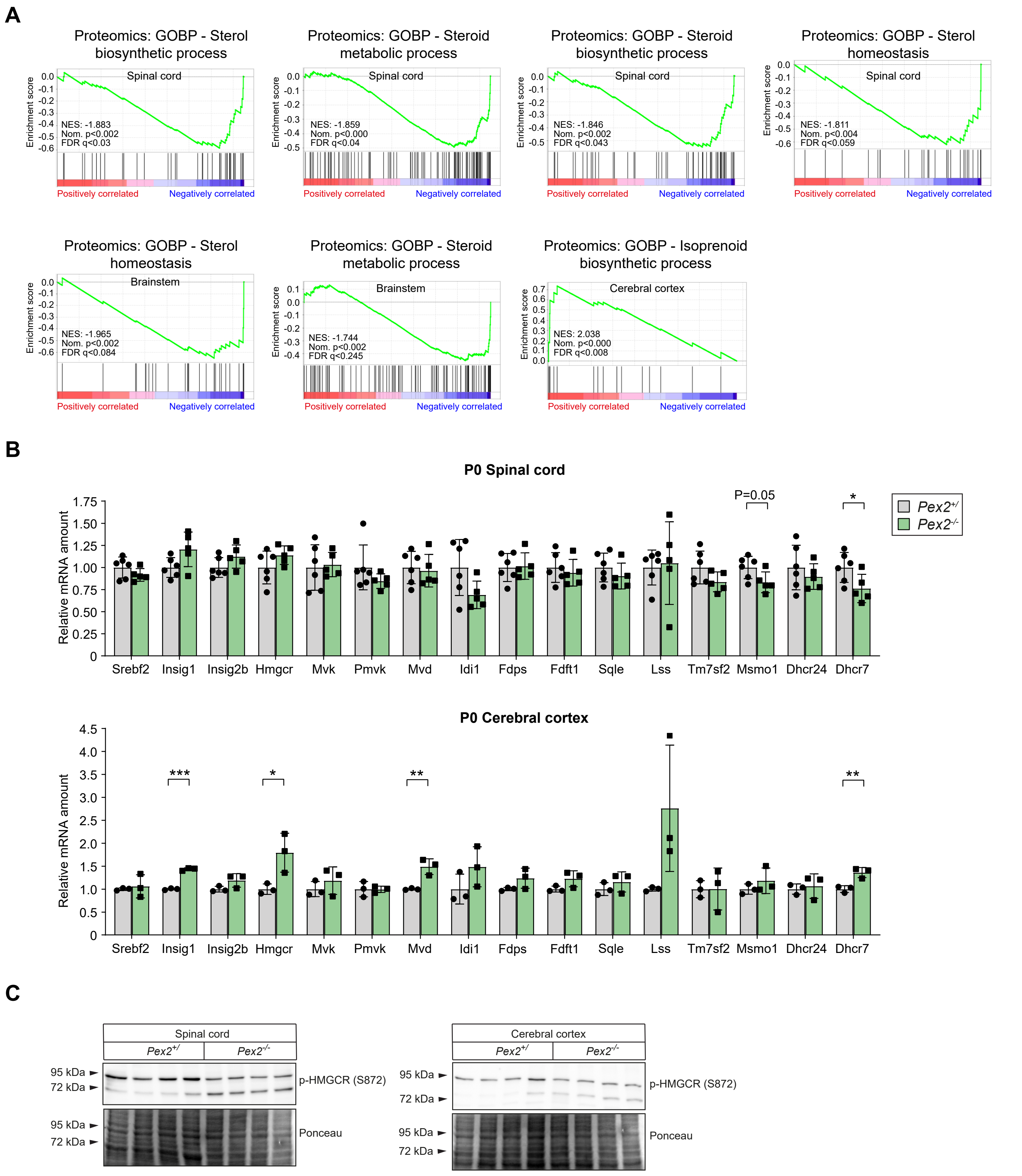

Supplemental Figure 4

Figure S4. (**A**) GSEA of proteomics data for gene sets related to sterol homeostasis and biosynthetic processes, steroid metabolic and biosynthetic processes, and isoprenoid biosynthetic processes. (**B**) Expression of genes involved in cholesterol biosynthesis and its regulation in the spinal cord and cerebral cortex of newborn (P0) control and *Pex2*$^{-/-}$ mice. (**C**) Whole tissue lysates were assessed by immunoblotting for phosphorylation of HMGCR at Ser872. Data are mean ± SD. Statistical analysis was performed using Student's t-test or Student's t-test with Welch's correction. *, $P<0.05$; **, $P<0.01$; ***, $P<0.001$; versus control mice.

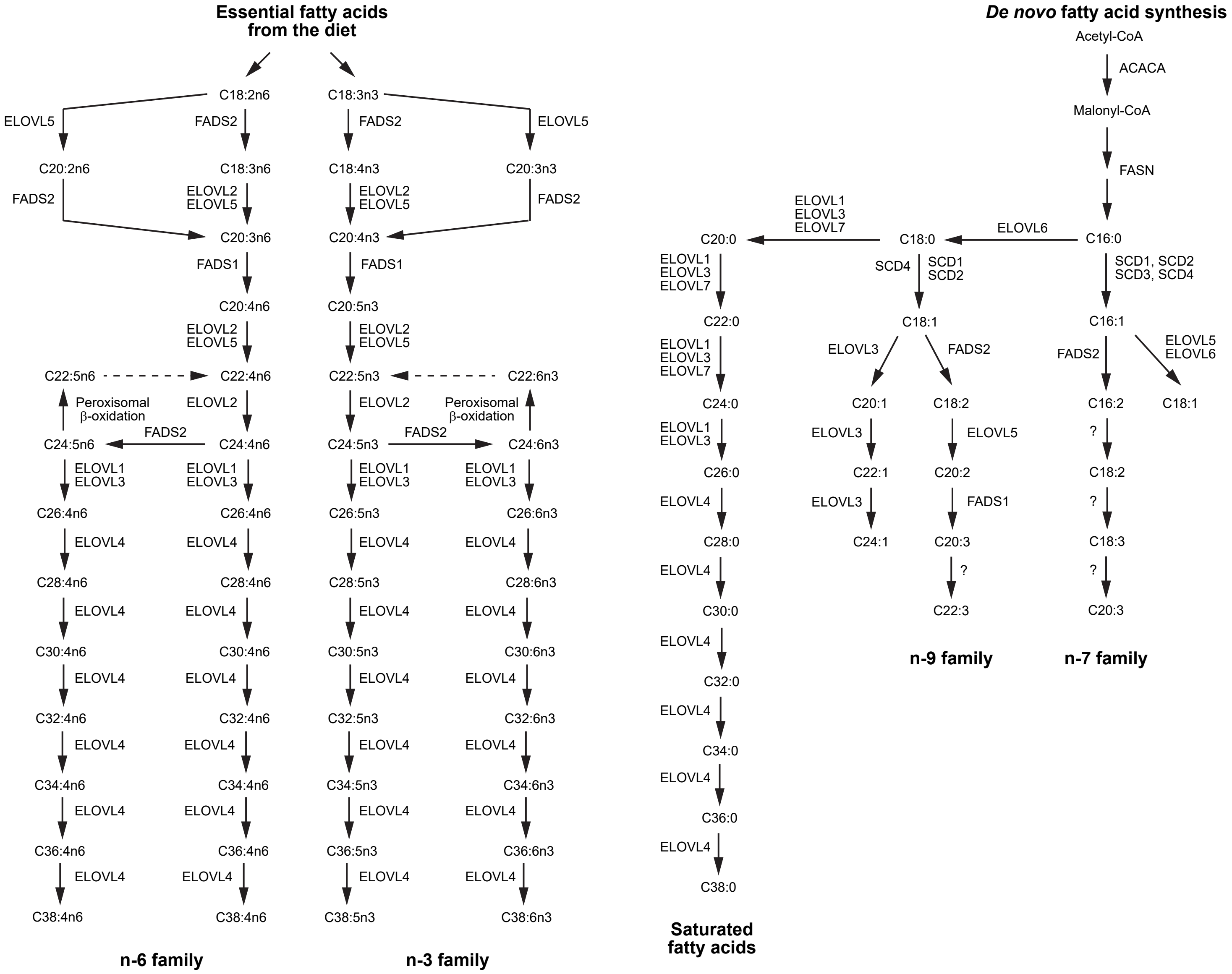

Supplemental Figure 5

Figure S5. Schematic representation of biosynthesis of very long-chain (VLC)-saturated and VLC-polyunsaturated fatty acids.

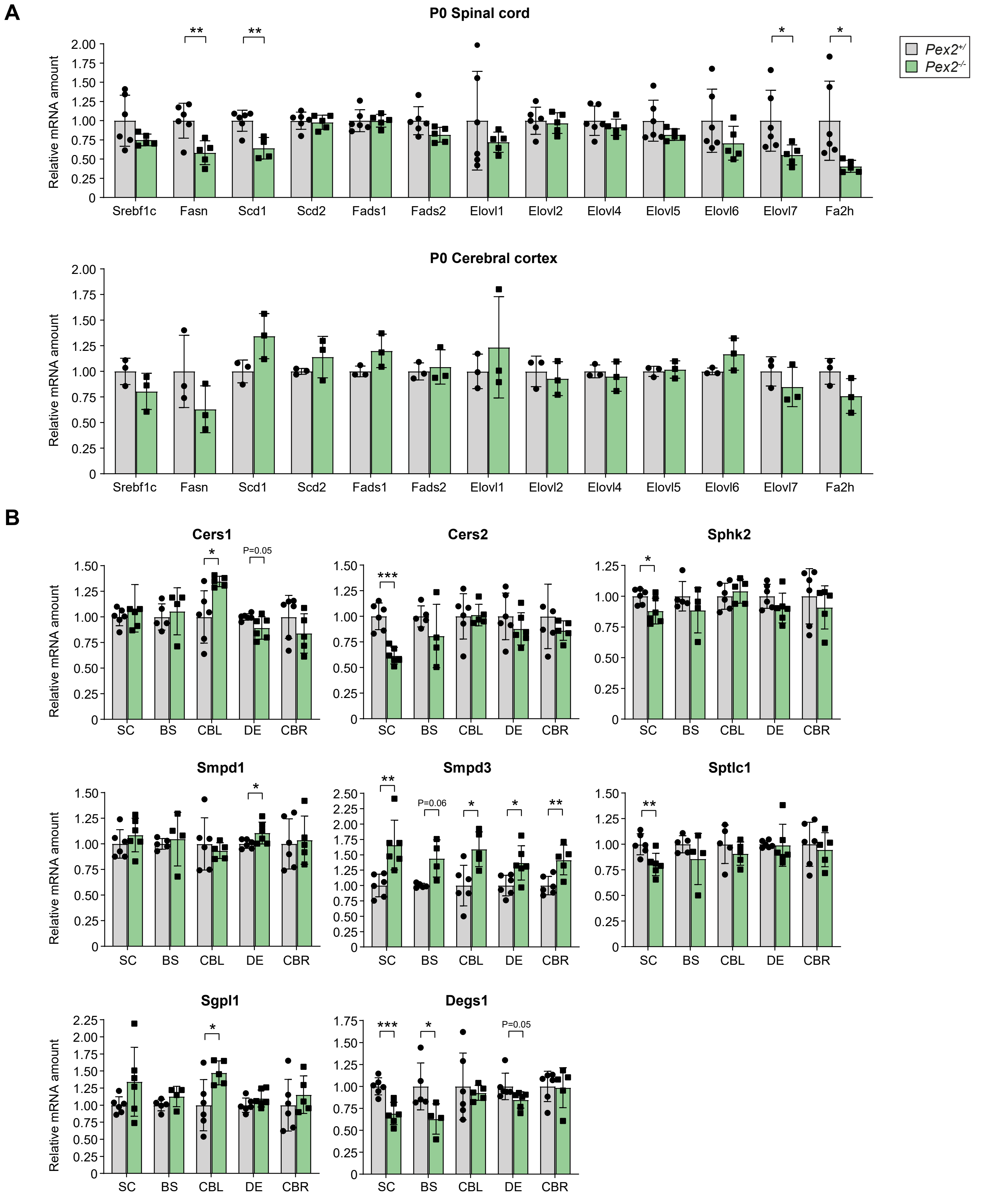

Supplemental Figure 6

Figure S6. (**A**) Expression of genes involved in fatty acid biosynthesis in the spinal cord and cerebral cortex of newborn (P0) control and *Pex2*$^{-/-}$ mice. (**B**) Expression of genes involved in sphingolipid metabolism in the CNS of P10 control and *Pex2*$^{-/-}$ mice. Data are mean ± SD. Statistical analysis was performed using Student's t-test or Student's t-test with Welch's correction. *, $P<0.05$; **, $P<0.01$; versus control mice.

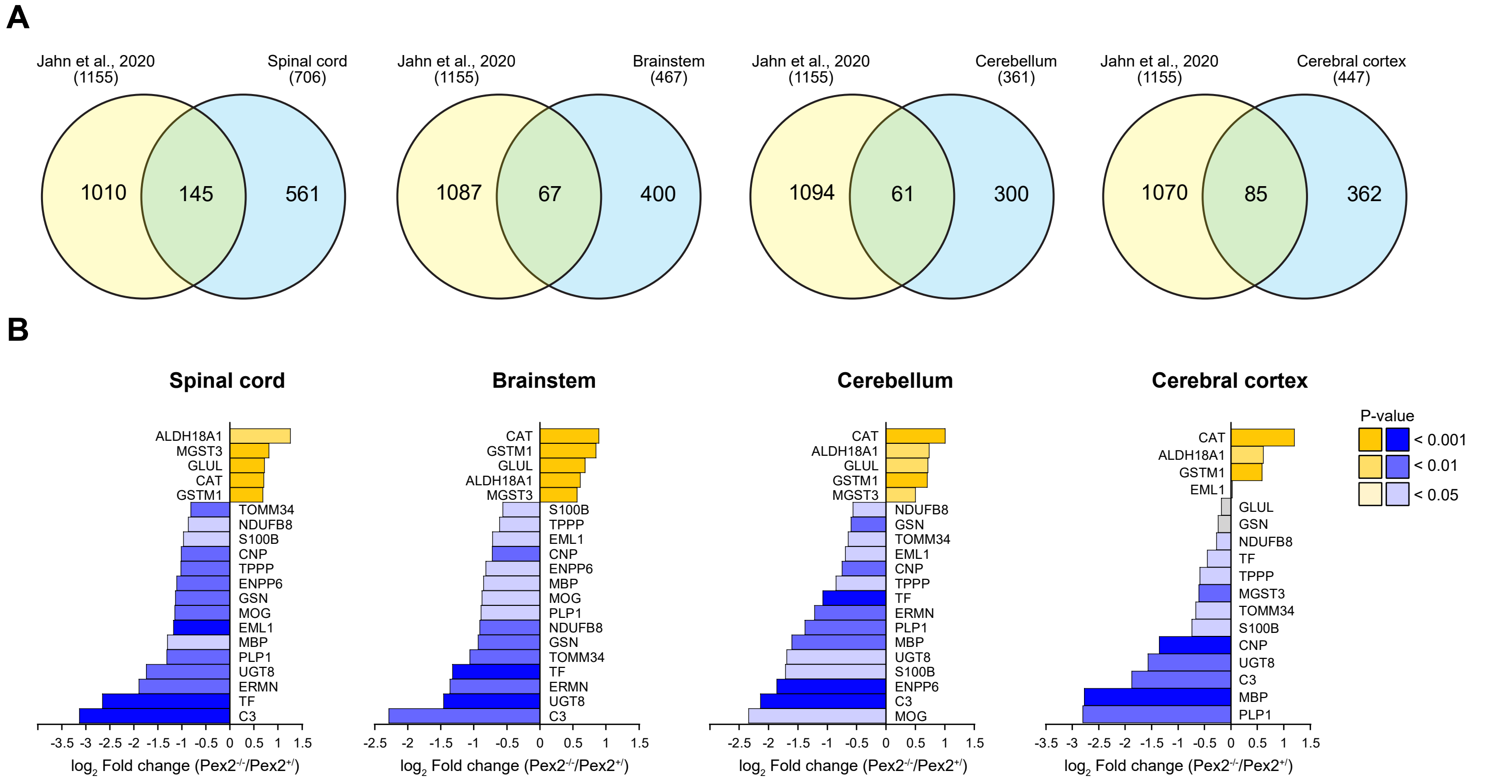

Supplemental Figure 7

Figure S7. (**A**) Venn diagrams showing the overlap of significantly altered proteins (cut-offs for log_2 fold change and P-value were set at ±0.5 and 0.05, respectively) in SC, BS, CBL and CBR of P10 control and *Pex2*$^{-/-}$ mice (blue) with the mouse myelin proteome (yellow) reported by Jahn et al. (Jahn et al., 2020). (**B**) Protein levels of 20 myelin-associated proteins that are significantly altered in SC, BS, and CBL. Blue bars: significantly downregulated proteins; yellow bars: significantly upregulated proteins; gray bars: no significant change.

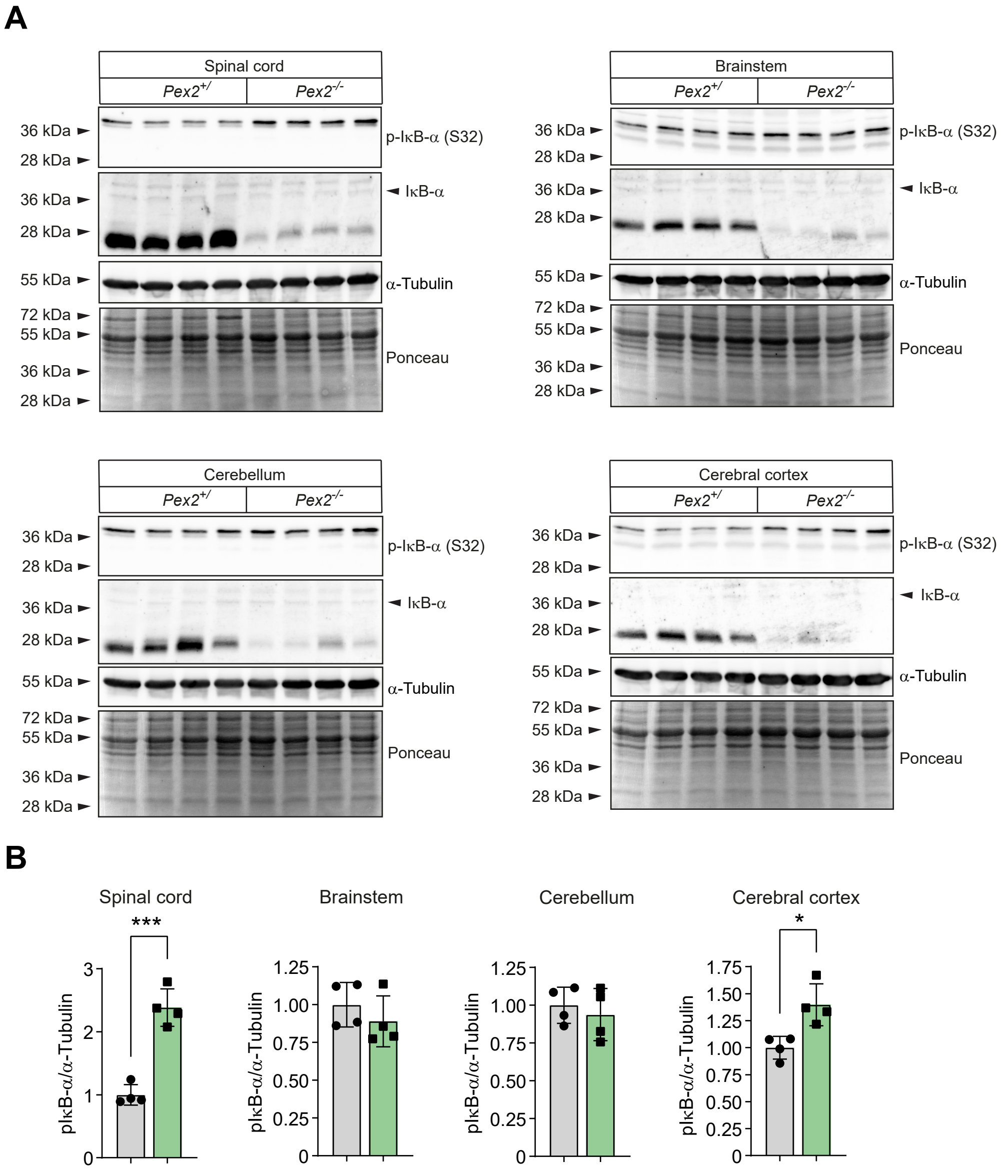

Supplemental Figure 8

Figure S8. Representative Western blots (A) and quantification (B) of p-IκB-α (S32) in the SC, BS, CBL, and CBR of P10 *Pex2*$^{-/-}$ mice. For quantification, p-IκB-α was normalized to α-tubulin. Protein ratios are expressed relative to that in control mice, which were arbitrarily defined as 1. *, P<0.05; ***, P<0.001; versus control mice.

Table S1. Antibodies for immunofluorescence (IF) and western blot analysis

Target	Host	Dilution		Source
		Western	IF	(Product number, company)
Catalase	Rabbit	1:8000	1:500	219010, Calbiochem; RRID:AB_2071738
CNP	Mouse	1:400		#MS-349-P1, Neomarkers; RRID:AB_61316
MBP	Mouse	1:1000	1:1000	#SMI99, Sternberger Monoclonals Incorporated
GFAP	Mouse	1:1000		G3893, Sigma-Aldrich; RRID:AB_477010
HMGCR	Rabbit	1:3000		Kovacs et al., 2004 [(1)]
p-HMGCR (Ser872)	Rabbit	1:1000		#09-356, Millipore; RRID:AB_1163451
α-Tubulin	Rabbit	1:5000		ab18251, Abcam; RRID:AB_2210057
p-AMPKα (Thr172)	Rabbit	1:1000		#2535, Cell Signaling Technology; RRID:AB_331250
AMPKα	Rabbit	1:1000		#2532, Cell Signaling Technology; RRID:AB_330331
p-AKT (Ser473)	Rabbit	1:1000		#4058, Cell Signaling Technology; RRID:AB_331168
AKT	Rabbit	1:1000		#9272, Cell Signaling Technology; RRID:AB_329827
p-RPS6 (Ser240/244)	Rabbit	1:1000		#2215, Cell Signaling Technology; RRID:AB_331682
RPS6	Rabbit	1:1000		#2217, Cell Signaling Technology; RRID:AB_331355
p-ERK1/2 (Thr202/Tyr204)	Rabbit	1:1000		#9101, Cell Signaling Technology; RRID:AB_331646
ERK1/2	Rabbit	1:1000		#9102, Cell Signaling Technology; RRID:AB_330744
p-IκB-α (Ser32)	Rabbit	1:1000		#9241, Cell Signaling Technology; RRID:AB_2566820
IκB-α (Clone 6A920)	Mouse	1:1000		IMG-127A, Imgenex; RRID:AB_1150527
Mouse IgG, HRP-linked	Goat	1:5000		Cat#401253, Millipore; RRID:AB_437779
Rabbit IgG, HRP-linked	Goat	1:5000		Cat#401393, Millipore; RRID:AB_437797
Rabbit IgG (H+L), Alexa Fluor 488 conjugated	Donkey		1:400	#A21206, Molecular Probes; RRID:AB_2535792
Mouse IgG (H+L), Texas Red conjugated	Horse		1:400	TI-2000, Vector Laboratories; RRID:AB_2336178

[(1)]Kovacs, W.J., Tape, K.N., Shackelford, J.E., Wikander, T.M., Richards, M.J., Fliesler, S.J., et al. (2004). Disturbed cholesterol homeostasis in a peroxisome-deficient *PEX2* knockout mouse model. *Mol Cell Biol* 24(1), 1-13 (2004).

Table S2. Quantitative real-time PCR primer.

Gene	Species	Forward primer	Reverse primer
Mvk	*Mus musculus*	5’-GGTGTGGTCGGAACTTCCC-3’	5’-CCTTGAGCGGGTTGGAGAC-3’
Dhcr24	*Mus musculus*	5’-CTCTGGGTGCGAGTGAAGG-3’	5’-TTCCCGGACCTGTTTCTGGAT-3’
Dhcr7	*Mus musculus*	5’-AGGCTGGATCTCAAGGACAAT-3’	5’-GCCAGACTAGCATGGCCTG-3’
Hmgcs1	*Mus musculus*	5’-AACTGGTGCAGAAATCTCTAGC-3’	5’- GGTTGAATAGCTCAGAACTAGCC-3’
Ldlr	*Mus musculus*	5’-TGACTCAGACGAACAAGGCTG-3’	5’-ATCTAGGCAATCTCGGTCTCC-3’
Lxra	*Mus musculus*	5’-CTCAATGCCTGATGTTTCTCCT-3’	5’-TCCAACCCTATCCCTAAAGCAA-3’
Abca1	*Mus musculus*	5’-AAAACCGCAGACATCCTTCAG-3’	5’-CATACCGAAACTCGTTCACCC-3’
Abcg1	*Mus musculus*	5’-CTTTCCTACTCTGTACCCGAGG-3’	5’-CGGGGCATTCCATTGATAAGG-3’
Abcg4	*Mus musculus*	5’-TGCTGACCACGCACCTAAAG-3’	5’-CCGCACGGAATAGGACAGT-3’
Apoe	*Mus musculus*	5’-CTGACAGGATGCCTAGCCG-3’	5’-CGCAGGTAATCCCAGAAGC-3’
Apod	*Mus musculus*	5’-TCACCACAGCCAAAGGACAAA-3’	5’-CGTTCTCCATCAGCGAGTAGT-3’
Cyp46a1	*Mus musculus*	5’-CAGCCGCTATGAGCACATCC-3’	5’-AGAAACACATCTTGGAGCACACG-3’
Srebf1c	*Mus musculus*	5’-GATGTGCGAACTGGACACAG-3’	5’-CATAGGGGGCGTCAAACAG-3’
Fasn	*Mus musculus*	5’-GGAGGTGGTGATAGCCGGTAT-3’	5’-TGGGTAATCCATAGAGCCCAG-3’
Elovl1	*Mus musculus*	5’-TCCAAAGCTACCCTCTGATGG-3’	5’-AGGGAGAGTATCACCAGTGAGA-3’
Elovl2	*Mus musculus*	5’-CCTGCTCTCGATATGGCTGG-3’	5’-AAGAAGTGTGATTGCGAGGTTAT-3’
Elovl3	*Mus musculus*	5’-TTCTCACGCGGGTTAAAAATGG-3’	5’-GAGCAACAGATAGACGACCAC-3’
Elovl4	*Mus musculus*	5’-GTCCTGAACGCGATGTCCA-3’	5’-GCGTGCTTATGCTTATCGTTG-3’
Elovl5	*Mus musculus*	5’-ATGGAACATTTCGATGCGTCA-3’	5’-GTCCCAGCCATACAATGAGTAAG-3’
Elovl6	*Mus musculus*	5’-AAGCAGTTCAACGAGAACGAA-3’	5’-CGTACAGCGCAGAAAACAGG-3’
Elovl7	*Mus musculus*	5’-CATCGAGGACTGTGCGTTTTT-3’	5’-CCAGGATGATGGTTTGTGGCA-3’
Fads1	*Mus musculus*	5’-AGCACATGCCATACAACCATC-3’	5’-TTTCCGCTGAACCACAAAATAGA-3’
Fads2	*Mus musculus*	5’-AAGGGAGGTAACCAGGGAGAG-3’	5’-CCGCTGGGACCATTTGGTAA-3’
Scd1	*Mus musculus*	5’-TTCTTGCGATACACTCTGGTGC-3’	5’-CGGGATTGAATGTTCTTGTCGT-3’
Scd2	*Mus musculus*	5’-GCATTTGGGAGCCTTGTACG-3’	5’-AGCCGTGCCTTGTATGTTCTG-3’
Ugt8	*Mus musculus*	5’-CTGGAGTTTCCAAGACCAACGC-3’	5’-CACCAGGACAAAGCCATGTTCC-3’
Mog	*Mus musculus*	5’-AGCTGCTTCCTCTCCCTTCTC-3’	5’-ACTAAAGCCCGGATGGGATAC-3’
Mag	*Mus musculus*	5’-CTGCCGCTGTTTTGGATAATGA-3’	5’-CATCGGGGAAGTCGAAACGG-3’
Tppp	*Mus musculus*	5’-AGGGCTGCTAAGAGGTTGTCA-3’	5’-GGTGTCCCCATGTACTGCAA-3’
Ermn	*Mus musculus*	5’-CTGAGACACTGAGCGGGAC-3’	5’-CAACCTTGTAGTATGCCTGGG-3’
Enpp6	*Mus musculus*	5’-CAGAGAGATTGTGAACAGAGGC-3’	5’-CCGATCATCTGGTGGACCT-3’
Eml1	*Mus musculus*	5’-TGAGAACCACCGTCAACAATG-3’	5’-GAGCTGGTCCTATTGATGCTTT-3’
Tspan2	*Mus musculus*	5’-TATCTGCTGCTCGGCTTCAAC-3’	5’-GTCCAAATGCAATAACGGCTG-3’
Gjc3	*Mus musculus*	5’-GGCGCTTCCTTCTTCCCAT-3’	5’-CCTGGCTGCCCTAAATGACA-3’
S100b	*Mus musculus*	5’-TGGTTGCCCTCATTGATGTCT-3’	5’-CCCATCCCCATCTTCGTCC-3’
Fa2h	*Mus musculus*	5’-CCACTTGGGGGAGAAGTATGA-3’	5’-TGGGGACACTATACCAGACAG-3’
Sox10	*Mus musculus*	5’-CGGACGATGACAAGTTCCCC-3’	5’-GTGAGGGTACTGGTCGGCT-3’
Aspa	*Mus musculus*	5’- ACATGGCTGCTGTTATTCATCC-3’	5’- GGGTACACGGTACAGTCTCCA-3’
Nkx2-2	*Mus musculus*	5’-AAGCATTTCAAAACCGACGGA-3’	5’-CCTCAAATCCACAGATGACCAGA-3’
Olig1	*Mus musculus*	5’-TCTTCCACCGCATCCCTTCT-3’	5’-CCGAGTAGGGTAGGATAACTTCG-3’
Olig2	*Mus musculus*	5’-TCCCCAGAACCCGATGATCTT-3’	5’-CGTGGACGAGGACACAGTC-3’
Ng2	*Mus musculus*	5’-GGGCTGTGCTGTCTGTTGA-3’	5’-TGATTCCCTTCAGGTAAGGCA-3’
Sox9	*Mus musculus*	5’-GAGCCGGATCTGAAGAGGGA-3’	5’-GCTTGACGTGTGGCTTGTTC-3’
Pdgfra	*Mus musculus*	5’-AGAGTTACACGTTTGAGCTGTC-3’	5’-GTCCCTCCACGGTACTCCT-3’
Ulk4	*Mus musculus*	5’-TAACTGGGATATACGGTCCAAGG-3’	5’-TGTGATCGCCTCGATAACAGG-3’
Myrf	*Mus musculus*	5’-CTAAAACGCCTCAGTAGCAGCTG-3’	5’-TGGAACCTGTCCTTCTGGCTGA-3’
Tnfa	*Mus musculus*	5’-CCCTCACACTCAGATCATCTTCT-3’	5’-GCTACGACGTGGGCTACAG-3’

Il6	*Mus musculus*	5’-TAGTCCTTCCTACCCCAATTTCC-3’	5’-TTGGTCCTTAGCCACTCCTTC-3’
Il1b	*Mus musculus*	5’-GCAACTGTTCCTGAACTCAACT-3’	5’-ATCTTTTGGGGTCCGTCAACT-3’
Phyh	*Mus musculus*	5’-CTCGGCCCCAACGATTGTAG-3’	5’-CCCTGGTGGTTTCACCTCC-3’
Cers1	*Mus musculus*	5’-CCACCACACACATCTTTCGG-3’	5’-GGAGCAGGTAAGCGCAGTAG-3’
Cers2	*Mus musculus*	5’-ATGCTCCAGACCTTGTATGACT-3’	5’-CTGAGGCTTTGGCATAGACAC-3’
Sphk2	*Mus musculus*	5’-CACGGCGAGTTTGGTTCCTA-3’	5’-CTTCTGGCTTTGGGCGTAGT-3’
Smpd1	*Mus musculus*	5’-TGGGACTCCTTTGGATGGG-3’	5’-CGGCGCTATGGCACTGAAT-3’
Smpd3	*Mus musculus*	5’-ACACGACCCCCTTTCCTAATA-3’	5’-GGCGCTTCTCATAGGTGGTG-3’
Sptlc1	*Mus musculus*	5’-ACGAGGCTCCAGCATACCAT-3’	5’-TCAGAACGCTCCTGCAACTTG-3’
Sgpl1	*Mus musculus*	5’-CTGAAGGACTTCGAGCCTTATTT-3’	5’-ACTCCACGCAATGAGCTGC-3’
Degs1	*Mus musculus*	5’-GAATGGGTCTACACGGACCAG-3’	5’-CGAGAAGCATCATGGCTACAA-3’
Trem2	*Mus musculus*	5’-CTGGAACCGTCACCATCACTC-3’	5’-CGAAACTCGATGACTCCTCGG-3’
Il34	*Mus musculus*	5’-TTGCTGTAAACAAAGCCCCAT-3’	5’-CCGAGACAAAGGGTACACATTT-3’
Csf1	*Mus musculus*	5’-GTGTCAGAACACTGTAGCCAC-3’	5’-TCAAAGGCAATCTGGCATGAAG-3’
Il10	*Mus musculus*	5’-GCTCTTACTGACTGGCATGAG-3’	5’- CGCAGCTCTAGGAGCATGTG-3’
Aif1	*Mus musculus*	5’-ATCAACAAGCAATTCCTCGATGA-3’	5’-CAGCATTCGCTTCAAGGACATA-3’
Cd68	*Mus musculus*	5’-TGTCTGATCTTGCTAGGACCG-3’	5’-GAGAGTAACGGCCTTTTTGTGA-3’
Cx3cr1	*Mus musculus*	5’-GAGTATGACGATTCTGCTGAGG-3’	5’-CAGACCGAACGTGAAGACGAG-3’
Cx3cl1	*Mus musculus*	5’-ACGAAATGCGAAATCATGTGC-3’	5’-CTGTGTCGTCTCCAGGACAA-3’
Tmem119	*Mus musculus*	5’-CCTACTCTGTGTCACTCCCG-3’	5’-CACGTACTGCCGGAAGAAATC-3’
Spp1	*Mus musculus*	5’-AGCAAGAAACTCTTCCAAGCAA-3’	5’-GTGAGATTCGTCAGATTCATCCG-3’
Tyrobp	*Mus musculus*	5’-GAGTGACACTTTCCCAAGATGC-3’	5’-CCTTGACCTCGGGAGACCA-3’
Clec7a	*Mus musculus*	5’-GACTTCAGCACTCAAGACATCC-3’	5’-TTGTGTCGCCAAAATGCTAGG-3’
Cst7	*Mus musculus*	5’-AGTCCCATGTCAGCAAAGCC-3’	5’-ATATAGAGTCCGCTTCAAGGCA-3’
Gfap	*Mus musculus*	5’-CCCTGGCTCGTGTGGATTT-3’	5’-GACCGATACCACTCCTCTGTC-3’
Serpina3n	*Mus musculus*	5’-ATTTGTCCCAATGTCTGCGAA-3’	5’-TGGCTATCTTGGCTATAAAGGGG-3’
Osmr	*Mus musculus*	5’-CATCCCGAAGCGAAGTCTTGG-3’	5’-GGCTGGGACAGTCCATTCTAAA-3’
Kdm5c	*Mus musculus*	5’-TGGGGATGTCAAGATGGAAT-3’	5’-CCACTGCCAAATTCTTTGG-3’
Kdm5d	*Mus musculus*	5’-ATATGCTCTCGTGGGGATGA-3’	5’-CCACTGCCAAATTCTTTGG-3’
